# Transcriptome Analysis in Chicken Cecal Epithelia upon Infection by *Eimeria tenella* In Vivo

**DOI:** 10.1371/journal.pone.0064236

**Published:** 2013-05-30

**Authors:** Aijiang Guo, Jianping Cai, Wei Gong, Hongbin Yan, Xuenong Luo, Guangfu Tian, Shaohua Zhang, Haili Zhang, Guan Zhu, Xuepeng Cai

**Affiliations:** 1 State Key Laboratory of Veterinary Etiological Biology, Lanzhou Institute of Veterinary Research, China Academy of Agricultural Sciences, Lanzhou, China; 2 Department of Veterinary Pathobiology, College of Veterinary Medicine and Biomedical Sciences, Texas A&M University, College Station, Texas, United States of America; 3 Adjunct Professorship, Institute of Genetics, College of Life Science, Zhejiang University, Hangzhou, China; Kyushu Institute of Technology, Japan

## Abstract

Coccidiosis, caused by various *Eimeria* species, is a major parasitic disease in chickens. However, our understanding on how chickens respond to coccidian infection is highly limited at both molecular and cellular levels. The present study employed the Affymetrix chicken genome array and performed transcriptome analysis on chicken cecal epithelia in response to infection for 4.5 days in vivo by the cecal-specific species *E. tenella*. By Significance Analysis of Microarrays (SAM), we have identified 7,099 probe sets with *q*-values at <0.05, in which 4,033 and 3,066 genes were found to be up- or down-regulated in response to parasite infection. The reliability of the microarray data were validated by real-time qRT-PCR of 20 genes with varied fold changes in expression (i.e., correlation coefficient between microarray and qRT-PCR datasets: *R* ^2^ = 0.8773, *p*<0.0001). Gene ontology analysis, KEGG pathway mapping and manual annotations of regulated genes indicated that up-regulated genes were mainly involved in immunity/defense, responses to various stimuli, apoptosis/cell death and differentiation, signal transduction and extracellular matrix (ECM), whereas down-regulated genes were mainly encoding general metabolic enzymes, membrane components, and some transporters. Chickens mustered complex cecal eipthelia molecular and immunological responses in response to *E. tenella* infection, which included pathways involved in cytokine production and interactions, natural killer cell mediated cytotoxicity, and intestinal IgA production. In response to the pathogenesis and damage caused by infection, chicken cecal epithelia reduced general metabolism, DNA replication and repair, protein degradation, and mitochondrial functions.

## Introduction

Chickens are one of the major sources of animal protein around the world. However, the poultry industry is consistently threatened by various viral, bacterial, and parasitic diseases. Among them, coccidiosis caused by a number of *Eimeria* species, including cecal *E. tenella*, is the most important parasitic disease that requires continuous prophylactic treatment. Live vaccines against certain coccidian species are available and well established for use in broiler breeds and layer hens. They are also increasingly accepted by the broiler industry, particularly with the attenuated vaccines [1], although the mainstream prophylactic treatments still use anti-coccidial drugs. The development of immune-based or other non-antibiotic therapeutics is highly appealing, considering the development of resistance in chickens against various anti-coccidian drugs (e.g, [2]–[6]), and the global trends against the use of prophylactic antibiotics in food animals. However, such an effort has been hampered by our limited understanding on how chickens respond to coccidian infections at molecular levels.

Previous studies have shown that coccidian infections produce both antibody and cell-mediated immune responses, in which cell-mediated immunity appeared to play a major role in disease resistance [7]. These observations might explain the unsuccessful attempts at developing subunit vaccines, despite several earlier attempts directed at identifying potential protective antigens for avian coccidiosis [8]–[11]. There have been other studies by real-time quantitative reverse transcription-polymerase chain reaction (qRT-PCR) on the fluctuated expression of immune-relevant genes in various chicken tissues or cells [12]–[14]. More recently, an intraepithelial lymphocyte (IEL) cDNA microarray has been developed and used to study gene expression profile changes in immune cells in response to *E. maxima, E. acevulina,* and *E. tenella* infections [15]–[19].

Two whole genome microarrays have been developed (i.e., Affymetrix chicken genome array and the Agilent platform-based 44 K chicken oligo microarray), which permit global gene expression profiling analysis [20], [21]. In the present study, we used the Affymetrix chicken array and performed global transcriptome analysis on chicken cecal mucous membranes in response to *E. tenella* infection in vivo. We observed complex responses, mainly elevated expression of genes associated with the immunological responses and regulation, signal transduction, cell death and cell differentiation.

## Results and Discussion

### General Characterization of the Lanzhou-1 Strain of *E. tenella*

Chickens may be infected by up to 7–9 different *Eimeria* species specialized at different portions of the gut, in which *E. tenella* is one of the most pathogenic species that exclusively occupies the cecum [22]–[24]. All *Eimeria* species infect the intestinal epithelial cells with the potential to fully occupy the infection sites if appropriate control measurements are not given. The present study focused on evaluating gene expression changes in cecal epithelia after *E. tenella* infection in chickens for 4.5 days, corresponding to the most damaging developmental stage of second generation of merogony.

**Figure 1:**
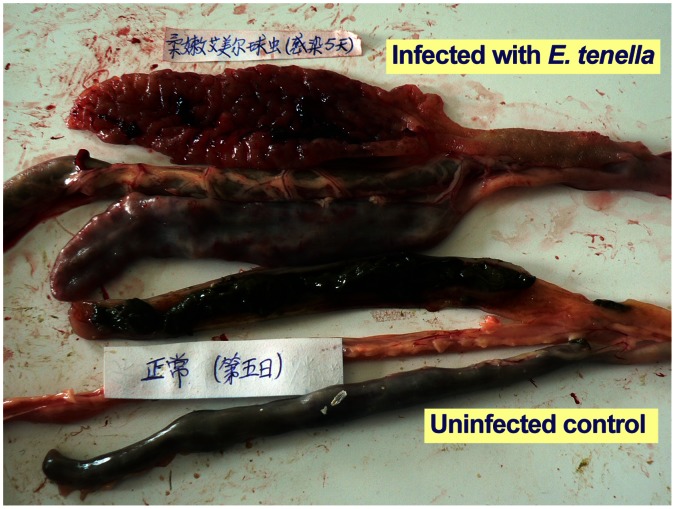
Typical cecal lesion (score = 3) in a chicken infected with the Lanzhou-1 strain of *Eimeria tenella* (10^5^ oocyst/bird) on day 5 post-infection (pi) in comparison with that from an uninfected bird.

**Figure 2:**
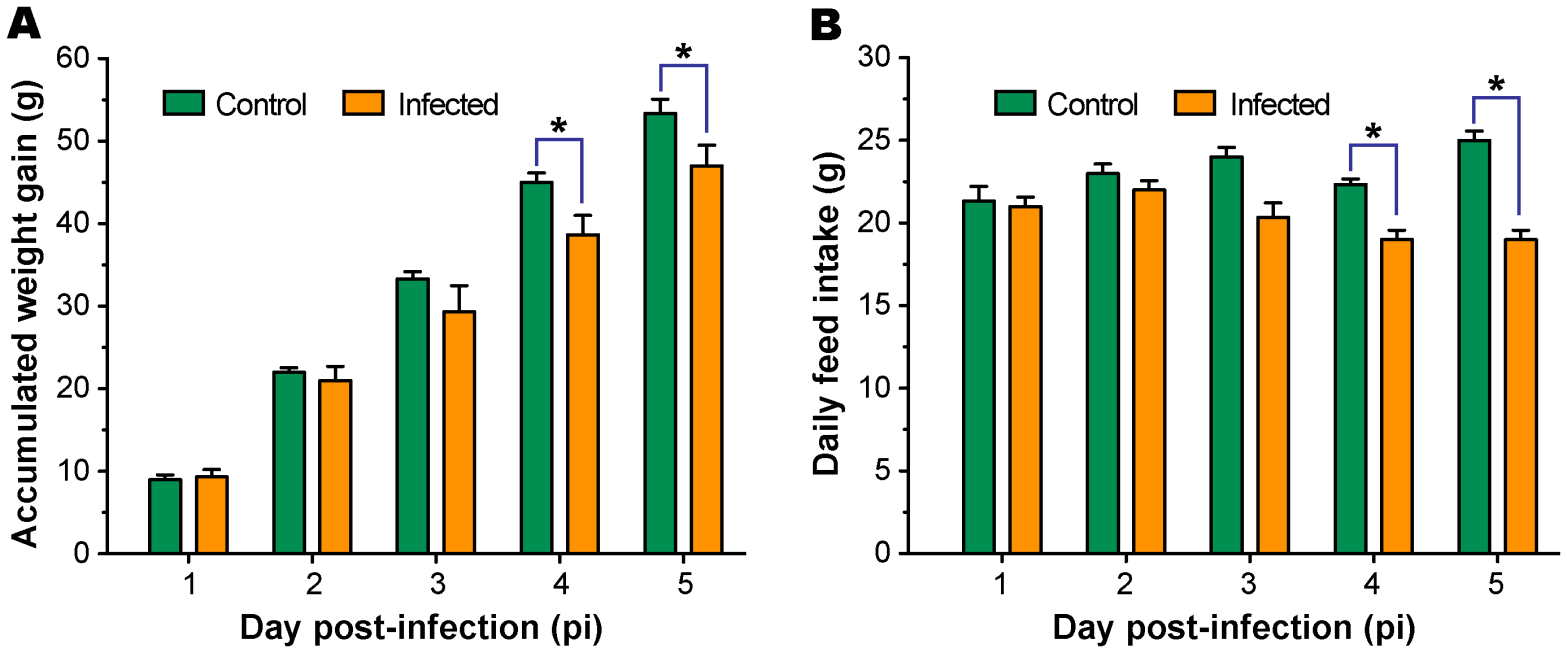
Comparison of accumulated weight gains (A) and daily feed intake (B) between chickens infected with the Lanzhou-1 strain of *Eimeria tenella* (10^5^ oocysts/bird) and uninfected controls. Asterisks indicate statistically significant differences between infected and uninfected groups (*p*<0.05).

The study used Lanzhou-1 strain of *E. tenella* that was locally isolated from a farm in Lanzhou, China. Chickens infected with this strain (10^5^ oocysts/10-day old bird) started to show classic clinical symptoms on day 3 to 4 post-infection (pi), which included droopiness, listlessness, ruffled feathers, reduced weight gain and loss of appetite. Bloody diarrheas typically started on day 5 pi, but it occurred on day 4 pi in some birds. Chicken ceca became thickened, shortened and filled with blood. Based on a 0 to 4 scoring system [25], the lesions could be scored at 4 in all infected birds on day 7 pi, or at ∼3 in day 4.5 pi (Figure 1). The reductions in both weight gain and feed intake were statistically significant on day 4 pi, and thereafter (Figure 2). In a separate experiment with 10 birds infected for 8 days, one death was observed on each of the days 5 to 7 pi (i.e., 30% death in 7 days pi). However, deaths did not occur in birds used in this study. These observation indicate that this strain of *E. tenella* is highly pathogenic to chickens and could cause symptoms characteristic of cecal coccidiosis.

### Differentially Expressed Genes in Chicken Cecal Epithelia upon *E. tenella* Infection

**Figure 3:**
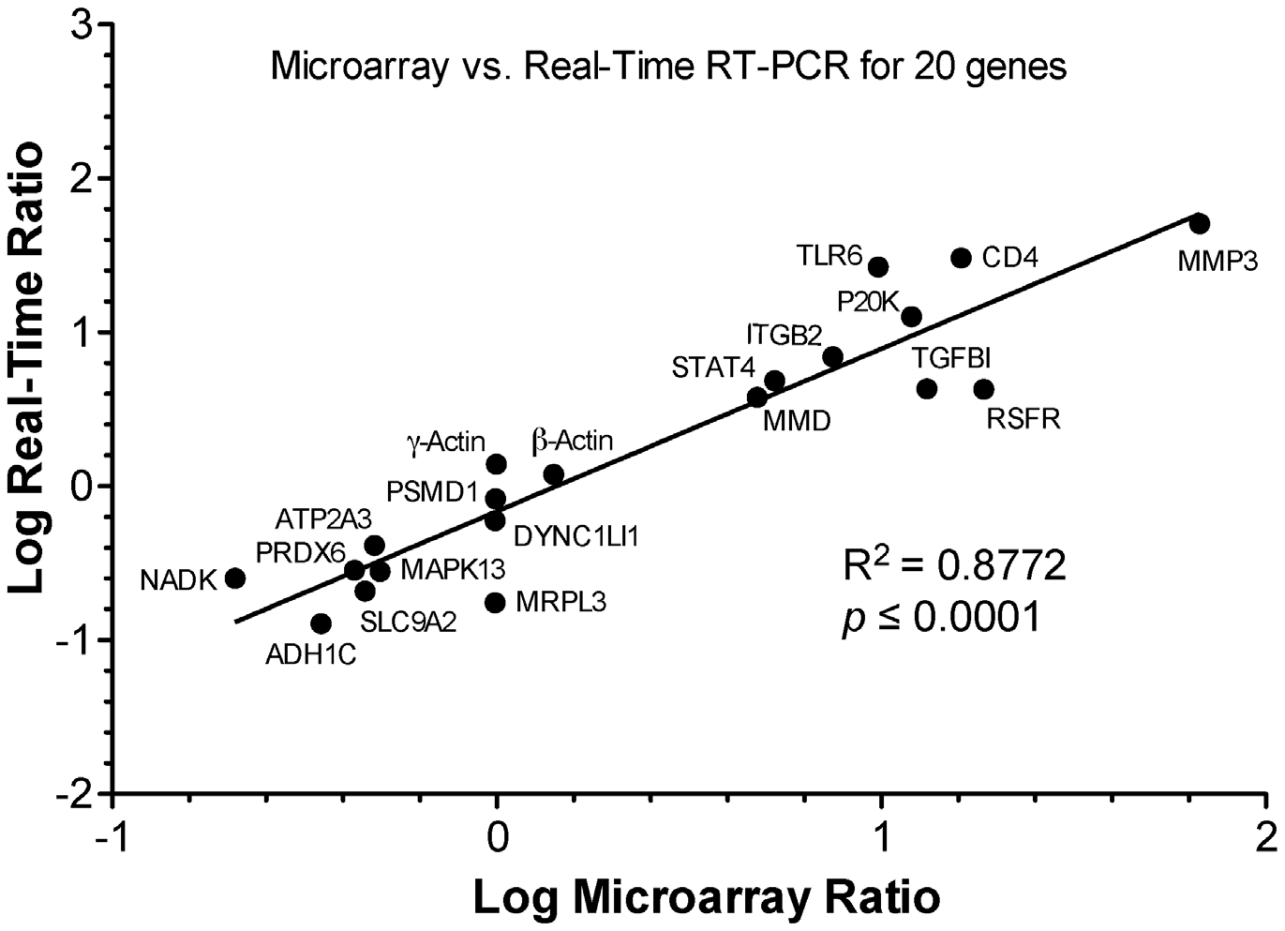
Correlation between microarray and qRT-PCR data on 20 selected genes with various fold changes as plotted by the logarithm of ratios of means between infected and uninfected samples. Primers are listed in Table S5.

Both infected and uninfected samples included three biological replicates, with cecal epithelia pooled from 4 chickens for RNA extraction. Using Significance Analysis of Microarrays (SAM) software, we have identified 7,099 genes (probe sets) from a total of 16,391 genes in the array with *q*-values at <0.05, in which fold changes ranged from 1.112 to 67.335 fold in up-regulated (n = 4,033) and from 0.916 to 0.099 fold in down-regulated genes (n = 3,066) upon infection (Figure S1, also see Table S1 for a complete list). Among the up-regulated, 1,355 or 831 probe sets had >2 or >3 fold changes, while in the down-regulated, 538 or 57 sets had >2 or >3 fold changes (i.e., ratios between infected and uninfected specimens at <0.5 or <0.3333), respectively. Parasites in infected specimens had no or little effect on the microarray data as separate hybridization of chips using probes prepared from RNA isolated from pure *E. tenella* merozoites only produced background or near background signals (data not shown). The reliability of the microarray data were validated by real-time qRT-PCR of 20 genes with varied fold changes in expression, in which no conflicts were observed between the real-time and microarray datasets (Figure 3). The two datasets had a good correlation coefficient (*R* ^2^ = 0.8773, *p*<0.0001).

The top 10 up-regulated genes were associated with extracellular matrix (ECM) (matrix metallopeptide 3, fibronectin 1 and lumican), immunity/defense (lymphatic vessel endothelial hyaluronan receptor 1 and chemokine [C-C motif] ligand 17), signal transduction across membrane (ecotropic viral integration site 2A and POU class 2 associating factor 1), cytoskeleton/smooth muscle contraction (caldesmon 1 and myosin light chain kinase), and heme degradation (biliverdin reductase A) (Table 1). Six of the top 10 down-regulated genes were involved in general metabolism (carbonic anhydrase VII, calbindin, NAD kinase, UDP glucuronosyltransferase, beta-galactoside alpha-2,3-sialyltransferase, and succinate-CoA ligase), along with three transporters (two solute carrier family proteins and lysosomal H^+^ transporter), and twinfilin, which is an actin-binding protein and actin assembly inhibitor [26].

**Table 1:** List of top 20 differentially expressed genes.*

Functional Group		Signal Ratio	Protein ID	Gene description and note
**Top 10 up-regulated**				
Extracellular matrix	67.33		XP_417175	Matrix metallopeptidase 3 (stromelysin 1, progelatinase)
Immunity/defense	32.61		XP_420971	Lymphatic vessel endothelial hyaluronan receptor 1
Cytoskeleton	29.98		NP_989489	Caldesmon 1 [Smooth muscle contraction]
Metabolism	29.58		XP_418872	Biliverdin reductase A [Heme degradation]
Immunity/defense	29.43		XP_414018	Chemokine (C-C motif) ligand 17
Cytoskeleton	23.41		NP_990790	Myosin, light chain kinase [Smooth muscle contraction]
Extracellular matrix	23.20		XP_421868	Fibronectin 1
Signal transduction	21.40		NP_001074362	Ecotropic viral integration site 2A
Signal transduction	21.372		NP_989506	POU class 2 associating factor 1 [Negative regulation of NF-kappaB transcription factor activity]
Extracellular matrix	21.079		XP_421599	Lumican [laminin-binding]
**Top 10 down-regulated**				
Transporter		0.26	XP_415945	Solute carrier family 26, member 3
Metabolism		0.26	XP_414152	Carbonic anhydrase VII
Transporter		0.25	NP_001008455	ATPase, H+ transporting, lysosomal 38 kda, V0 subunit D2
Cytoskeleton		0.23	NP_001025910	Twinfilin, actin-binding protein, homolog 1 (Drosophila) [Actin assembly inhibitor]
Metabolism		0.22	NP_990844	Calbindin 1, 28 kda; calcium-binding
Metabolism		0.21	NP_001026041	NAD kinase
Metabolism		0.19	XP_420613	UDP glucuronosyltransferase 2 family, polypeptide A3
Metabolism		0.19	XP_417860	ST3 beta-galactoside alpha-2,3-sialyltransferase 4
Transporter		0.17	XP_416918	Solute carrier family 9 (sodium/hydrogen exchanger), member 2
Metabolism		0.10	NP_001006141	Succinate-CoA ligase, GDP-forming, beta subunit

* Four unknown genes were excluded from the list. Also see Table S1 for a complete gene list with probe set numbers.

### Gene Ontology Analysis and Pathway Mapping

**Figure 4:**
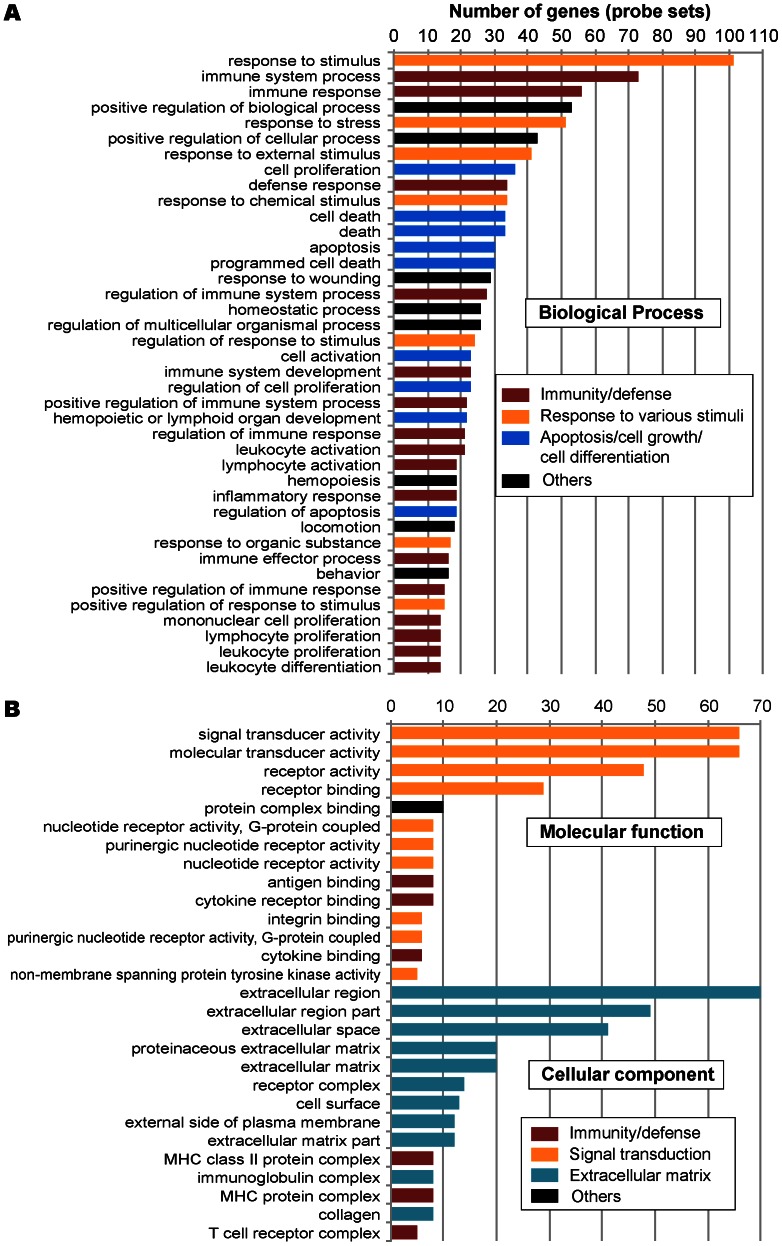
Gene ontology (GO) analysis of up-regulated genes in chicken cecal epithelia in response to *Eimeria tenella* infection. (**A**) Top GO terms in biological process. (**B**) Top GO terms in molecular function and cellular components.

**Figure 5:**
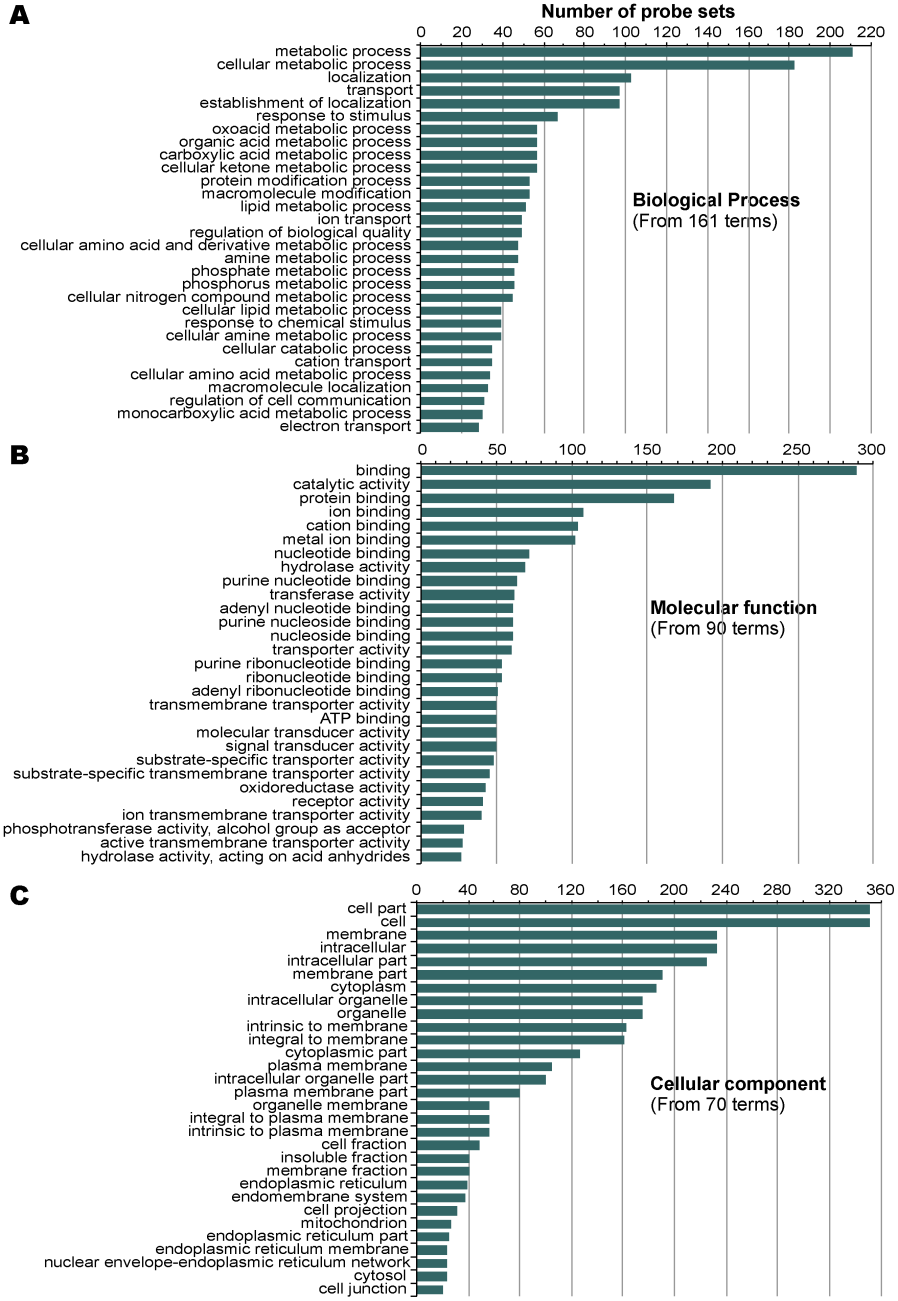
Gene ontology (GO) analysis of down-regulated genes in chicken cecal epithelia in response to *Eimeria tenella* infection. (**A**) Top GO terms in biological process. (**B**) Top GO terms in molecular function. (**C**) Top GO term in cellular components.

**Figure 6:**
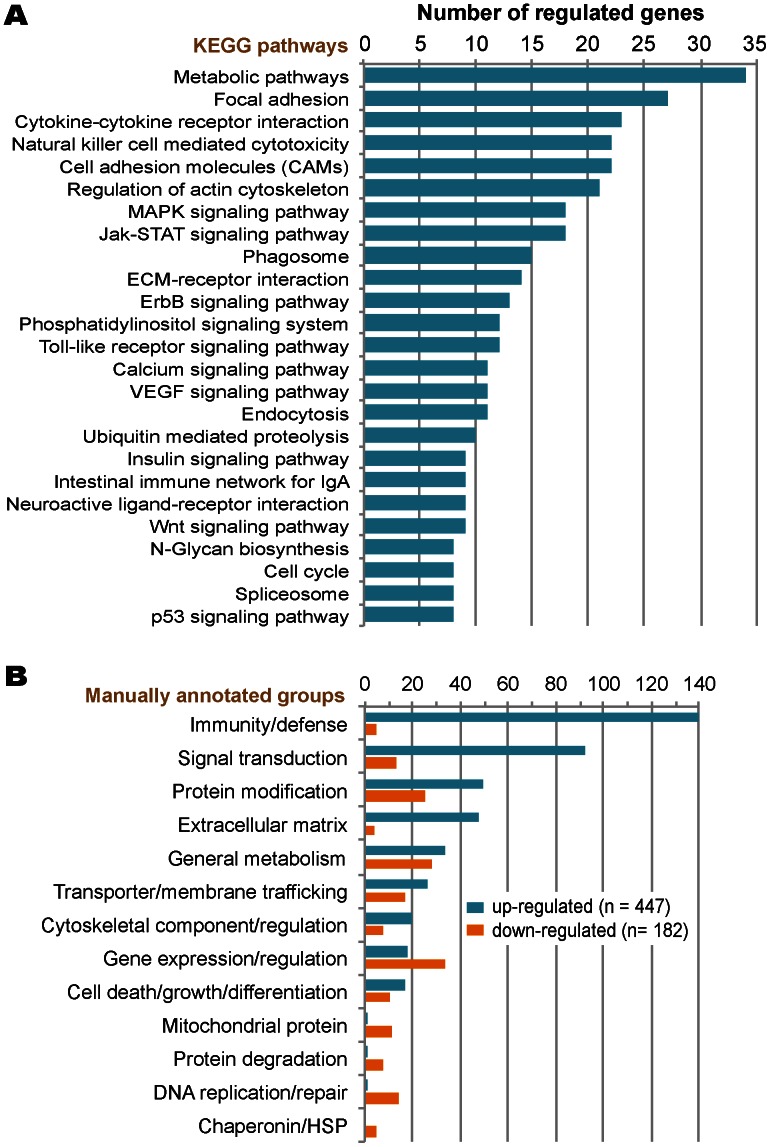
Major pathways and functional clarifications of significantly regulated genes in chicken cecal epithelia in response to *Eimeria tenella* infection. (**A**) KEGG metabolic pathway mapping of regulated genes. (**B**) Manual annotation of regulated genes by functional groups. Cutoff values: *q*<0.05, fold change >3 for up-regulated and >2 for down-regulated genes.

Due to the disproportional numbers between up- and down-regulated genes, we selected 831 up-regulated genes with >3X fold changes and 538 down-regulated ones with >2X fold changes in our gene ontology (GO) and pathway mapping analyses. Using updated GO terms maintained by the AgriGO project (http://bioinfo.cau.edu.cn/agriGO/) and a Singular Enrichment Analysis (SEA) with both minimal *p*-value (Fisher) and false discovery rate (FDR) (Hochberg) *q*-value set at 0.05, we were able to map 179 and 321 GO terms to the up- and down-regulated genes, respectively (Figure 4 and Figure 5, also see Table S2 for a complete list of the terms associated with information of probe sets). In KEGG pathway mapping, 1,046 genes could be assigned gene identification numbers with the National Center for Biotechnology Information (NCBI Gene ID), in which 450 genes could be mapped into various pathways at the Kyoto Encyclopedia of Genes and Genomes databases (http://www.genome.jp/kegg/). A total of 113 KEGG pathways were mappable by at least one gene and 44 pathways by at least 5 genes (Figure 6A, also see Table S3 for a complete list and Figure S2 for the top 20 color-coded KEGG pathways).

Additionally, since the chicken genome is not fully annotated, a large number of regulated genes could not be mapped into specific pathways in either the AgriGO or KEGG databases. Therefore, we performed a “rough” manual annotation of all infection-regulated genes by assigning them to major functional groups. We were able to classify 629 out of the 1,369 genes into 13 major groups (Figure 6B, also see Table S4 for a complete list). The manual classification provided a new look at gene expression profiles that might be obscured by GO and KEGG pathway mapping, particularly among the down-regulated members involved in DNA replication and repair, mitochondrial metabolism, protein degradation, and a subset of chaperonins and heat shock proteins (HSPs).

All analyses revealed the same trend that major elevated expression of genes in infected chicken cecal epithelia during the second generation of merogony were associated with immunity and defense (e.g., cytokine-cytokine receptor interaction, phagosome, natural killer cell mediated cytotoxicity and toll-like receptors signaling pathway), signal transduction (e.g., MAPK, Jak-STAT, ErbB, and phosphatidylinositol signaling pathways), ECM (e.g., focal adhesion, cell adhesion and ECM-receptor interaction), and cell cycle-associated pathways such as those involved in apoptosis and cell differentiation. A higher number of up-regulated genes, particularly those involved in various immunological responses, than down-regulated ones in infected specimens were likely a result of the “new” population of immune cells recruited to the infection site, rather than by the “original” population of epithelial cells. These observations were in congruence with earlier observations that interferon-γ and various interleukins mediating cellular immunity plays an important role in response to the coccidian infections [12], [15], [27]–[31].

Many regulated pathways were interconnected or functionally overlapped, such as cytokine/cytokine receptor interaction and Jak-STAT signaling pathway, and the recruiting of immune and other types of cells in the adhesion molecule interactions (e.g., MHC-I, MHC-II or ITGB2 in dendritic cells that interact with CD8, CD4 or CD226 in T cells). Notably, many genes encoding components within the intestinal immune network for IgA production were highly elevated, including class II MHC in dendritic cells, CD28, CD40L and ICOS in CD4^+^ T cells, BAFF in the epithelia, as well as MHC in B2 cells and integrin α4β7 in IgA^+^ B cells. The role of IgA in immuno-protection against *Eimeria* infection is still debatable. Although significant levels of IgA were consistently observed in the gut mucosa in response to *Eimeria* infection, several studies have shown that both chemical and hormonal bursectomy could not diminish the development of protective immunity against *Eimeria* reinfection [28], [32]. All together, we speculate that IgA production may not be essential for chickens to develop protective immunity, but may play an additive role in combating coccidian infection.

Another notable change was the elevated expression of several genes encoding the coagulation cascade, such as coagulation factors VII (probe set: Gga.3468.1.S1_at), coagulation factor X (Gga.514.1.S1_at), and tissue factor pathway inhibitor (Gga.11741.1.S1_a_at), which was apparently associated with the typical pathology (i.e., severe bleeding) caused by infection.

In contrast to immunity and defense associated pathways, general metabolism was significantly decreased in the infected cecal epithelia. Also down-regulated were genes involved in protein degradation, DNA replication and repair, and mitochondrial functions (Figure 6B), suggesting that infected cecal epithelia suppressed cell growth associated activities after devoting a significant amount of resources to muster a complex immunological defense against the severe coccidian infection. However, the reductions in general metabolism may also be associated with the significantly reduced feed intake and the 12-h fasting prior to the collection of tissues, as these would unavoidably decrease the availability of nutrients in infected chickens, affecting their general metabolism (Figure 2B).

Genome microarray and recently developed RNA-Seq technologies are powerful tools for transcriptome analysis of host cell responses against infections. However, only a limited number of studies were devoted to the analyses of global gene expression profiling in chickens in response to coccidian infections. In a pioneering study in 2003, a cDNA microarray containing 400 unique chicken genes was developed and used to study the gene expression changes in intestinal intraepithelial lymphocytes (IELs) in response to the primary and secondary infections by *E. acervulina* and *E. maxima* [15]. The cDNA microarray was then significantly expanded to contain 9,668 genes, which was reapplied to study the infections of *E. acervulina* and *E. maxima* [18], [33], and later extended to include *E. tenella* [19]. This serial of studies using cDNA microarray focused on the gene expressions in isolated intestinal lymphocytes, and have identified a large number of genes that were significantly regulated by coccidian infections, including those involved in “Diseases and Disorder” and “Physiological System Development and Function”, along with 16 intracellular signaling pathways. In chickens infected with *E. tenella*, the major regulated pathways were those involved in “Hematological system development and function”, “Hematopoiesis”, “Connective tissue development and function”, and “Digestive system development and function” [19]. It was noticeable that two of the four functional categories were related to hematopoiesis, which was in congruent with the data obtained in this study.

Although Affymetrix and Agilent microarrays have been recently developed to cover the entire chicken genome (e.g. [20], [21]), these two less-biased platforms were previously not used to study the coccidian infections. The present study employed Affymetrix chicken microarray to study gene expressions regulated by *E. tenella* infection in cecal epithelial cells, rather than focusing on a single specific type of cells, thus providing a more comprehensive snapshot of the global gene expression changes in both infected epithelial cells and immune cells recruited to the infection sites. However, more comprehensive analyses with multiple time points of infection, various parasite strains and chicken breeds with distinguished phenotypes, as well as with isolated cell subpopulations are still needed to fully understand the molecular interactions and pathogenesis at the infection sites.

## Materials and Methods

### *Eimeria tenella* Infection in Chickens

The Lanzhou-1 strain of *E. tenella* was originally isolated in the field in Lanzhou, China and maintained in the Lanzhou Veterinary Research Institute. Parasite oocysts were harvested, sporulated and stored as previously described [34]. One-day old specific pathogen-free (SPF) ISA Brown chickens were purchased from Xigu Farms, Lanzhou and housed in an oocyst-free animal house. Animals were given free access to feed and water, and constant light was provided during the entire experimental period. When the animals were 10-days old, 35 healthy chickens were randomly divided into two groups consisting of 20 and 15 chickens. Group 1 chickens (n = 20) were orally inoculated with 1.0×10^5^ sporulated oocysts (<3 months old). Chickens in group 2 (n = 15) receiving no oocysts were used as uninfected controls. To avoid contamination, the two groups of chickens were housed in two separate rooms, and care and feeding were performed by different personnel. All experimental protocols were approved by the Committee for the Care and Use of Experimental Animals at Lanzhou Veterinary Research Institute, China.

### Isolation of Chicken Cecal Epithelia

All chickens were fasted for 12 h prior to collecting ceca. Twelve birds in each group were killed by cervical dislocation at 4.5 days post-infection (pi). A protocol adapted from previous reports was used to isolated cecal epithelial cells from infected and uninfected chickens [35], [36]. Briefly, cecal mucus and gut contents were first gently scraped with glass microscope slides and discarded. Samples from 4 of the 12 chickens were pooled together to form 3 biological replicates in each group. Pooled samples were suspended in 5 mL solution containing 40 mM Tris-HCl (pH 7.4), 4 mM KCl, 3 mM MgCl, and 0.25 M sucrose, and homogenized with a Dounce homogenizer with a loose fitting pestle (Yongcheng Company, Guangzhou, China). Epithelial cells were enriched by passing consecutively through 212, 106, 61 and 45 µm sieves. Cells retained in 45 µm sieves were collected by centrifugation at 500×*g* for 10 min. Collected cells were examined microscopically and counted with a hemocytometer which revealed ∼90% epithelial cells, plus a small portion of other cells including immune cells.

### Preparation and Purification of Merozoites from Infected Chickens

Parasite merozoites were isolated from four infected group 1 chickens (5 days pi) using a slightly modified protocol as previously described [37]. Briefly, chicken cecal epithelial layers were collected by scraping with glass slides and homogenized using a motar and pestle in a solution containing 120 mM NaCl, 20 mM Tris-HCl (pH 7.4), 3 mM K_2_HPO_4_, and 1 mM CaC1_2_. The mixture was further homogenized in 50 mL loose fitting pestles, and sieved through a 350 µm sieve. The filtrates were centrifuged at 1000×*g* for 10 min. The pellet was washed once with PBS (pH 7.4) and resuspended in 50 mL PBS. The solution containing merozoites was passed through a series of 212, 106 and 61 µm sieves. Final filtrate contained merozoites with >90% purity as assessed microscopically with a hemocytometer. The remaining portion was red blood cells and epithelial debris.

### Probe Preparation and Hybridization

Isolation of total RNA, preparation of cRNA probes, hybridization and chip scans were performed by CapitalBio (Beijing, China). Briefly, total RNA was first isolated using a Trizol RNA isolation kit (Invitrogen, USA) and then purified using an RNeasy Mini Kit (Qiagen, Germany) according to the manufacturers’ instructions. The quality and quantity of RNA were assessed by formaldehyde agarose gel electrophoresis and spectrophotometry. Biotin-labeled double-stranded cRNA probes were prepared from total RNA (2 µg/sample) using a MessageAmpTM II aRNA Amplification Kit. The resulting biotin-tagged cRNA samples were fragmented to 35–200 bases in length according to the protocols from Affymetrix. The fragmented cRNA was hybridized to GeneChip Chicken Genome Arrays (GPL3213, Affymetrix, Santa Clara) that contained 16,392 probe sets, representing 32,773 transcripts and 28,000 genes.

Seven Affymetrix chicken genome array chips were used, including three biological replicates in each of the infected and uninfected groups, plus one for the merozoite specimen as an additional control. Hybridization was performed at 45°C with rotation for 16 h in an Affymetrix GeneChip Hybridization Oven 640. The arrays were washed and then stained with streptavidin- phycoerythrin on an Affymetrix Fluidics Station 450, followed by scanning on a GeneChip Scanner 3000.

### Microarray Data Analysis

Acquired signal intensity data were analyzed using GeneChip Operating software (GCOS 1.4), in which the scanned images were assessed by visual inspection, then median signals were read with default settings and saved as CEL files. Raw signal intensities were first normalized by an invariant-set normalization algorithm using a DNA-chip analyzer (dChip version 2008) [38], [39].

Significance Analysis of Microarrays (SAM) was used to identify genes that were differentially expressed between infected and uninfected samples. Genes with >2 fold changes and both false discovery rate (FDR, *q*) and Wilcoxon Rank-Sum test significance level (*p*) <0.05 were considered as significantly regulated. However, due to the disproportionally higher number of up-regulated genes than down-regulated ones (Figure S1), and to better balance the number of genes in both directions, we selected genes with >3 fold changes in the up-regulated and >2 fold changes in the down-regulated for subsequent gene ontology (GO) analysis and KEGG pathway mapping. Gene ontology (GO) analysis was performed using the AgriGO system with *p*-values and FDR values calculated by Fisher and Hachburg algorithms, respectively (http://bioinfo.cau.edu.cn/agriGO/). GO terms were considered as significantly enriched with both *p*-values and FDR at <0.05. KEGG pathway mapping was performed using KEGG mapper (http://www.genome.jp/kegg/tool/map_pathway2.html). Manual annotation was performed by visual scanning the definitions of all genes, including those unmapped in AgriGO and KEGG databases, and assigning them into major functional groups as previously described [40]–[42].

### Quantitative Real-time RT-PCR

Quantitative real-time RT-PCR (qRT-PCR) was performed for 20 genes with different levels of differential expression (10 up-regulated, 6 down-regulated, and 4 with no or little changes in microarray data). The same RNA extracts used for microarray analyses were further digested with DNase I at 37°C for 15 min to remove contaminating DNA. RNA quantities and qualities were determined by spectrophotometer and electrophoresis, judged by the presence of intact 28S and 18S rRNA bands at intensity ratio of ∼2∶1. Reverse transcription was performed using a PrimeScript RT kit (Takara Biotechnology, Dalian, China). Subsequent PCR amplification reactions were conducted using SYBR Premix ExTaq II (Takara Biotechnology) using primers listed in Table S5. Real-time PCR was carried out in a final volume of 25 µL containing 0.4 µm of each primer, 12.5 µL of 2 × SYBR Premix ExTaq and varied concentrations of cDNA templates. Rotor-Gene Q real-time PCR detection system (Qiagen, Germany), with the recommended universal thermal cycling parameters, was used for amplification. Each reaction was run in at least triplicate. 18S rRNA was used as a reference transcript for normalization. Quality of qRT-PCR was were assessed by melting curve analysis for primers and amplicons. Expression levels were determined by the 2^−ΔΔCT^ method, where ΔΔC_T_ = (C_T[gene-infected]_−C_T[18S-infected]_)−(C_T[gene-uninfected]_−C_T[18S-uninfected]_).

### Data Deposition

Minimum Information About a Microarray Experiment (MIAME) data from this experiment has been deposited to the NCBI’s Gene Expression Omnibus (GEO) database. Microarray data can be accessed with the following accession numbers: GEO platform number GPL3213, GEO series number GSE39602 and sample accession numbers GSM972785–GSM972791.

## Supporting Information

Figure S1 **Distribution of genes against log2(fold changes) and** ***q*** **-values.** (**A**) Plot of all probe sets against log2(fold change). The light grey area defines genes below 2-fold changes, while the dark grey area defines those below 3-fold changes. (**B**) Plot of log2(fold change) against *q*-values of all probe sets. Green lines define the boundaries of *q*-value at 0.05 and fold changes at 2X and 3X as indicated.
(TIF)

Figure S2 **Diagram of the top 20 mapped KEGG pathways with significantly regulated genes in cecal epithelia upon** ***Eimeria tenella*** **infection (panels A to T).** Regulated genes are colored by fold changes as follows: red, >17 folds; brown/orange, 17 to 9 folds; yellow, 8 to 2 folds; cyan, −2 to −4 folds; green, −4 to −8 folds; blue, −9 folds or larger.
(PDF)

Table S1 **Complete list of probe sets with** ***q*** **-values <5% in fold changes.**
(XLSX)

Table S2 **Complete list of regulated genes that could be mapped by gene ontology (GO), including GO terms,** ***p*** **-values, FDR (** ***q*** **) values and mappable genes.**
(XLSX)

Table S3 **List of KEGG pathways and associated genes.**
(PDF)

Table S4 **List of differentially expressed genes that could be manually assigned into major functional groups.** Up-regulated genes are in red, while down-regulated genes are in blue font.
(PDF)

Table S5 **List of primers used in real-time qRT-PCR analysis.**
(PDF)
