# Supplementary figures and images for: Transcriptome Analysis in Chicken Cecal Epithelia upon Infection by Eimeria tenella In Vivo
Source: PLoS One. 2013 May 30;8(5):e64236. doi: 10.1371/journal.pone.0064236 (PMC3667848; doi:10.1371/journal.pone.0064236)

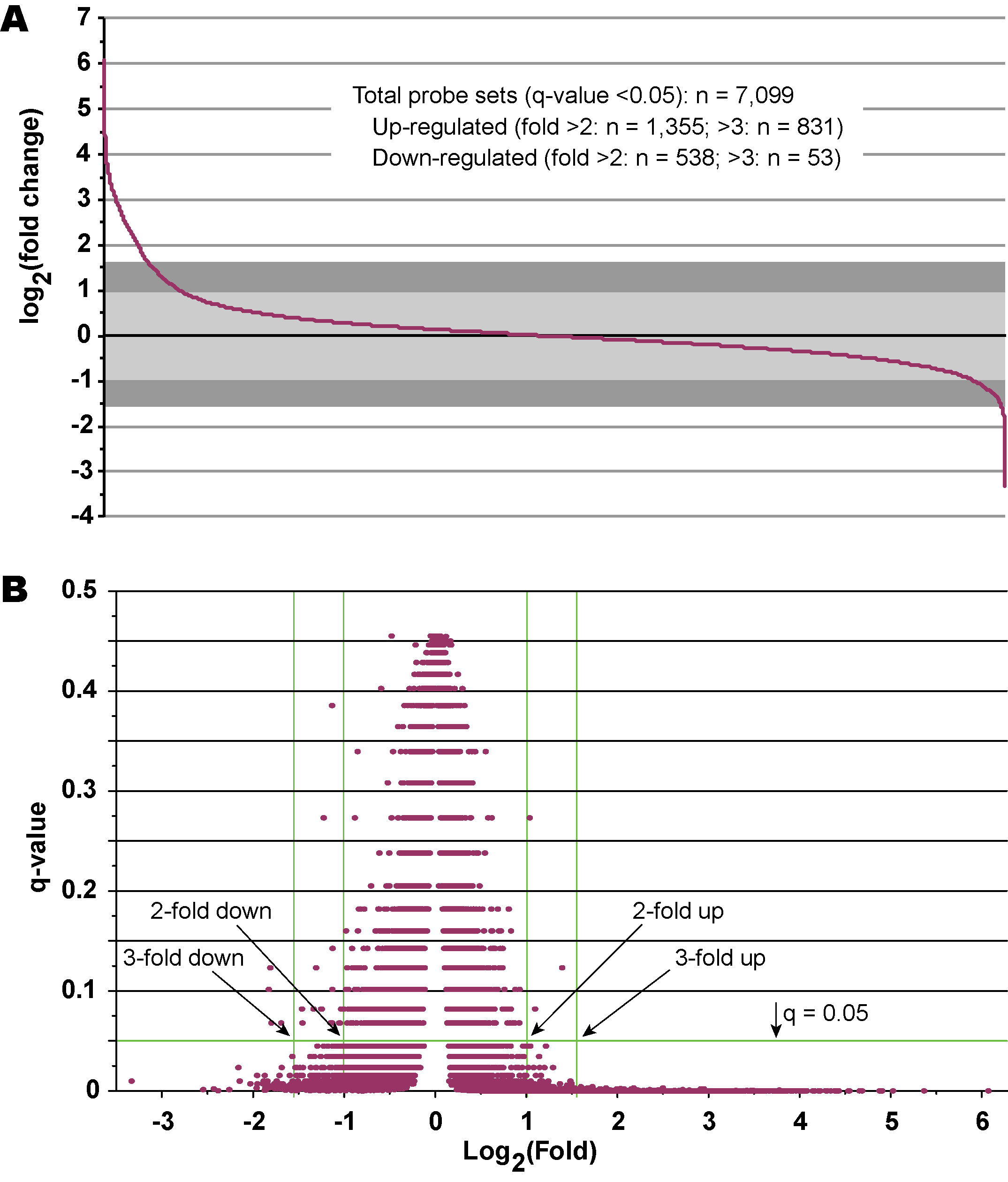

Supplement: Figure S1 — Distribution of genes against log2(fold changes) and q -values. (A) Plot of all probe sets against log2(fold change). The light grey area defines genes below 2-fold changes, while the dark grey area defines those below 3-fold changes. (B) Plot of log2(fold change) against q-values of all probe sets. Green lines define the boundaries of q-value at 0.05 and fold changes at 2X and 3X as indicated. (TIF) [file pone.0064236.s001.tif]
