# Supplementary material for: Transcriptome Analysis in Chicken Cecal Epithelia upon Infection by Eimeria tenella In Vivo
Source: PLoS One. 2013 May 30;8(5):e64236. doi: 10.1371/journal.pone.0064236 (PMC3667848; doi:10.1371/journal.pone.0064236)
Supplement: Figure S2 — Diagram of the top 20 mapped KEGG pathways with significantly regulated genes in cecal epithelia upon Eimeria tenella infection (panels A to T). Regulated genes are colored by fold changes as follows: red, >17 folds; brown/orange, 17 to 9 folds; yellow, 8 to 2 folds; cyan, −2 to −4 folds; green, −4 to −8 folds; blue, −9 folds or larger. (PDF) [file pone.0064236.s002.pdf]

Figure S2. Diagram of top 20 mapped KEGG pathways with significantly regulated genes in the cecal epithelia upon *Eimeria tenella* (Lanzhou-1 strain) infection (Panels A to T). Regulated genes are colored by fold changes as follows: red, >17 folds; brown/orange, 17 to 9 folds; yellow, 8 to 2 folds; cyan, -2 to -4 folds; green, -4 to -8 folds; blue, -9 folds or larger.

A

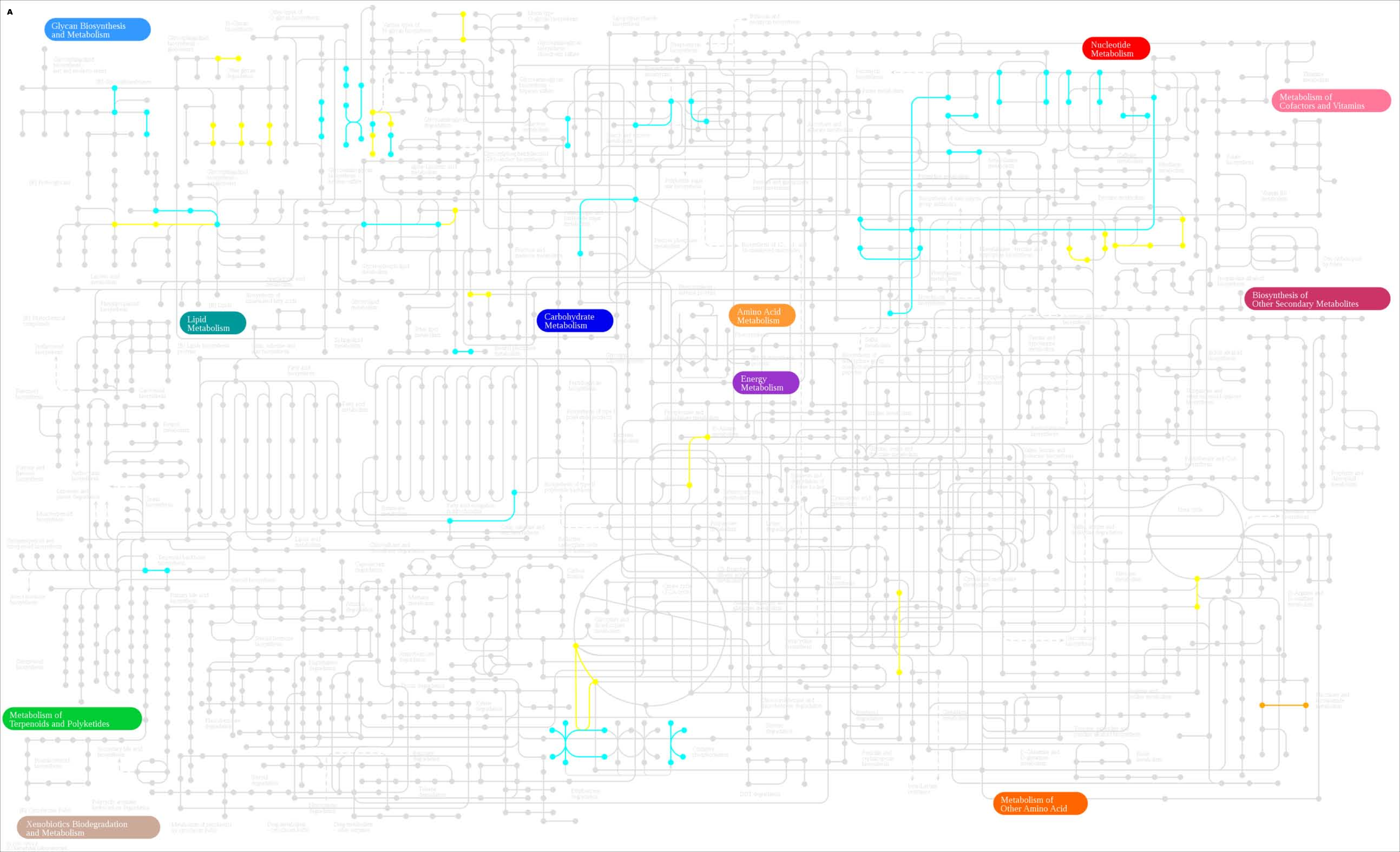
Glycan Biosynthesis and Metabolism
Nucleotide Metabolism
Metabolism of Cofactors and Vitamins
Biosynthesis of Other Secondary Metabolites
Lipid Metabolism
Carbohydrate Metabolism
Amino Acid Metabolism
Energy Metabolism
Metabolism of Terpenoids and Polyketides
Metabolism of Other Amino Acid
Xenobiotics Biodegradation and Metabolism

B

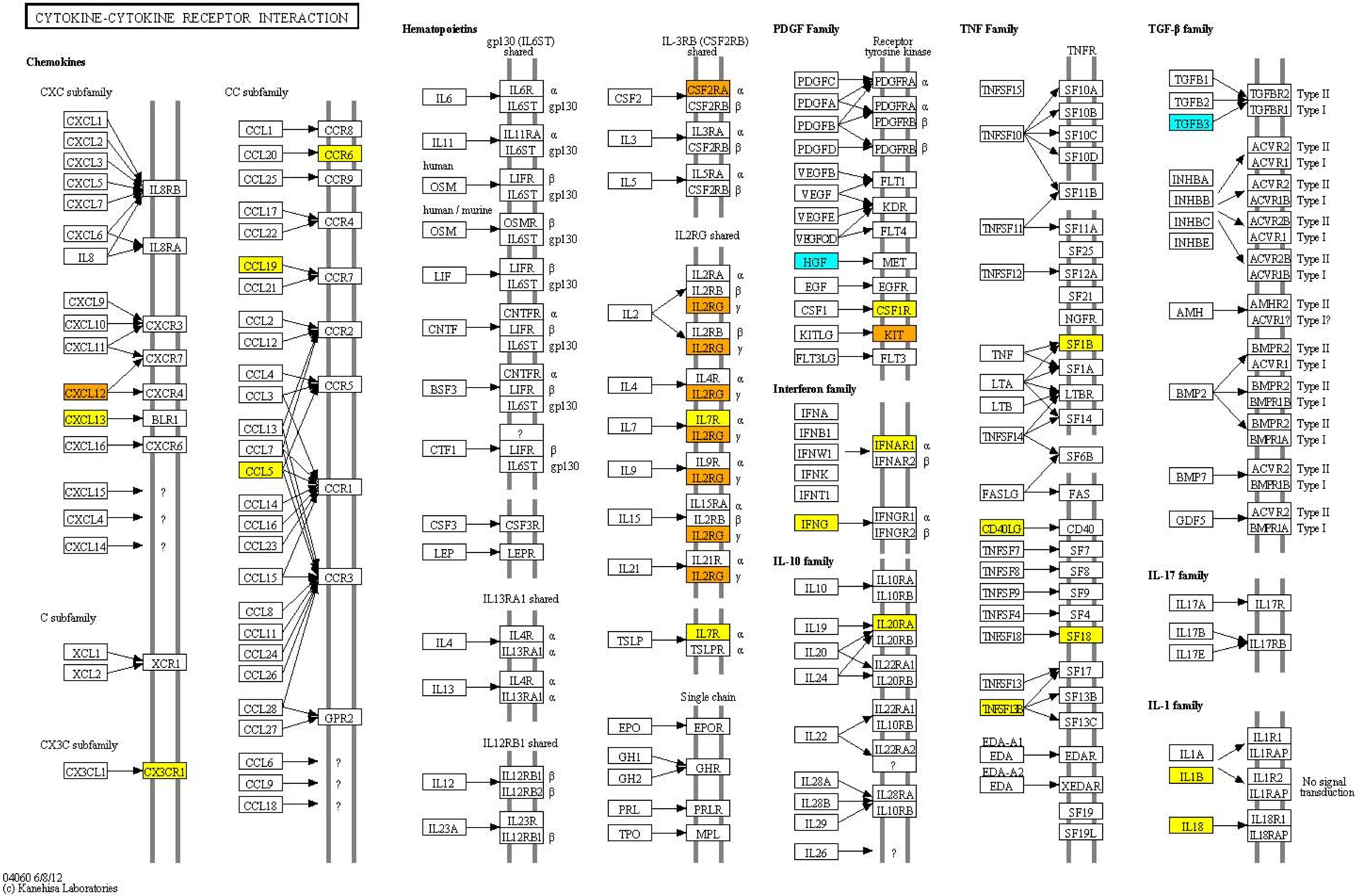

CYTOKINE-CYTOKINE RECEPTOR INTERACTION
Chemokines
CXC subfamily
CXCL1
CXCL2
CXCL3
CXCL5
CXCL7
CXCL6
IL8
IL8RB
IL8RA
CXCL9
CXCL10
CXCL11
CXCR3
CXCR7
CXCL12
CXCR4
CXCL13
BLR1
CXCL16
CXCR6
CXCL15
CXCL4
CXCL14
?
C subfamily
XCL1
XCL2
XCR1
CX3C subfamily
CX3CL1
CX3CR1
CC subfamily
CCL1
CCR8
CCL20
CCR6
CCL25
CCR9
CCL17
CCL22
CCR4
CCL19
CCL21
CCR7
CCL2
CCL12
CCR2
CCL4
CCL3
CCR5
CCL13
CCL7
CCL5
CCR1
CCL14
CCL16
CCL23
CCL15
CCR3
CCL8
CCL11
CCL24
CCL26
CCL28
CCL27
GPR2
CCL6
CCL9
CCL18
Hematopoietins
gp130 (IL6ST) shared
IL6
IL6R α
IL6ST gp130
IL11
IL11RA α
human
OSM
LIFR β
human / murine
OSMR β
LIF
CNTF
CNTFR α
BSF3
CTF1
?
CSF3
CSF3R
LEP
LEPR
IL13RA1 shared
IL4
IL4R α
IL13RA1 α
IL13
IL12RB1 shared
IL12
IL12RB1 β
IL12RB2 β
IL23A
IL23R
IL-3RB (CSF2RB) shared
CSF2
CSF2RA α
CSF2RB β
IL3
IL3RA α
IL5
IL5RA α
IL2RG shared
IL2
IL2RA α
IL2RB β
IL2RG γ
IL4R α
IL7
IL7R α
IL9
IL9R α
IL15
IL15RA α
IL21
IL21R α
TSLP
TSLPR α
Single chain
EPO
EPOR
GH1
GH2
GHR
PRL
PRLR
TPO
MPL
PDGF Family
Receptor tyrosine kinase
PDGFC
PDGFA
PDGFB
PDGFD
PDGFRA α
PDGFRB β
VEGFB
VEGF
VEGFE
VEGFD
FLT1
KDR
FLT4
HGF
MET
EGF
EGFR
CSF1
CSF1R
KITLG
KIT
FLT3LG
FLT3
Interferon family
IFNA
IFNB1
IFNW1
IFNK
IFNT1
IFNAR1 α
IFNAR2 β
IFNG
IFNGR1 α
IFNGR2 β
IL-10 family
IL10
IL10RA
IL10RB
IL19
IL20
IL24
IL20RA
IL20RB
IL22RA1
IL22
IL22RA2
IL28A
IL28B
IL29
IL28RA
IL26
TNF Family
TNFR
TNFSF15
TNFSF10
SF10A
SF10B
SF10C
SF10D
SF11B
TNFSF11
SF11A
SF25
TNFSF12
SF12A
SF21
NGFR
TNF
LTA
LTB
TNFSF14
SF1B
SF1A
LTBR
SF14
SF6B
FASLG
FAS
CD40LG
CD40
TNFSF7
SF7
TNFSF8
SF8
TNFSF9
SF9
TNFSF4
SF4
TNFSF18
SF18
TNFSF13
TNFSF13B
SF17
SF13B
SF13C
EDA-A1
EDA
EDAR
EDA-A2
XEDAR
SF19
SF19L
TGF-β family
TGFB1
TGFB2
TGFB3
TGFBR2 Type II
TGFBR1 Type I
INHBA
INHBB
INHBC
INHBE
ACVR2 Type II
ACVR1 Type I
ACVR1B Type I
ACVR2B Type II
AMH
AMHR2 Type II
ACVR1? Type I?
BMP2
BMPR2 Type II
BMPR1B Type I
BMPR1A Type I
BMP7
GDF5
IL-17 family
IL17A
IL17R
IL17B
IL17E
IL17RB
IL-1 family
IL1A
IL1R1
IL1RAP
IL1B
IL1R2
No signal transduction
IL18
IL18R1
IL18RAP
04060 6/8/12
(c) Kanehisa Laboratories

c

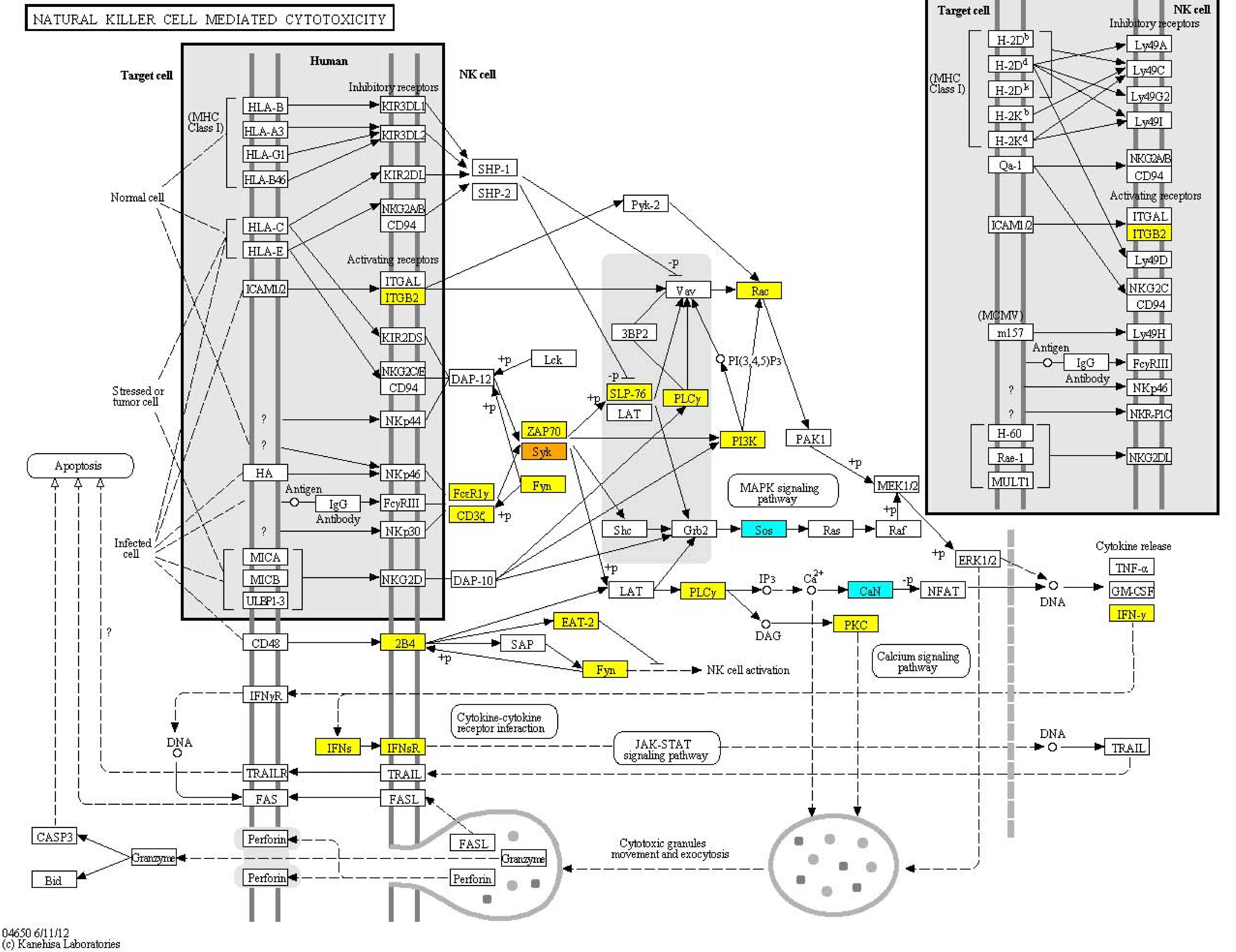

NATURAL KILLER CELL MEDIATED CYTOTOXICITY
Human
Target cell
NK cell
Inhibitory receptors
(MHC Class I)
HLA-B
HLA-A3
HLA-G1
HLA-B46
KIR3DL1
KIR3DL2
KIR2DL
NKG2A/B
CD94
Normal cell
HLA-C
HLA-E
Activating receptors
ICAM1/2
ITGAL
ITGB2
KIR2DS
NKG2C/E
CD94
Stressed or tumor cell
NKp44
HA
NKp46
Antigen
IgG
Antibody
FcγRIII
NKp30
Infected cell
MICA
MICB
ULBP1-3
NKG2D
DAP-10
DAP-12
CD48
2B4
SAP
EAT-2
Fyn
NK cell activation
SHP-1
SHP-2
Pyk-2
Lck
ZAP70
Syk
Fyn
FcεR1γ
CD3ζ
Vav
Rac
3BP2
SLP-76
LAT
PLCγ
PI(3,4,5)P3
PI3K
PAK1
MEK1/2
MAPK signaling pathway
Shc
Grb2
Sos
Ras
Raf
ERK1/2
LAT
PLCγ
IP3
Ca2+
CaN
NFAT
DAG
PKC
DNA
Cytokine release
TNF-α
GM-CSF
IFN-γ
Calcium signaling pathway
Apoptosis
IFNγR
DNA
IFNs
IFNsR
Cytokine-cytokine receptor interaction
JAK-STAT signaling pathway
DNA
TRAIL
TRAILR
TRAIL
FAS
FASL
FASL
CASP3
Granzyme
Bid
Perforin
Perforin
Granzyme
Perforin
Cytotoxic granules movement and exocytosis
+p
-p
?
Mouse
Target cell
NK cell
Inhibitory receptors
(MHC Class I)
H-2Db
H-2Dd
H-2Dk
H-2Kb
H-2Kd
Ly49A
Ly49C
Ly49G2
Ly49I
Qa-1
NKG2A/B
CD94
Activating receptors
ICAM1/2
ITGAL
ITGB2
Ly49D
NKG2C
CD94
(MCMV)
m157
Ly49H
Antigen
IgG
Antibody
FcγRIII
NKp46
NKR-P1C
H-60
Rae-1
MULT1
NKG2D
04650 6/11/12
(c) Kanehisa Laboratories

D

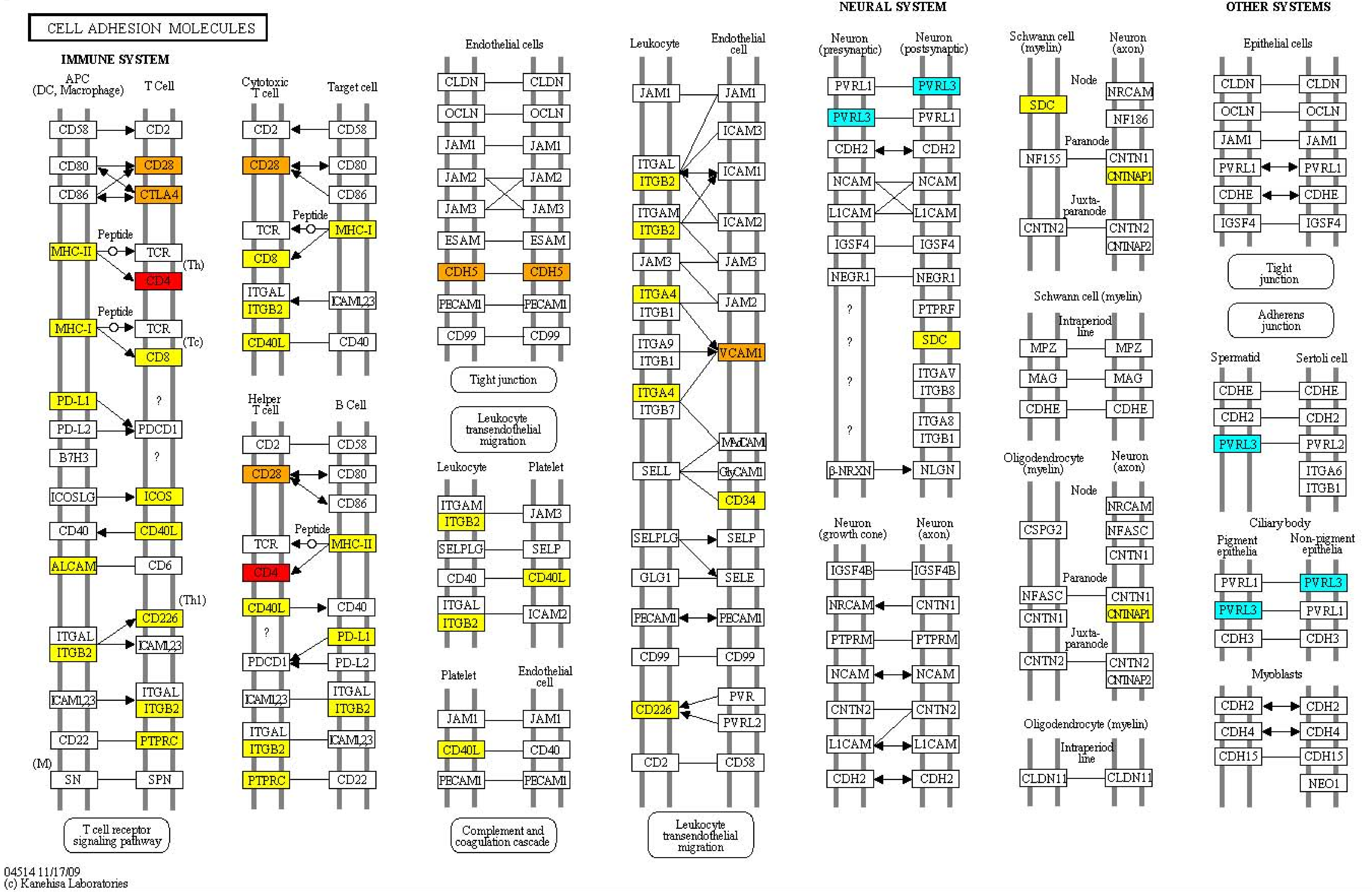

CELL ADHESION MOLECULES
IMMUNE SYSTEM
APC (DC, Macrophage)
T Cell
CD58
CD2
CD80
CD28
CD86
CTLA4
Peptide
MHC-II
TCR
(Th)
CD4
MHC-I
(Tc)
CD8
PD-L1
?
PDCD1
PD-L2
B7H3
ICOSLG
ICOS
CD40
CD40L
ALCAM
CD6
(Th1)
CD226
ITGAL
ITGB2
ICAM1,2,3
PTPRC
CD22
(M)
SN
SPN
T cell receptor signaling pathway
Cytotoxic T cell
Target cell
Helper T cell
B Cell
Endothelial cells
CLDN
OCLN
JAM1
JAM2
JAM3
ESAM
CDH5
PECAM1
CD99
Tight junction
Leukocyte transendothelial migration
Leukocyte
Platelet
ITGAM
SELPLG
SELP
ICAM2
Endothelial cell
Complement and coagulation cascade
ICAM3
ICAM1
ITGA4
ITGB1
ITGA9
VCAM1
ITGB7
MAdCAM1
SELL
GlyCAM1
CD34
SELE
GLG1
PVR
PVRL2
NEURAL SYSTEM
Neuron (presynaptic)
Neuron (postsynaptic)
PVRL1
PVRL3
CDH2
NCAM
L1CAM
IGSF4
NEGR1
PTPRF
SDC
ITGAV
ITGB8
ITGA8
β-NRXN
NLGN
Neuron (growth cone)
Neuron (axon)
IGSF4B
NRCAM
CNTN1
PTPRM
CNTN2
Schwann cell (myelin)
Node
NF186
Paranode
NF155
CNTNAP1
Juxta-paranode
CNTNAP2
Intraperiod line
MPZ
MAG
CDHE
Oligodendrocyte (myelin)
CSPG2
NFASC
CLDN11
OTHER SYSTEMS
Epithelial cells
Adherens junction
Spermatid
Sertoli cell
ITGA6
Ciliary body
Pigment epithelia
Non-pigment epithelia
CDH3
Myoblasts
CDH4
CDH15
NEO1
04514 11/17/09
(c) Kanehisa Laboratories

E

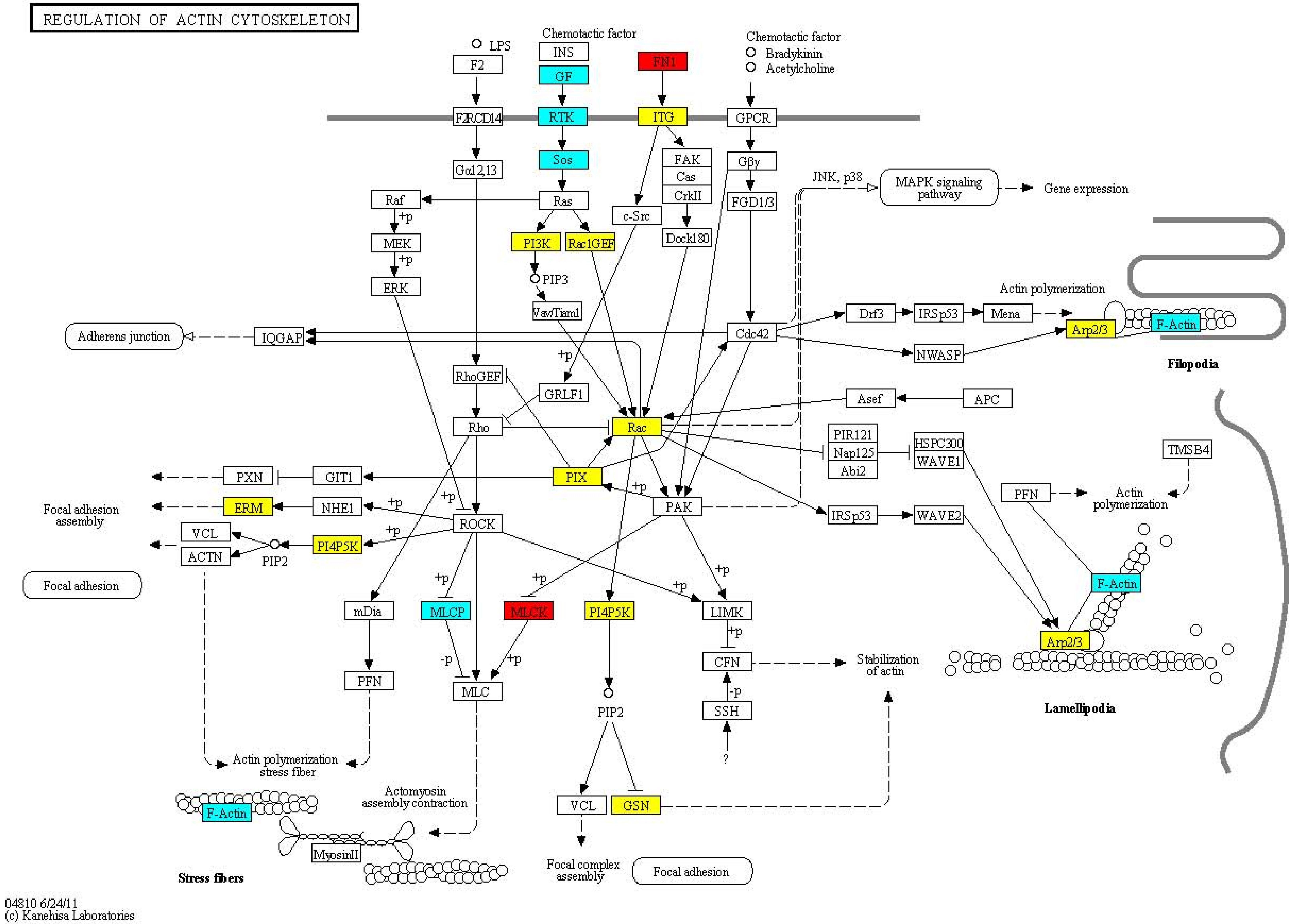

REGULATION OF ACTIN CYTOSKELETON
LPS
F2
Chemotactic factor
INS
GF
FN1
Chemotactic factor
Bradykinin
Acetylcholine
F2R CD14
RTK
ITG
GPCR
Sos
FAK
Cas
CrkII
Gβγ
Gα12,13
Raf
Ras
c-Src
Dock180
FGD1/3
JNK, p38
MAPK signaling pathway
Gene expression
MEK
PI3K
Rac1GEF
ERK
PIP3
Vav/Tiam1
Actin polymerization
Drf3
IRSp53
Mena
Arp2/3
F-Actin
Adherens junction
IQGAP
Cdc42
NWASP
Filopodia
RhoGEF
GRLF1
Asef
APC
Rho
Rac
PIR121
Nap125
Abi2
HSPC300
WAVE1
TMSB4
PXN
GIT1
PIX
Focal adhesion assembly
ERM
NHE1
ROCK
PAK
IRSp53
WAVE2
PFN
Actin polymerization
VCL
ACTN
PIP2
PI4P5K
F-Actin
Focal adhesion
mDia
MLCP
MLCK
PI4P5K
LIMK
CFN
Stabilization of actin
Arp2/3
PFN
MLC
SSH
Lamellipodia
PIP2
?
Actin polymerization stress fiber
Actomyosin assembly contraction
F-Actin
VCL
GSN
MyosinII
Stress fibers
Focal complex assembly
Focal adhesion
+p
-p
04810 6/24/11
(c) Kanehisa Laboratories

F

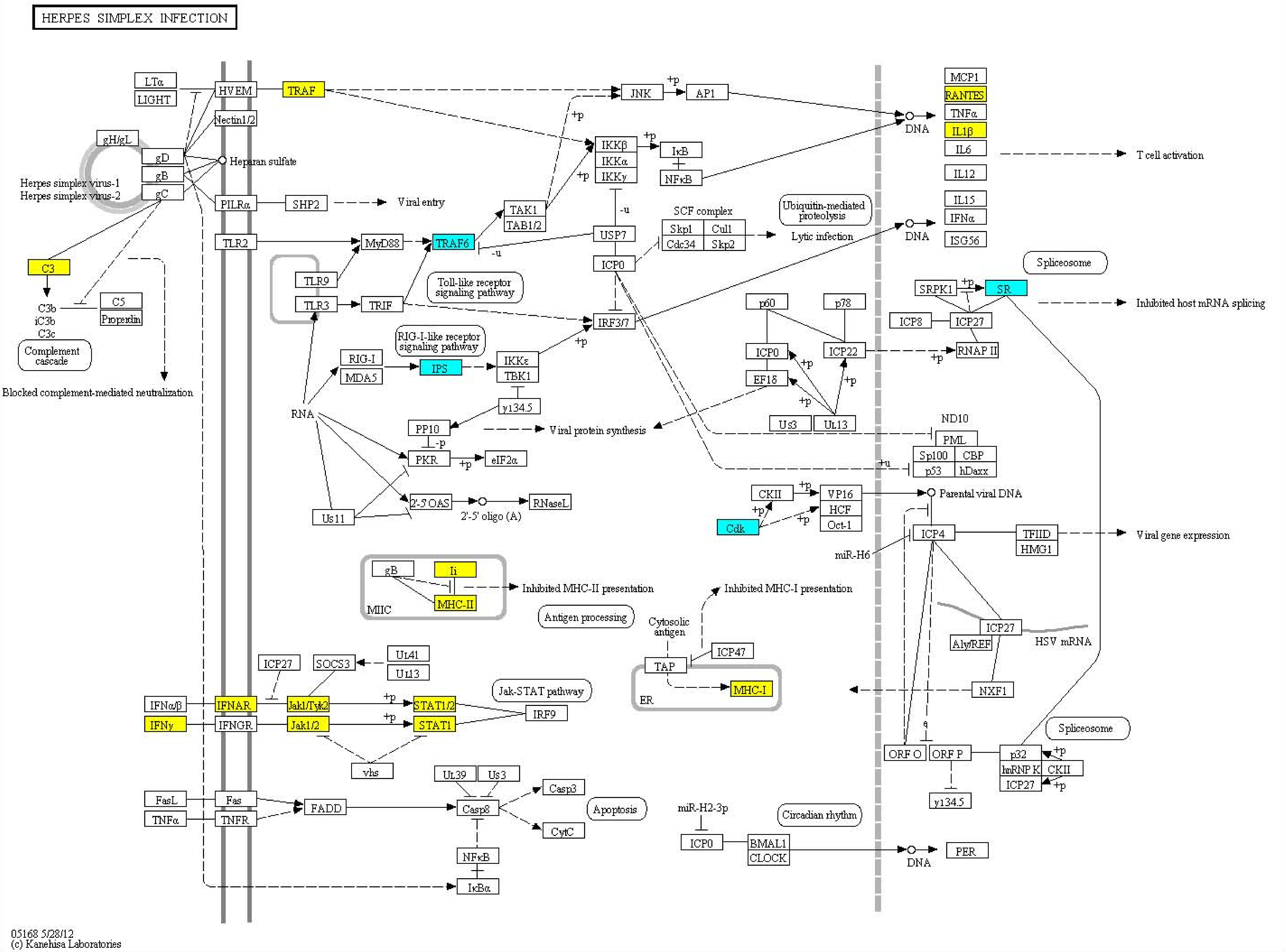
HERPES SIMPLEX INFECTION
LTα
LIGHT
HVEM
TRAF
Nectin1/2
gH/gL
gD
gB
gC
Heparan sulfate
Herpes simplex virus-1
Herpes simplex virus-2
PILRα
SHP2
Viral entry
TLR2
MyD88
TRAF6
TAK1
TAB1/2
JNK
AP1
IKKβ
IKKα
IKKγ
IκB
NFκB
DNA
MCP1
RANTES
TNFα
IL1β
IL6
IL12
IL15
IFNα
ISG56
T cell activation
USP7
ICP0
SCF complex
Skp1
Cul1
Cdc34
Skp2
Ubiquitin-mediated proteolysis
Lytic infection
C3
C3b
iC3b
C3c
C5
Properdin
Complement cascade
Blocked complement-mediated neutralization
TLR9
TLR3
TRIF
Toll-like receptor signaling pathway
IRF3/7
Spliceosome
SRPK1
SR
ICP8
ICP27
Inhibited host mRNA splicing
p60
p78
ICP0
ICP22
RNAP II
EF1δ
RIG-I
MDA5
IPS
RIG-I-like receptor signaling pathway
IKKε
TBK1
γ134.5
RNA
PP10
Viral protein synthesis
Us3
UL13
ND10
PML
Sp100
CBP
p53
hDaxx
PKR
eIF2α
2'-5'OAS
2'-5' oligo (A)
RNaseL
Us11
CKII
VP16
HCF
Oct-1
Cdk
Parental viral DNA
ICP4
TFIID
HMG1
miR-H6
Viral gene expression
gB
Ii
MHC-II
MIIC
Inhibited MHC-II presentation
Antigen processing
Inhibited MHC-I presentation
Cytosolic antigen
TAP
ICP47
MHC-I
ER
ICP27
Aly/REF
HSV mRNA
NXF1
ICP27
SOCS3
UL41
UL13
IFNα/β
IFNAR
Jak1/Tyk2
STAT1/2
Jak-STAT pathway
IRF9
IFNγ
IFNGR
Jak1/2
STAT1
vhs
ORF O
ORF P
γ134.5
p32
hnRNP K
CKII
ICP27
Spliceosome
UL39
Us3
FasL
Fas
TNFα
TNFR
FADD
Casp8
Casp3
CytC
Apoptosis
NFκB
IκBα
miR-H2-3p
ICP0
BMAL1
CLOCK
Circadian rhythm
DNA
PER
05168 5/28/12
(c) Kanehisa Laboratories

G

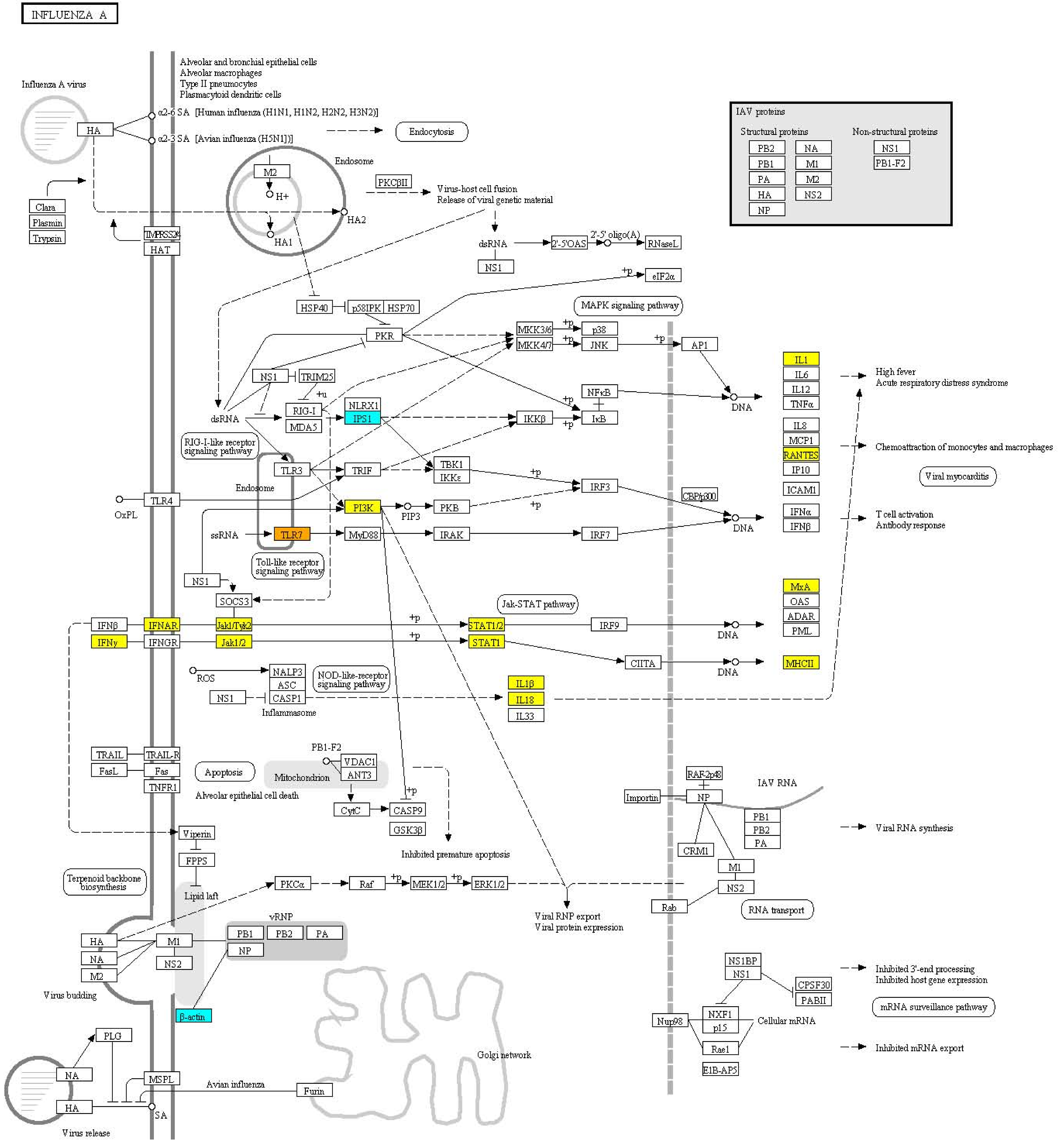

05164 6/3/11
(c) Kanehisa Laboratories

H

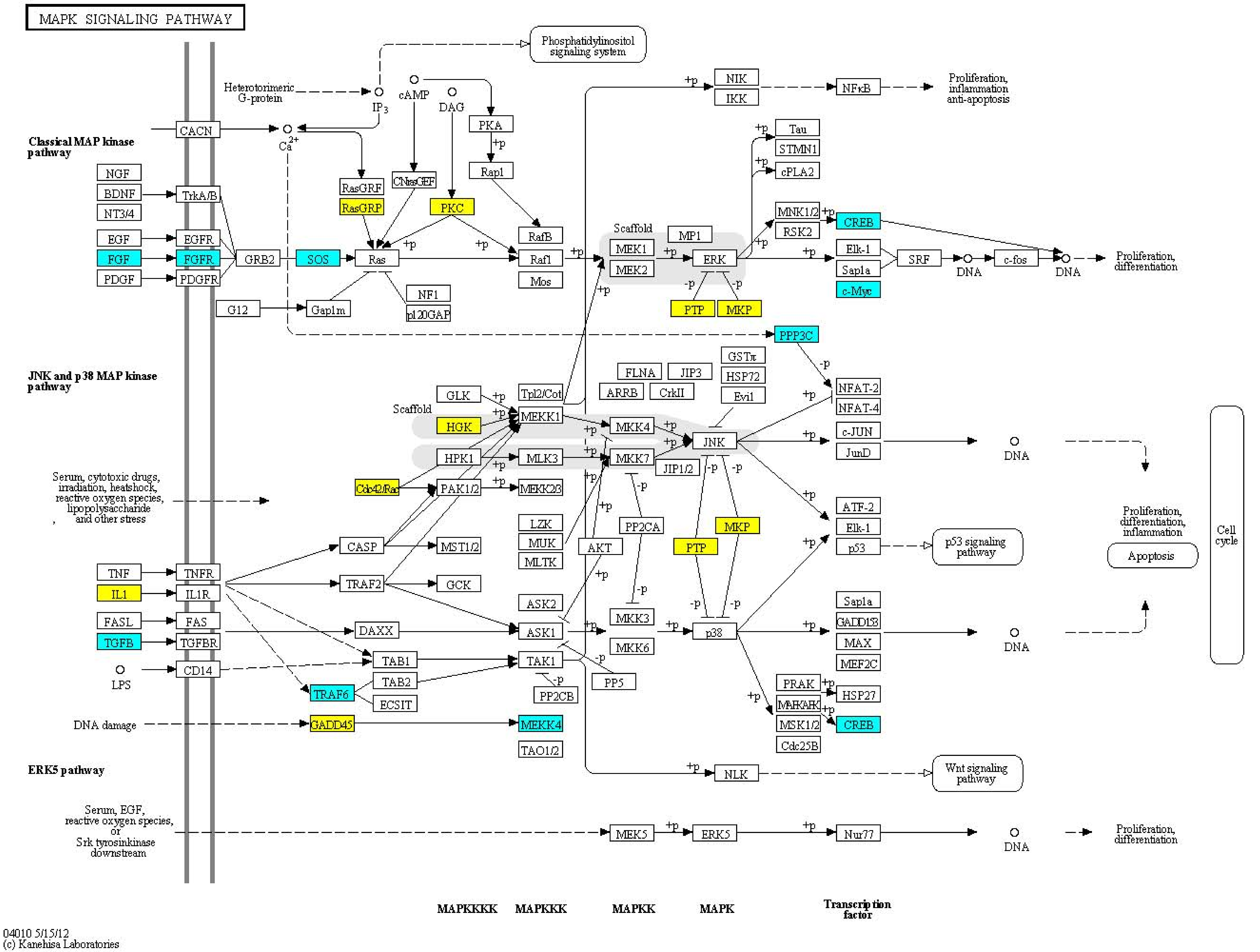
MAPK SIGNALING PATHWAY
Classical MAP kinase pathway
JNK and p38 MAP kinase pathway
ERK5 pathway
Phosphatidylinositol signaling system
Heterotorimeric G-protein
IP3
cAMP
DAG
CACN
Ca2+
PKA
Rap1
RasGRF
CNrasGEF
RasGRP
PKC
RafB
Raf1
Mos
NGF
BDNF
NT3/4
TrkA/B
EGF
EGFR
FGF
FGFR
PDGF
PDGFR
GRB2
SOS
Ras
NF1
p120GAP
G12
Gap1m
Scaffold
MP1
MEK1
MEK2
ERK
PTP
MKP
NIK
IKK
NFκB
Proliferation, inflammation anti-apoptosis
Tau
STMN1
cPLA2
MNK1/2
RSK2
CREB
Elk-1
Sap1a
c-Myc
SRF
DNA
c-fos
Proliferation, differentiation
PPP3C
NFAT-2
NFAT-4
GSTπ
HSP72
Evi1
FLNA
JIP3
ARRB
CrkII
GLK
Tpl2/Cot
HGK
MEKK1
HPK1
MLK3
MKK4
MKK7
JNK
JIP1/2
c-JUN
JunD
Cdc42/Rac
PAK1/2
MEKK2/3
ATF-2
p53
p53 signaling pathway
Serum, cytotoxic drugs, irradiation, heatshock, reactive oxygen species, lipopolysaccharide , and other stress
LZK
MUK
MLTK
PP2CA
AKT
Proliferation, differentiation, inflammation
Apoptosis
Cell cycle
TNF
TNFR
IL1
IL1R
FASL
FAS
TGFB
TGFBR
LPS
CD14
CASP
MST1/2
TRAF2
GCK
DAXX
ASK2
ASK1
MKK3
MKK6
p38
Sap1a
GADD153
MAX
MEF2C
TAB1
TAK1
TAB2
TRAF6
ECSIT
PP2CB
PP5
PRAK
MAPKAPK
MSK1/2
Cdc25B
HSP27
DNA damage
GADD45
MEKK4
TAO1/2
NLK
Wnt signaling pathway
Serum, EGF, reactive oxygen species, or Srk tyrosinkinase downstream
MEK5
ERK5
Nur77
+p
-p
MAPKKKK
MAPKKK
MAPKK
MAPK
Transcription factor
04010 5/15/12
(c) Kanehisa Laboratories

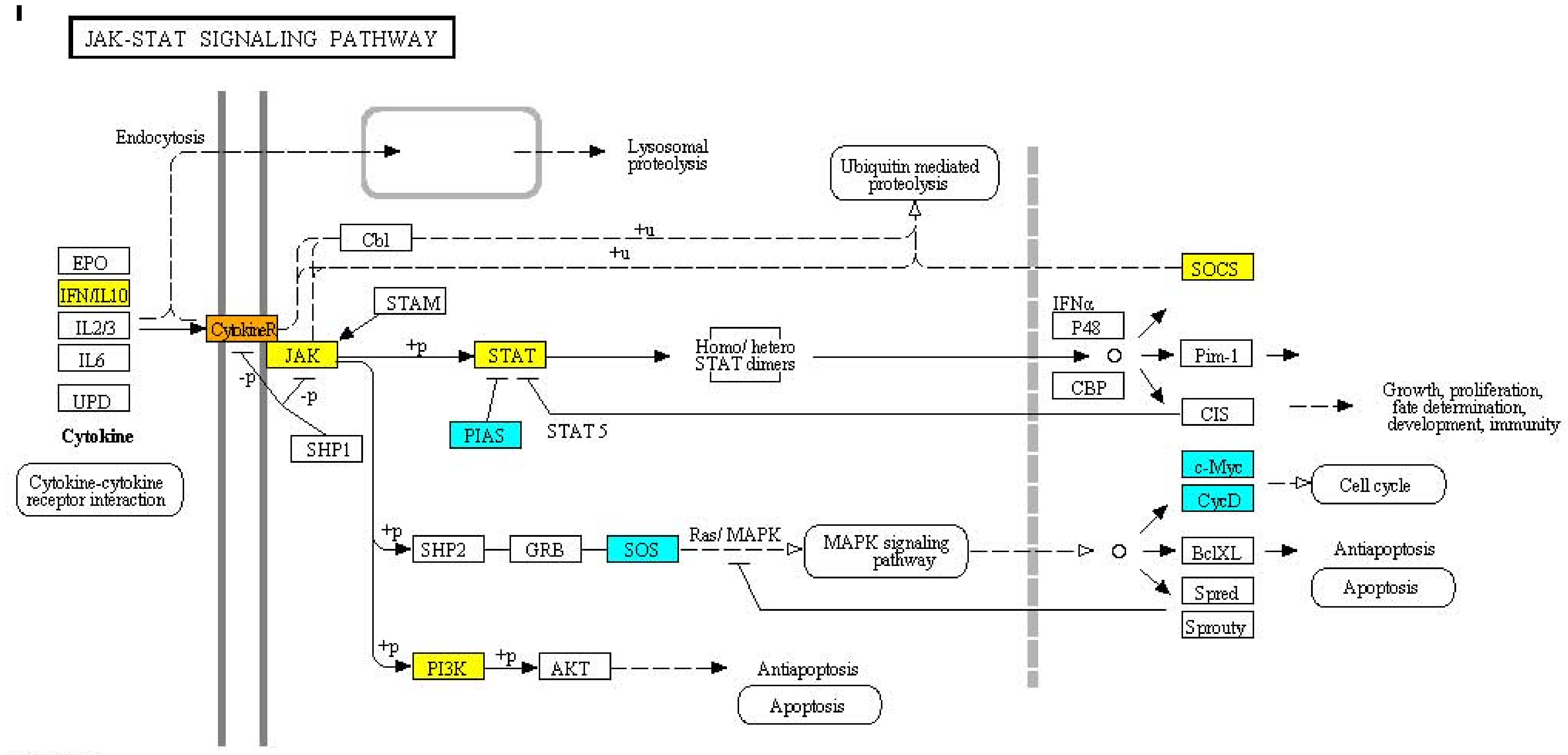

04630 6/8/12
(c) Kanehisa Laboratories

J

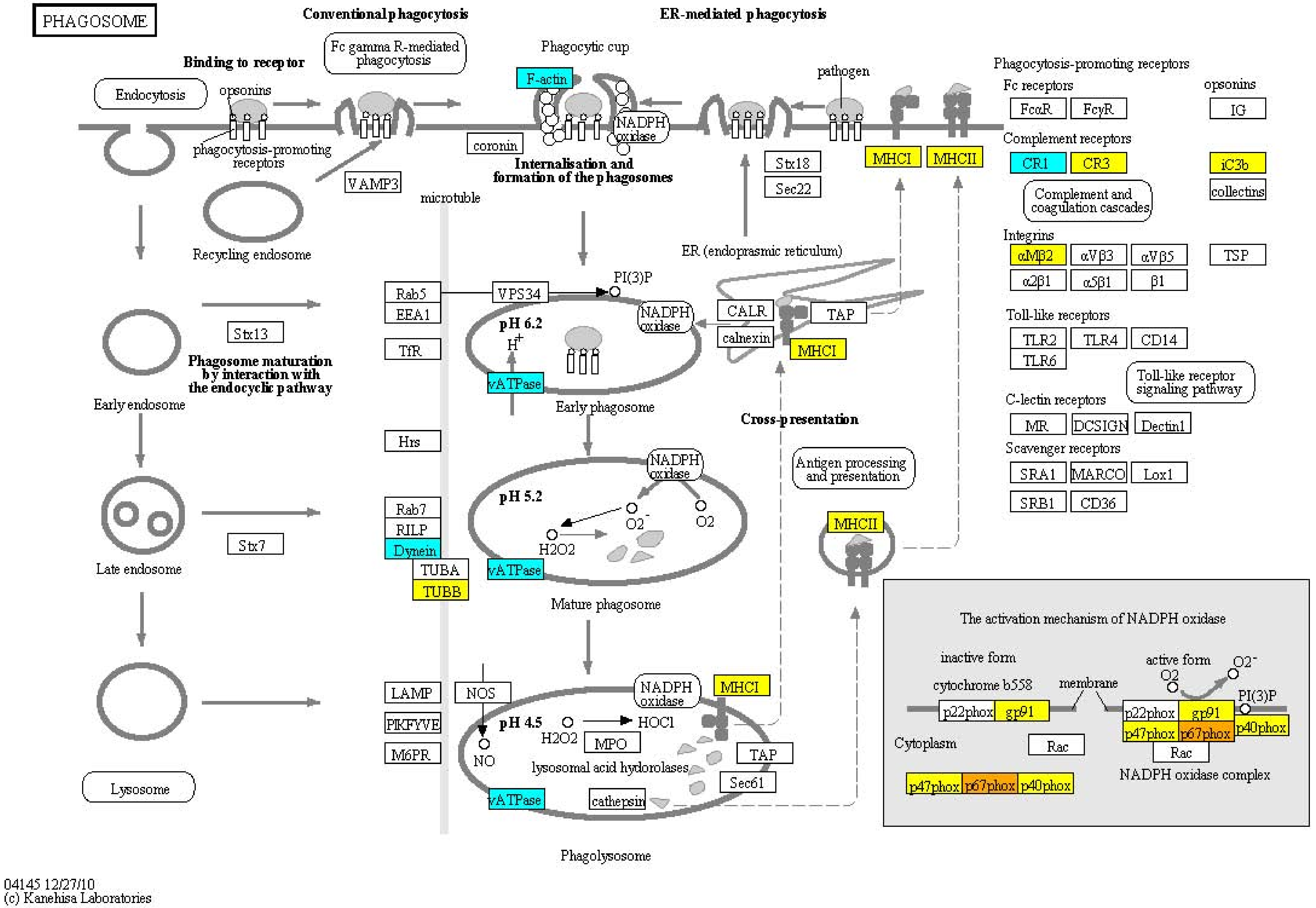

PHAGOSOME
Conventional phagocytosis
ER-mediated phagocytosis
Binding to receptor
Fc gamma R-mediated phagocytosis
Phagocytic cup
Endocytosis
opsonins
phagocytosis-promoting receptors
F-actin
NADPH oxidase
coronin
VAMP3
Recycling endosome
microtubule
Internalisation and formation of the phagosomes
pathogen
Stx18
Sec22
MHCI
MHCII
ER (endoprasmic reticulum)
Phagocytosis-promoting receptors
Fc receptors
FcαR
FcγR
opsonins
IG
Complement receptors
CR1
CR3
iC3b
Complement and coagulation cascades
collectins
Integrins
αMβ2
αVβ3
αVβ5
TSP
α2β1
α5β1
β1
Toll-like receptors
TLR2
TLR4
CD14
TLR6
Toll-like receptor signaling pathway
C-lectin receptors
MR
DCSIGN
Dectin1
Scavenger receptors
SRA1
MARCO
Lox1
SRB1
CD36
Early endosome
Stx13
Phagosome maturation by interaction with the endocyclic pathway
Rab5
EEA1
TfR
VPS34
PI(3)P
pH 6.2
H^+
vATPase
Early phagosome
CALR
calnexin
TAP
Cross-presentation
Antigen processing and presentation
Hrs
Rab7
RILP
Dynein
TUBA
TUBB
Late endosome
Stx7
pH 5.2
O_2^-
O2
H2O2
Mature phagosome
Lysosome
LAMP
PIKFYVE
M6PR
NOS
NO
pH 4.5
HOCl
MPO
lysosomal acid hydrolases
TAP
Sec61
cathepsin
Phagolysosome
The activation mechanism of NADPH oxidase
inactive form
cytochrome b558
membrane
p22phox
gp91
Cytoplasm
Rac
p47phox
p67phox
p40phox
active form
O2
O_2^-
PI(3)P
NADPH oxidase complex
04145 12/27/10
(c) Kanehisa Laboratories

K

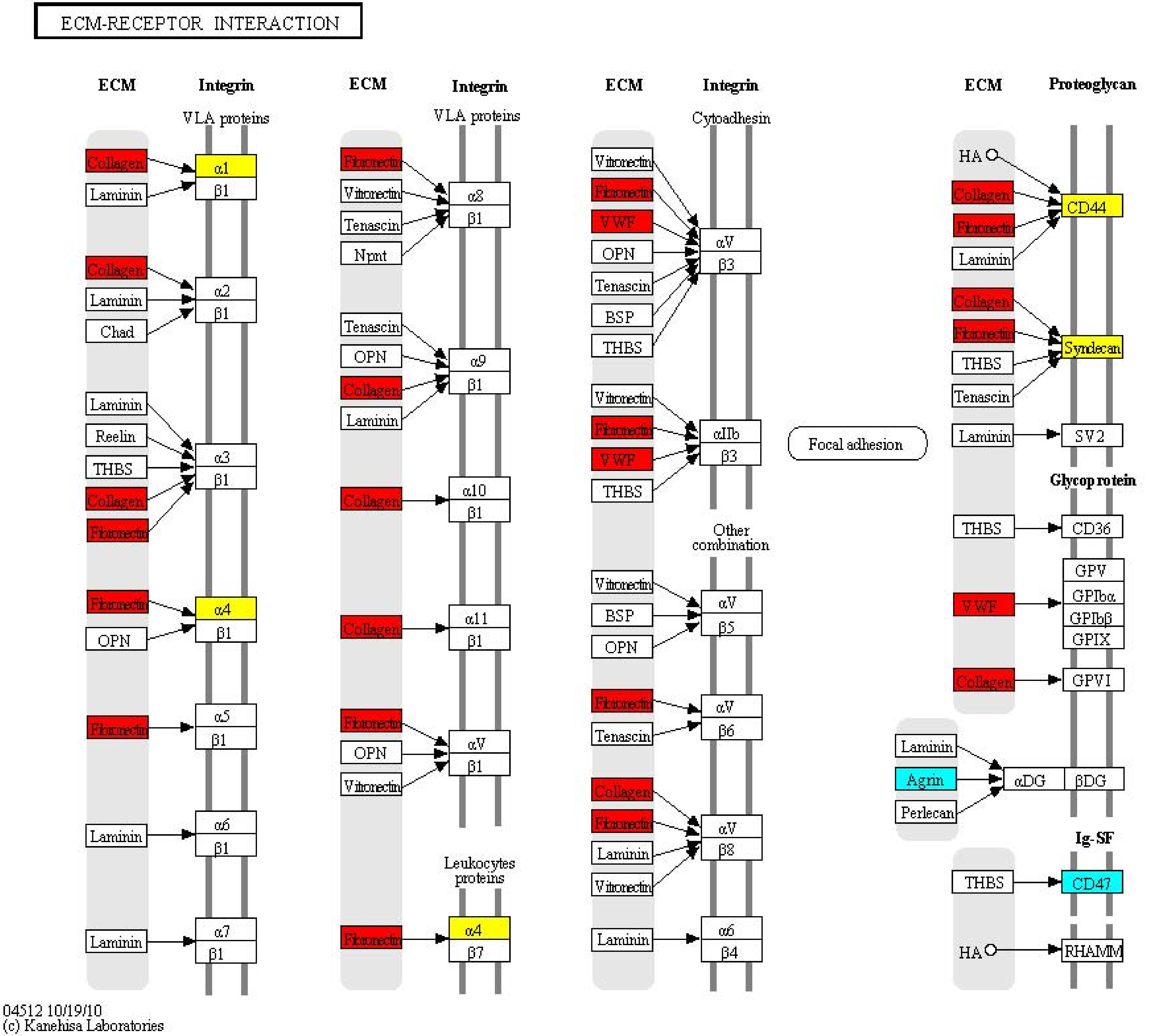
ECM-RECEPTOR INTERACTION
ECM
Integrin
VLA proteins
Collagen
Laminin
α1
β1
Collagen
Laminin
Chad
α2
β1
Laminin
Reelin
THBS
Collagen
Fibronectin
α3
β1
Fibronectin
OPN
α4
β1
Fibronectin
α5
β1
Laminin
α6
β1
Laminin
α7
β1
ECM
Integrin
VLA proteins
Fibronectin
Vitronectin
Tenascin
Npnt
α8
β1
Tenascin
OPN
Collagen
Laminin
α9
β1
Collagen
α10
β1
Collagen
α11
β1
Fibronectin
OPN
Vitronectin
αV
β1
Leukocytes proteins
Fibronectin
α4
β7
ECM
Integrin
Cytoadhesin
Vitronectin
Fibronectin
VWF
OPN
Tenascin
BSP
THBS
αV
β3
Vitronectin
Fibronectin
VWF
THBS
αIIb
β3
Other combination
Vitronectin
BSP
OPN
αV
β5
Fibronectin
Tenascin
αV
β6
Collagen
Fibronectin
Laminin
Vitronectin
αV
β8
Laminin
α6
β4
Focal adhesion
ECM
Proteoglycan
HA
Collagen
Fibronectin
Laminin
CD44
Collagen
Fibronectin
THBS
Tenascin
Syndecan
Laminin
SV2
Glycoprotein
THBS
CD36
VWF
GPV
GPIbα
GPIbβ
GPIX
Collagen
GPVI
Laminin
Agrin
Perlecan
αDG
βDG
Ig-SF
THBS
CD47
HA
RHAMM
04512 10/19/10
(c) Kanehisa Laboratories

L

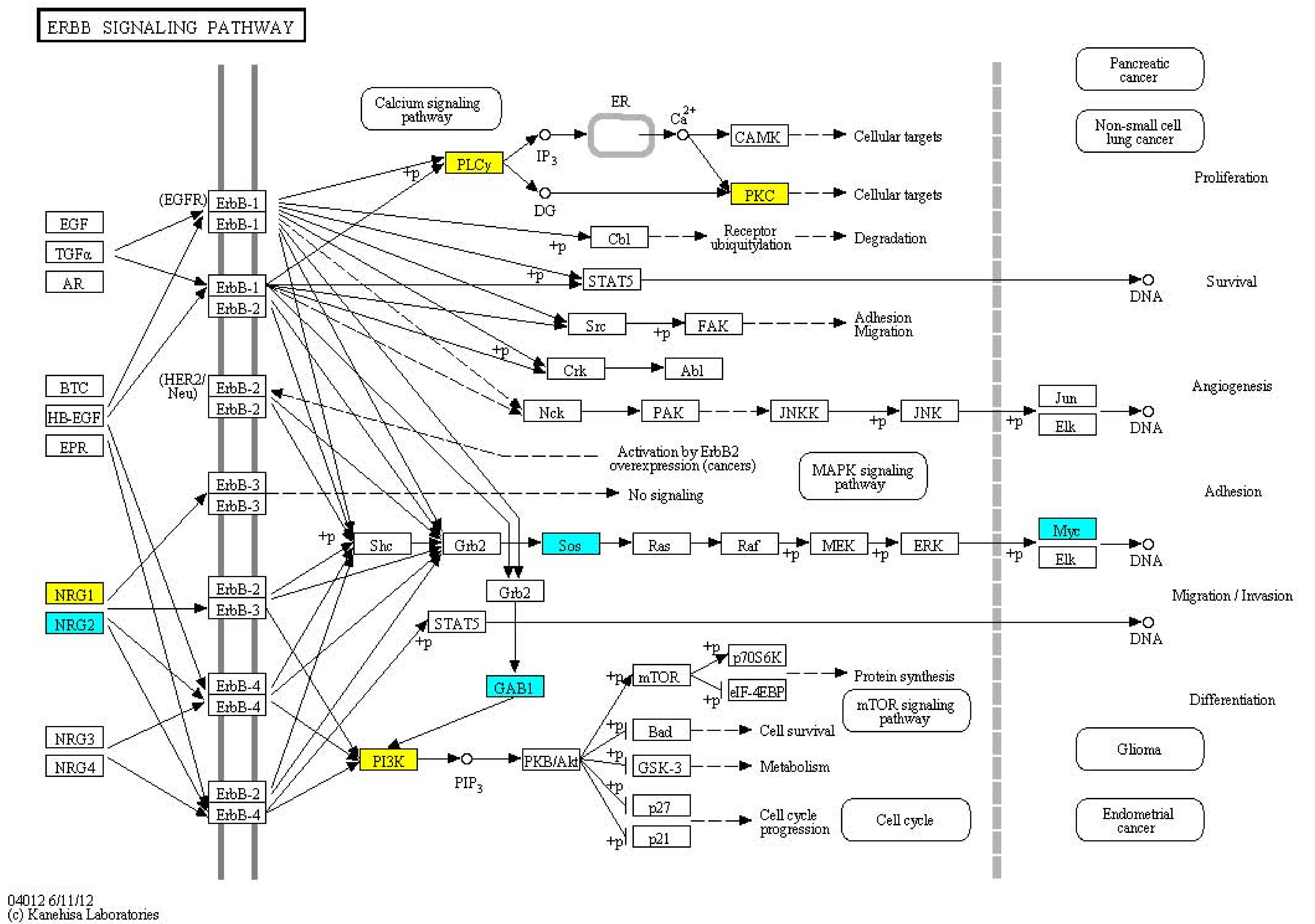

ERBB SIGNALING PATHWAY
Calcium signaling pathway
ER
Ca^{2+}
CAMK
Cellular targets
PLCγ
IP_3
DG
PKC
Cellular targets
Pancreatic cancer
Non-small cell lung cancer
Proliferation
(EGFR)
ErbB-1
ErbB-1
EGF
TGFα
AR
Cbl
Receptor ubiquitylation
Degradation
+p
STAT5
DNA
Survival
ErbB-1
ErbB-2
Src
FAK
Adhesion Migration
Crk
Abl
Angiogenesis
BTC
HB-EGF
EPR
(HER2/ Neu)
ErbB-2
ErbB-2
Nck
PAK
JNKK
JNK
Jun
Elk
DNA
Activation by ErbB2 overexpression (cancers)
MAPK signaling pathway
ErbB-3
ErbB-3
No signaling
Adhesion
Shc
Grb2
Sos
Ras
Raf
MEK
ERK
Myc
Elk
DNA
NRG1
NRG2
ErbB-2
ErbB-3
Grb2
Migration / Invasion
STAT5
DNA
GAB1
mTOR
p70S6K
eIF-4EBP
Protein synthesis
mTOR signaling pathway
Differentiation
ErbB-4
ErbB-4
NRG3
NRG4
Bad
Cell survival
Glioma
PI3K
PIP_3
PKB/Akt
GSK-3
Metabolism
ErbB-2
ErbB-4
p27
p21
Cell cycle progression
Cell cycle
Endometrial cancer
04012 6/11/12
(c) Kanehisa Laboratories

M

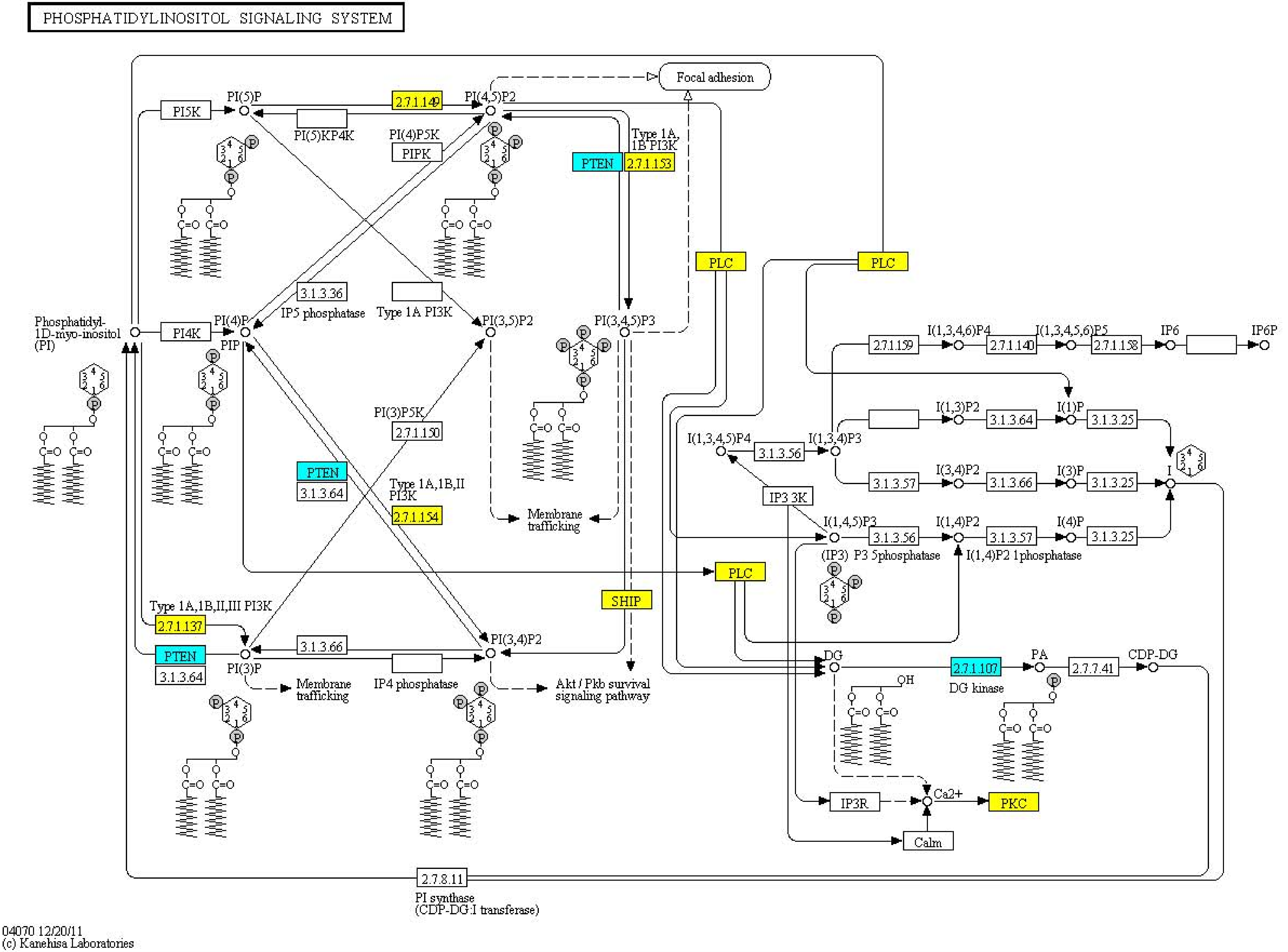
PHOSPHATIDYLINOSITOL SIGNALING SYSTEM
Focal adhesion
PI(5)P
PI5K
2.7.1.149
PI(4,5)P2
PI(5)KP4K
PI(4)P5K
PIPK
Type 1A, 1B PI3K
PTEN
2.7.1.153
PLC
PLC
3.1.3.36
IP5 phosphatase
Type 1A PI3K
Phosphatidyl-1D-myo-inositol (PI)
PI4K
PI(4)P
PIP
PI(3,5)P2
PI(3,4,5)P3
I(1,3,4,6)P4
I(1,3,4,5,6)P5
IP6
IP6P
2.7.1.159
2.7.1.140
2.7.1.158
PI(3)P5K
2.7.1.150
I(1,3)P2
I(1)P
3.1.3.64
3.1.3.25
I(1,3,4,5)P4
3.1.3.56
I(1,3,4)P3
PTEN
3.1.3.64
Type 1A,1B,II PI3K
2.7.1.154
I(3,4)P2
I(3)P
3.1.3.57
3.1.3.66
3.1.3.25
I
IP3 3K
Membrane trafficking
I(1,4,5)P3
I(1,4)P2
I(4)P
3.1.3.56
3.1.3.57
3.1.3.25
(IP3) P3 5phosphatase
I(1,4)P2 1phosphatase
PLC
SHIP
Type 1A,1B,II,III PI3K
2.7.1.137
3.1.3.66
PI(3,4)P2
PTEN
3.1.3.64
PI(3)P
IP4 phosphatase
DG
2.7.1.107
PA
2.7.7.41
CDP-DG
DG kinase
Membrane trafficking
Akt / Pkb survival signaling pathway
IP3R
Ca2+
PKC
Calm
2.7.8.11
PI synthase (CDP-DG:I transferase)
04070 12/20/11
(c) Kanehisa Laboratories

N

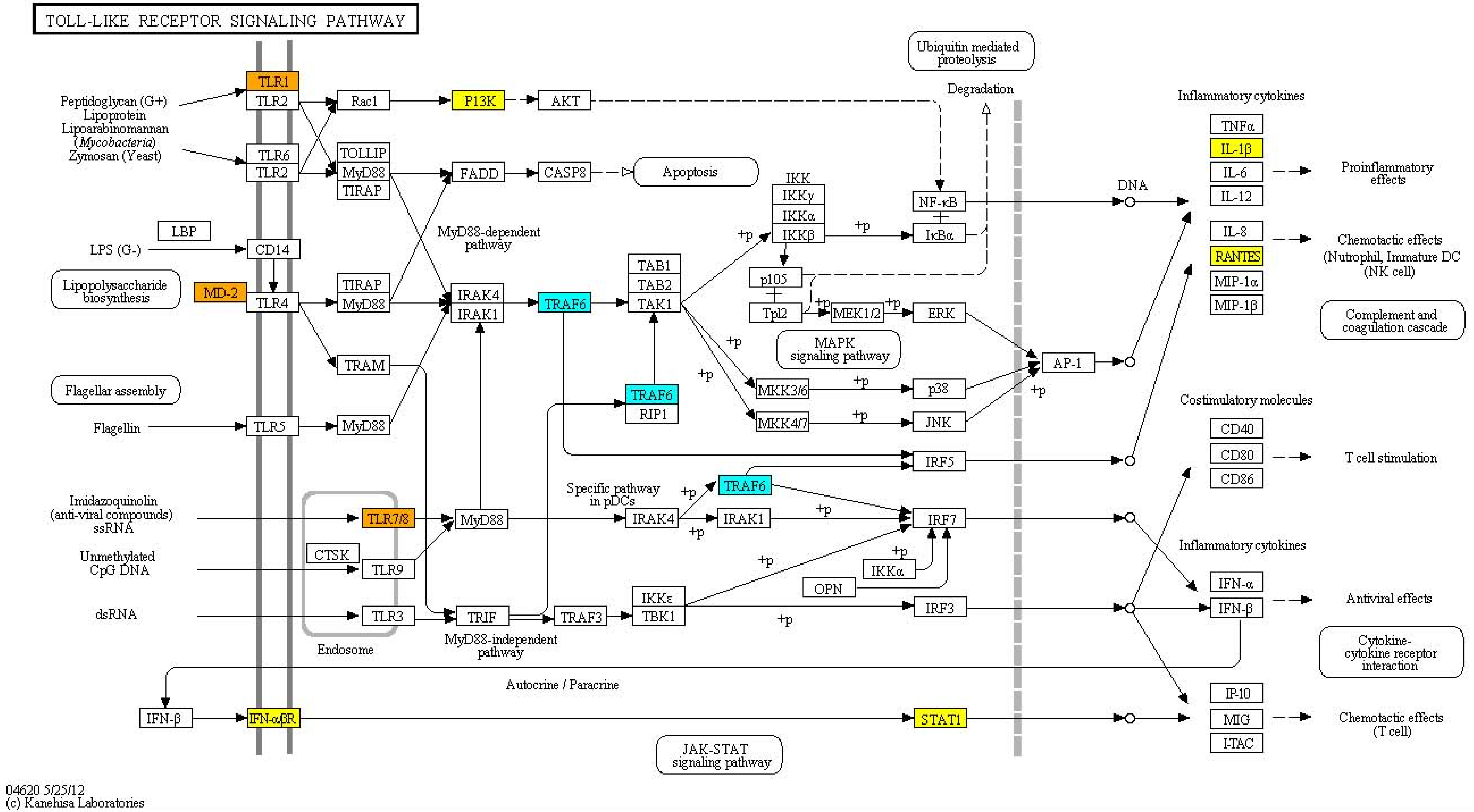

TOLL-LIKE RECEPTOR SIGNALING PATHWAY
Peptidoglycan (G+)
Lipoprotein
Lipoarabinomannan
(Mycobacteria)
Zymosan (Yeast)
TLR1
TLR2
TLR6
TLR2
LBP
LPS (G-)
CD14
Lipopolysaccharide biosynthesis
MD-2
TLR4
Flagellar assembly
Flagellin
TLR5
Rac1
PI3K
AKT
TOLLIP
MyD88
TIRAP
FADD
CASP8
Apoptosis
TIRAP
MyD88
TRAM
MyD88
MyD88-dependent pathway
IRAK4
IRAK1
TRAF6
TAB1
TAB2
TAK1
TRAF6
RIP1
IKK
IKKγ
IKKα
IKKβ
+p
p105
Tpl2
MEK1/2
ERK
MAPK signaling pathway
MKK3/6
p38
MKK4/7
JNK
AP-1
Ubiquitin mediated proteolysis
Degradation
NF-κB
IκBα
DNA
IRF5
Specific pathway in pDCs
TRAF6
IRAK4
IRAK1
IRF7
IKKα
OPN
Imidazoquinolin (anti-viral compounds) ssRNA
TLR7/8
MyD88
CTSK
Unmethylated CpG DNA
TLR9
dsRNA
TLR3
Endosome
TRIF
MyD88-independent pathway
TRAF3
IKKε
TBK1
IRF3
Autocrine / Paracrine
IFN-β
IFN-αβR
STAT1
JAK-STAT signaling pathway
Inflammatory cytokines
TNFα
IL-1β
IL-6
IL-12
Proinflammatory effects
IL-8
RANTES
MIP-1α
MIP-1β
Chemotactic effects (Nutrophil, Immature DC (NK cell)
Complement and coagulation cascade
Costimulatory molecules
CD40
CD80
CD86
T cell stimulation
Inflammatory cytokines
IFN-α
IFN-β
Antiviral effects
Cytokine-cytokine receptor interaction
IP-10
MIG
I-TAC
Chemotactic effects (T cell)
04620 5/25/12
(c) Kanehisa Laboratories

o

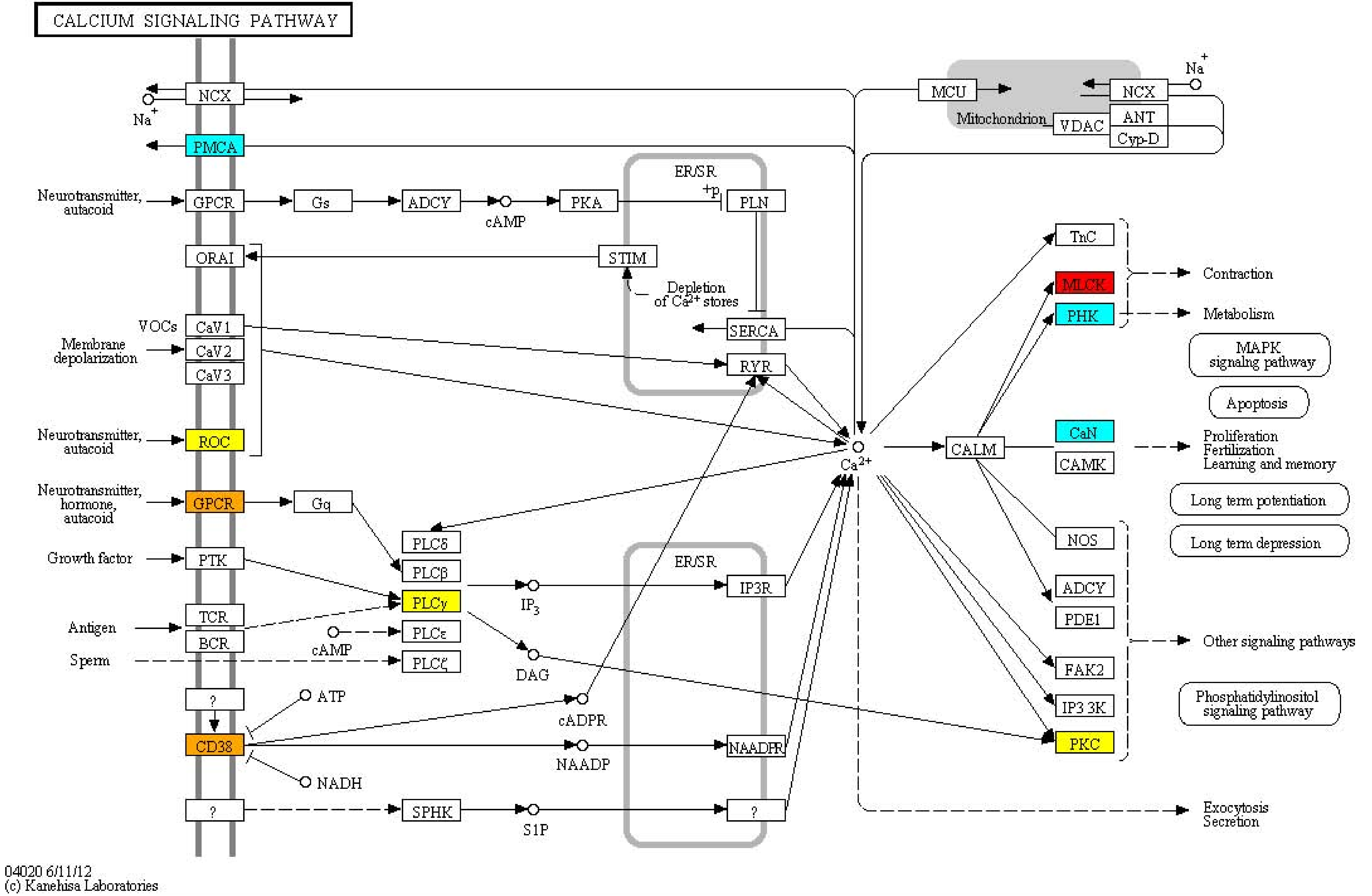
CALCIUM SIGNALING PATHWAY
NCX
Na+
PMCA
Neurotransmitter, autacoid
GPCR
Gs
ADCY
cAMP
PKA
ER/SR
+p
PLN
ORAI
STIM
Depletion of Ca2+ stores
VOCs
CaV1
CaV2
CaV3
Membrane depolarization
SERCA
RYR
Neurotransmitter, autacoid
ROC
Neurotransmitter, hormone, autacoid
GPCR
Gq
Growth factor
PTK
PLCδ
PLCβ
PLCγ
PLCε
PLCζ
Antigen
TCR
BCR
Sperm
cAMP
IP3
IP3R
DAG
?
ATP
CD38
cADPR
NAADP
NAADPR
NADH
?
SPHK
S1P
?
MCU
Mitochondrion
VDAC
NCX
ANT
Cyp-D
Na+
TnC
MLCK
PHK
Contraction
Metabolism
MAPK signaling pathway
Apoptosis
Ca2+
CALM
CaN
CAMK
Proliferation
Fertilization
Learning and memory
Long term potentiation
Long term depression
NOS
ADCY
PDE1
Other signaling pathways
FAK2
IP3 3K
PKC
Phosphatidylinositol signaling pathway
Exocytosis
Secretion
04020 6/11/12
(c) Kanehisa Laboratories

P

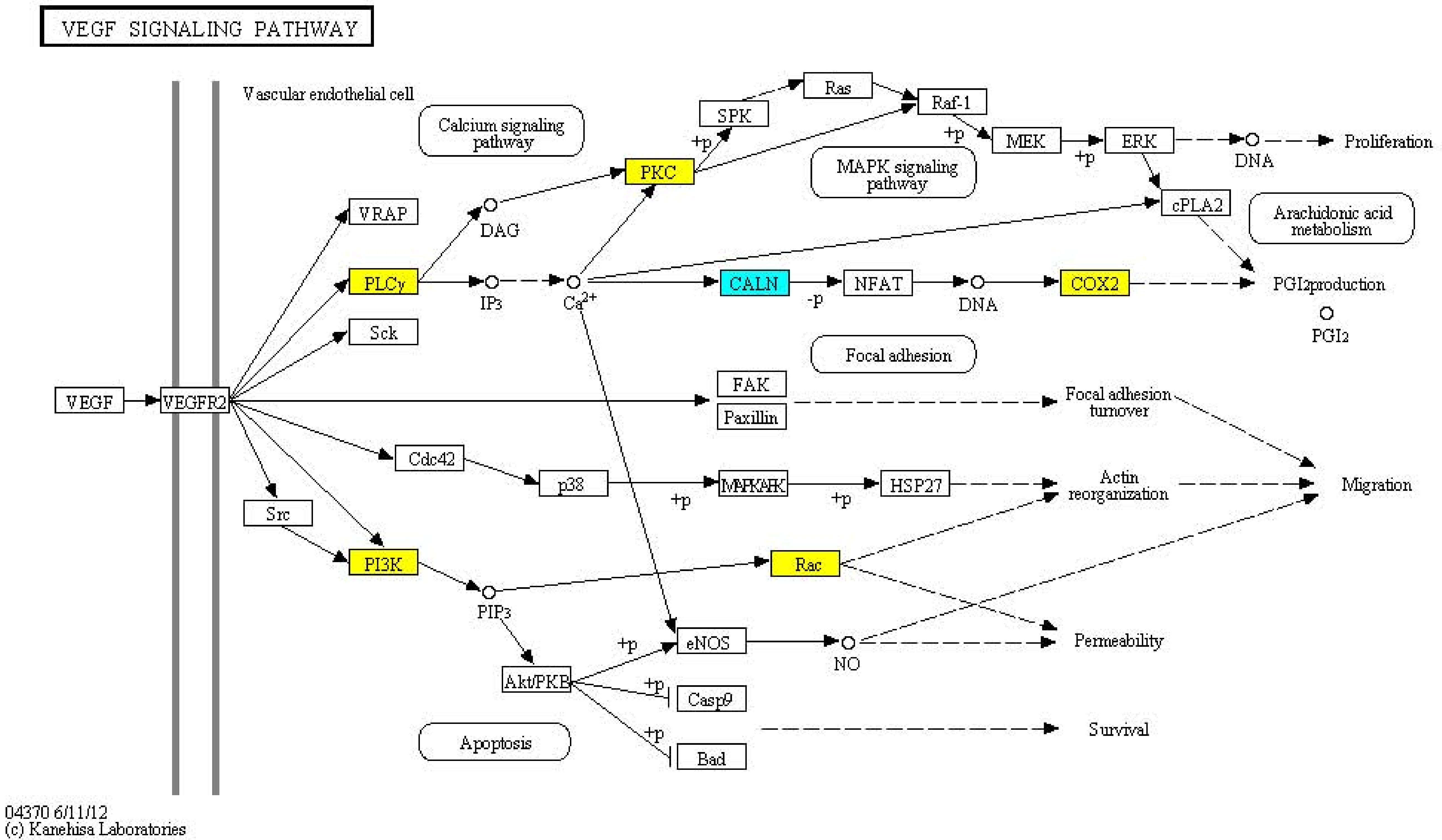

VEGF SIGNALING PATHWAY
Vascular endothelial cell
Calcium signaling pathway
MAPK signaling pathway
Arachidonic acid metabolism
Focal adhesion
Apoptosis
VEGF
VEGFR2
VRAP
PLCγ
Sck
Src
PI3K
Cdc42
p38
MAPKAPK
HSP27
DAG
IP3
Ca2+
PKC
SPK
Ras
Raf-1
MEK
ERK
DNA
Proliferation
cPLA2
CALN
NFAT
DNA
COX2
PGI2production
PGI2
FAK
Paxillin
Focal adhesion turnover
Actin reorganization
Migration
Rac
PIP3
Akt/PKB
eNOS
NO
Casp9
Bad
Permeability
Survival
+p
-p
04370 6/11/12
(c) Kanehisa Laboratories

Q

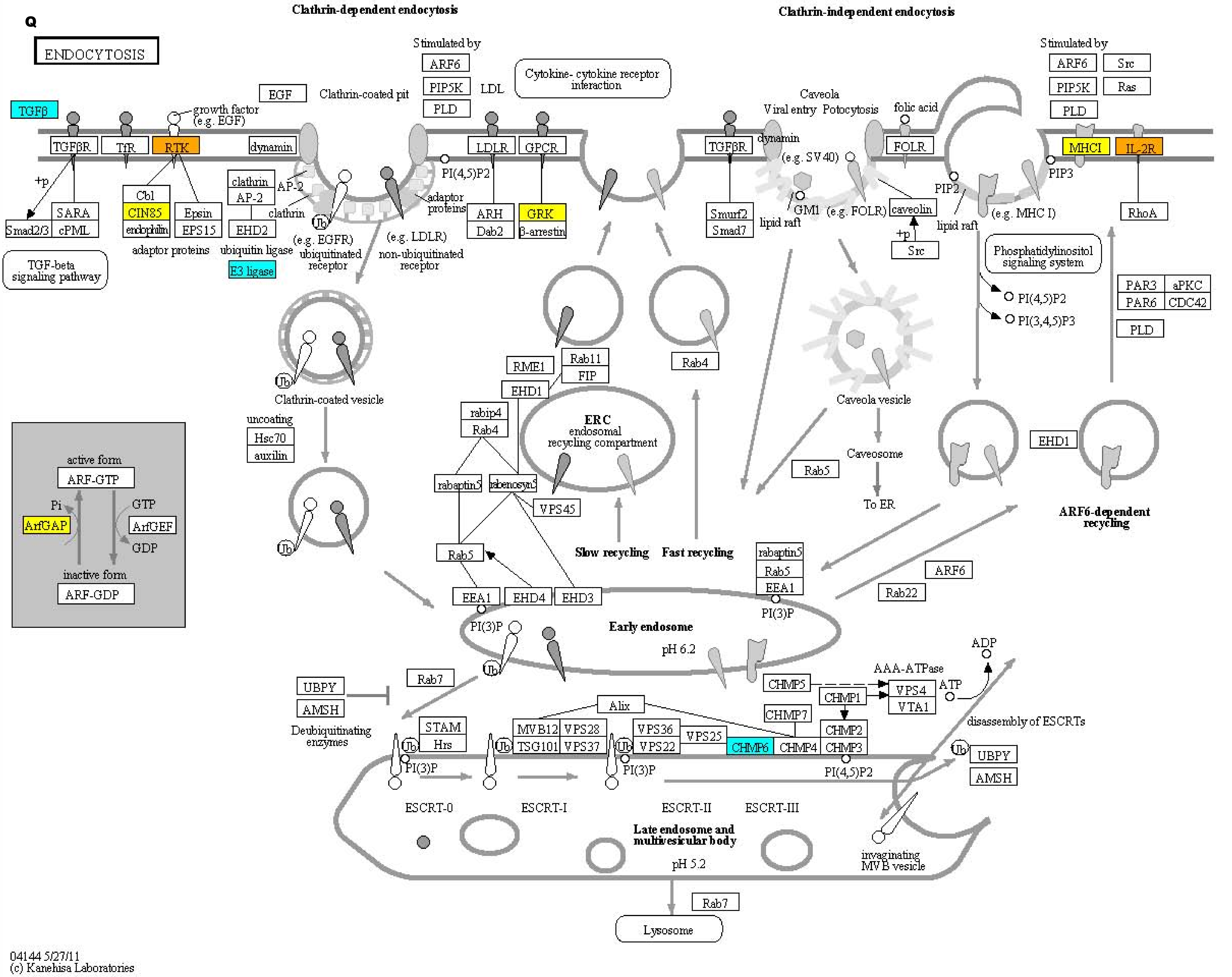
ENDOCYTOSIS
Clathrin-dependent endocytosis
Clathrin-independent endocytosis
TGFβ
growth factor (e.g. EGF)
TGFβR
TfR
RTK
EGF
Clathrin-coated pit
dynamin
Stimulated by
ARF6
PIP5K
PLD
LDL
Cytokine- cytokine receptor interaction
LDLR
GPCR
PI(4,5)P2
+p
Smad2/3
SARA
cPML
Cbl
CIN85
endophilin
Epsin
EPS15
adaptor proteins
clathrin
AP-2
EHD2
ubiquitin ligase
E3 ligase
adaptor proteins
(e.g. EGFR) ubiquitinated receptor
(e.g. LDLR) non-ubiquitinated receptor
ARH
Dab2
GRK
β-arrestin
TGF-beta signaling pathway
Ub
Clathrin-coated vesicle
uncoating
Hsc70
auxilin
active form
ARF-GTP
Pi
GTP
ArfGAP
ArfGEF
GDP
inactive form
ARF-GDP
RME1
Rab11
FIP
EHD1
rabip4
Rab4
ERC endosomal recycling compartment
rabaptin5
rabenosyn5
VPS45
Rab5
Slow recycling
Fast recycling
EEA1
EHD4
EHD3
PI(3)P
Early endosome
pH 6.2
Caveola
Viral entry
Potocytosis
(e.g. SV40)
folic acid
FOLR
GM1
(e.g. FOLR)
caveolin
lipid raft
Smurf2
Smad7
Src
Caveola vesicle
Caveosome
To ER
Src
Ras
MHCI
IL-2R
PIP2
PIP3
(e.g. MHC I)
RhoA
Phosphatidylinositol signaling system
PI(4,5)P2
PI(3,4,5)P3
PAR3
aPKC
PAR6
CDC42
PLD
EHD1
ARF6-dependent recycling
ARF6
Rab22
Rab7
UBPY
AMSH
Deubiquitinating enzymes
STAM
Hrs
Alix
MVB12
VPS28
TSG101
VPS37
VPS36
VPS22
VPS25
CHMP6
CHMP4
CHMP5
CHMP1
CHMP7
CHMP2
CHMP3
AAA-ATPase
VPS4
VTA1
ATP
ADP
disassembly of ESCRTs
ESCRT-0
ESCRT-I
ESCRT-II
ESCRT-III
Late endosome and multivesicular body
pH 5.2
invaginating MVB vesicle
Rab7
Lysosome
04144 5/27/11
(c) Kanehisa Laboratories

R

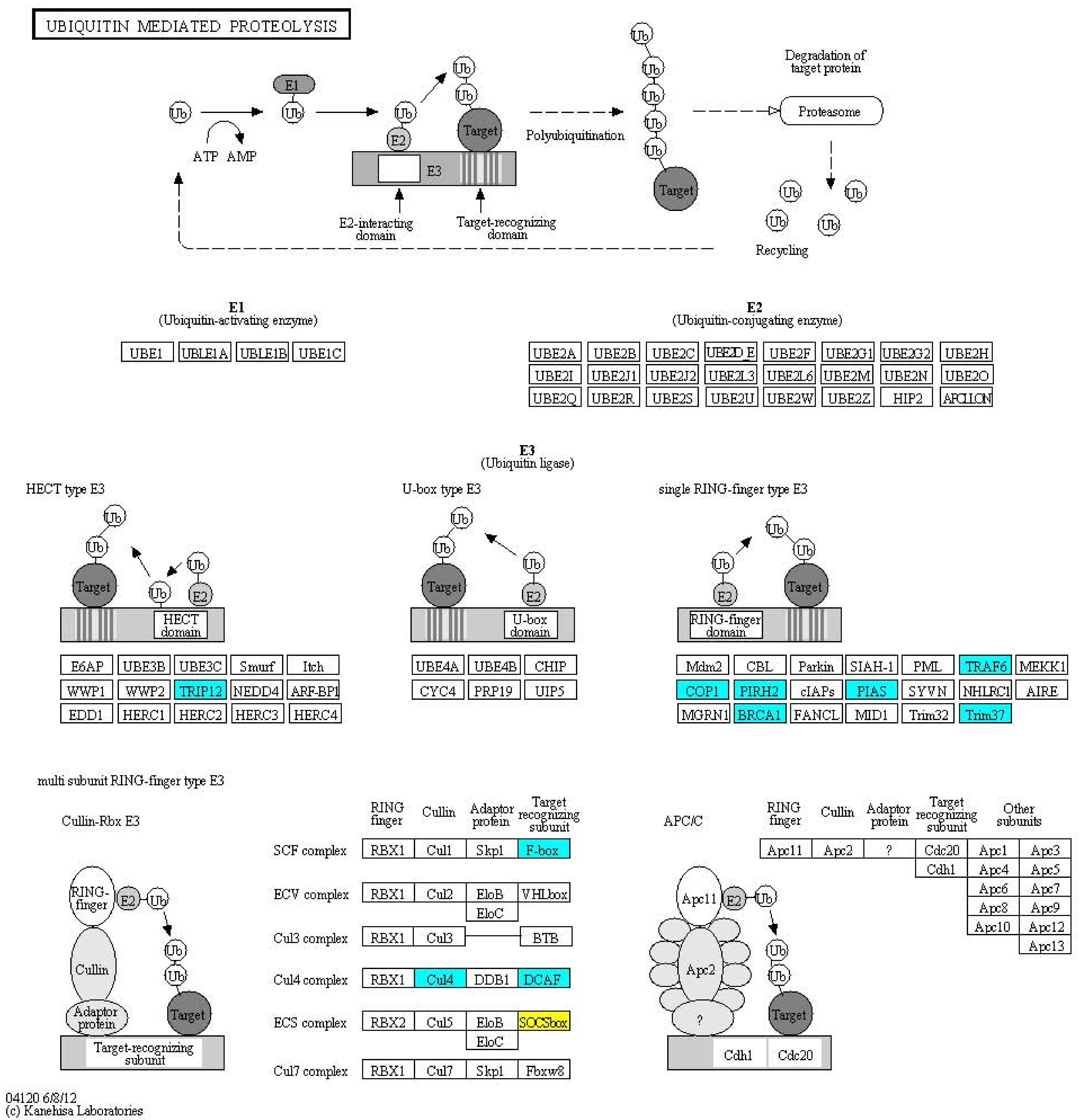
UBIQUITIN MEDIATED PROTEOLYSIS
E1
Ub
ATP AMP
E2
Target
E3
E2-interacting domain
Target-recognizing domain
Polyubiquitination
Degradation of target protein
Proteasome
Recycling
E1
(Ubiquitin-activating enzyme)
UBE1
UBLE1A
UBLE1B
UBE1C
E2
(Ubiquitin-conjugating enzyme)
UBE2A
UBE2B
UBE2C
UBE2D_E
UBE2F
UBE2G1
UBE2G2
UBE2H
UBE2I
UBE2J1
UBE2J2
UBE2L3
UBE2L6
UBE2M
UBE2N
UBE2O
UBE2Q
UBE2R
UBE2S
UBE2U
UBE2W
UBE2Z
HIP2
APOLLON
E3
(Ubiquitin ligase)
HECT type E3
HECT domain
E6AP
UBE3B
UBE3C
Smurf
Itch
WWP1
WWP2
TRIP12
NEDD4
ARF-BP1
EDD1
HERC1
HERC2
HERC3
HERC4
U-box type E3
U-box domain
UBE4A
UBE4B
CHIP
CYC4
PRP19
UIP5
single RING-finger type E3
RING-finger domain
Mdm2
CBL
Parkin
SIAH-1
PML
TRAF6
MEKK1
COP1
PIRH2
cIAPs
PIAS
SYVN
NHLRC1
AIRE
MGRN1
BRCA1
FANCL
MID1
Trim32
Trim37
multi subunit RING-finger type E3
Cullin-Rbx E3
RING-finger
Cullin
Adaptor protein
Target-recognizing subunit
RING finger
Cullin
Adaptor protein
Target recognizing subunit
SCF complex
RBX1
Cul1
Skp1
F-box
ECV complex
RBX1
Cul2
EloB
EloC
VHLbox
Cul3 complex
RBX1
Cul3
BTB
Cul4 complex
RBX1
Cul4
DDB1
DCAF
ECS complex
RBX2
Cul5
EloB
EloC
SOCSbox
Cul7 complex
RBX1
Cul7
Skp1
Fbxw8
APC/C
RING finger
Cullin
Adaptor protein
Target recognizing subunit
Other subunits
Apc11
Apc2
?
Cdc20
Cdh1
Apc1
Apc3
Apc4
Apc5
Apc6
Apc7
Apc8
Apc9
Apc10
Apc12
Apc13
Cdh1
Cdc20
04120 6/8/12
(c) Kanehisa Laboratories

s

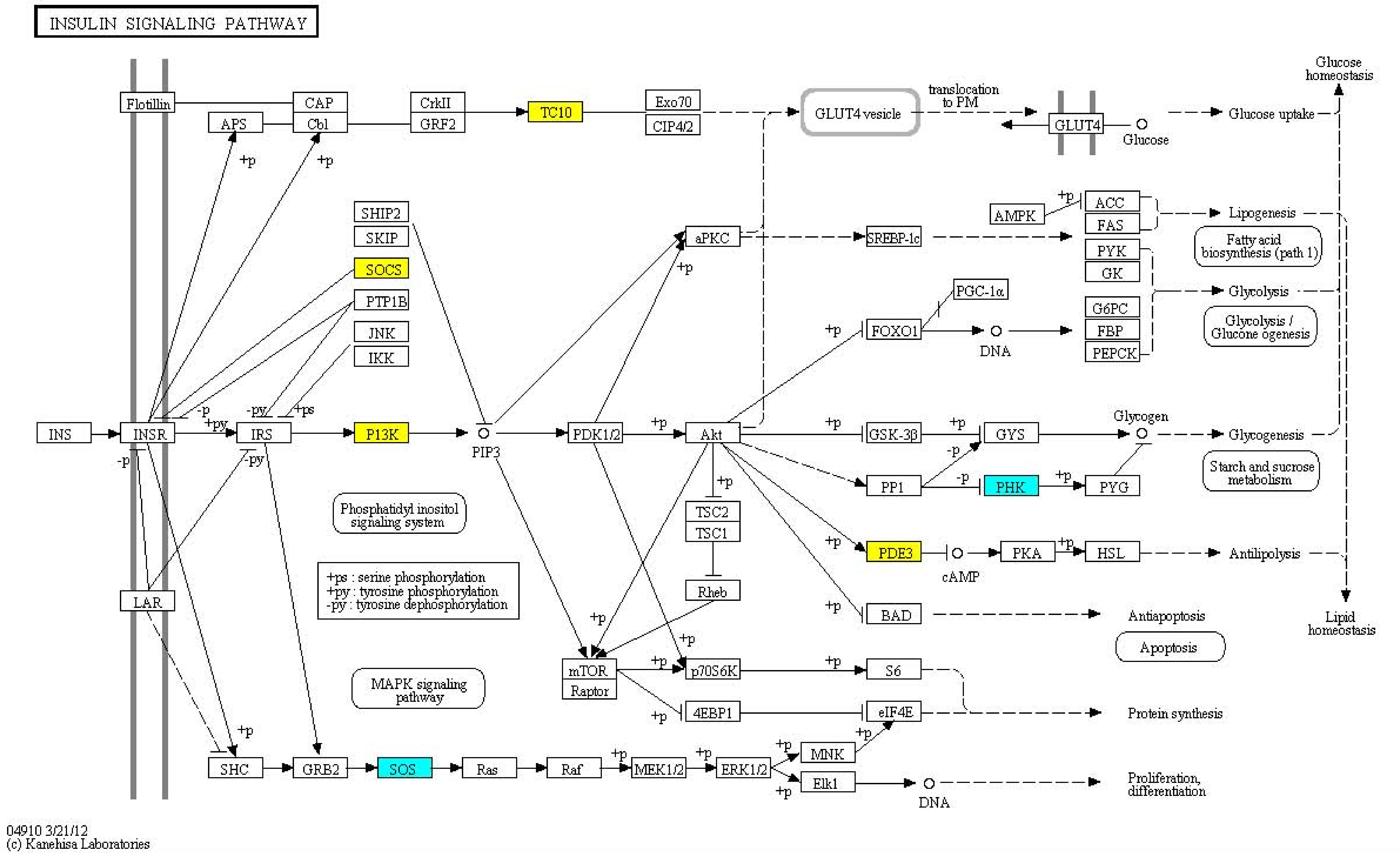
INSULIN SIGNALING PATHWAY
Flotillin
CAP
Cbl
APS
CrkII
GRF2
TC10
Exo70
CIP4/2
GLUT4 vesicle
translocation to PM
GLUT4
Glucose
Glucose uptake
Glucose homeostasis
SHIP2
SKIP
SOCS
PTP1B
JNK
IKK
aPKC
SREBP-1c
AMPK
ACC
FAS
Lipogenesis
Fatty acid biosynthesis (path 1)
PYK
GK
PGC-1α
FOXO1
DNA
G6PC
FBP
PEPCK
Glycolysis
Glycolysis / Gluconeogenesis
INS
INSR
IRS
P13K
PIP3
PDK1/2
Akt
GSK-3β
GYS
Glycogen
Glycogenesis
PP1
PHK
PYG
Starch and sucrose metabolism
TSC2
TSC1
Rheb
PDE3
cAMP
PKA
HSL
Antilipolysis
BAD
Antiapoptosis
Apoptosis
Lipid homeostasis
Phosphatidyl inositol signaling system
+ps : serine phosphorylation
+py : tyrosine phosphorylation
-py : tyrosine dephosphorylation
LAR
mTOR
Raptor
p70S6K
S6
4EBP1
eIF4E
Protein synthesis
MAPK signaling pathway
SHC
GRB2
SOS
Ras
Raf
MEK1/2
ERK1/2
MNK
Elk1
DNA
Proliferation, differentiation
04910 3/21/12
(c) Kanehisa Laboratories

T

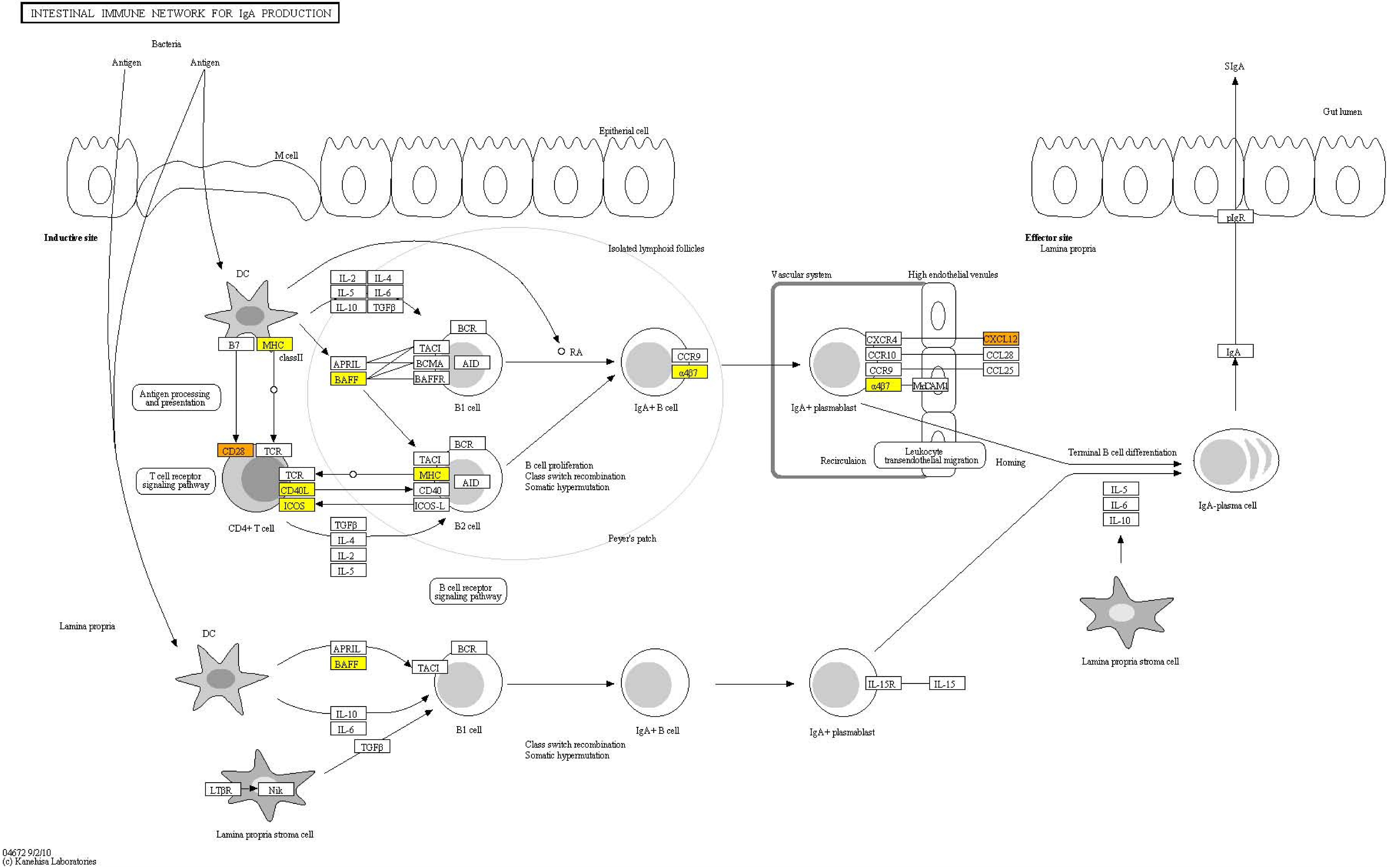
INTESTINAL IMMUNE NETWORK FOR IgA PRODUCTION
Bacteria
Antigen
Antigen
Epitherial cell
M cell
Gut lumen
SIgA
Inductive site
Effector site
Lamina propria
pIgR
Isolated lymphoid follicles
DC
IL-2
IL-4
IL-5
IL-6
IL-10
TGFβ
B7
MHC
classII
Antigen processing and presentation
APRIL
BAFF
TACI
BCMA
BAFFR
BCR
AID
B1 cell
RA
CCR9
α4β7
IgA+ B cell
Vascular system
High endothelial venules
CXCR4
CCR10
CCR9
α4β7
CXCL12
CCL28
CCL25
MAdCAM1
IgA+ plasmablast
IgA
Leukocyte transendothelial migration
Recirculaion
Homing
Terminal B cell differentiation
IgA-plasma cell
IL-5
IL-6
IL-10
CD28
TCR
T cell receptor signaling pathway
TCR
CD40L
ICOS
MHC
CD40
ICOS-L
TACI
BCR
AID
B2 cell
CD4+ T cell
B cell proliferation
Class switch recombination
Somatic hypermutation
TGFβ
IL-4
IL-2
IL-5
Peyer's patch
B cell receptor signaling pathway
Lamina propria stroma cell
Lamina propria
DC
APRIL
BAFF
TACI
BCR
B1 cell
IL-10
IL-6
TGFβ
IgA+ B cell
Class switch recombination
Somatic hypermutation
IgA+ plasmablast
IL-15R
IL-15
LTβR
Nik
Lamina propria stroma cell
04672 9/2/10
(c) Kanehisa Laboratories
