# Supplementary material for: Transcriptome Analysis in Chicken Cecal Epithelia upon Infection by Eimeria tenella In Vivo
Source: PLoS One. 2013 May 30;8(5):e64236. doi: 10.1371/journal.pone.0064236 (PMC3667848; doi:10.1371/journal.pone.0064236)
Supplement: Table S3 — List of KEGG pathways and associated genes. (PDF) [file pone.0064236.s005.pdf]

Additional file 3. Table S3. List of KEGG pathways and associated genes

gga04060 Cytokine-cytokine receptor interaction - Gallus gallus (chicken) (21)

gga:374229 TNFSF13B; tumor necrosis factor (ligand) superfamily, member 13b
gga:378783 KIT; v-kit Hardy-Zuckerman 4 feline sarcoma viral oncogene homolog (EC:2.7.10.1)
gga:395083 TNFRSF1B; tumor necrosis factor receptor superfamily, member 1B
gga:395180 CXCL12; chemokine (C-X-C motif) ligand 12
gga:395196 IL1B; interleukin 1, beta
gga:395199 IL2RG; interleukin 2 receptor, gamma (severe combined immunodeficiency)
gga:395312 IL18; interleukin 18 (interferon-gamma-inducing factor)
gga:395485 CD40LG; CD40 ligand
gga:395551 CCL4; chemokine (C-C motif) ligand 4
gga:395665 IFNAR1; interferon (alpha, beta and omega) receptor 1
gga:396054 IFNG; interferon, gamma
gga:396406 CSF1R; colony stimulating factor 1 receptor
gga:418666 CSF2RA; colony stimulating factor 2 receptor, alpha, low-affinity (granulocyte-macrophage)
gga:419424 TNFRSF18; tumor necrosis factor receptor superfamily, member 18
gga:420719 CX3CR1; chemokine (C-X3-C motif) receptor 1
gga:421686 IL20RA; interleukin 20 receptor, alpha
gga:422509 viral interleukin-8 homolog
gga:422510 CXCL13L2; chemokine
gga:426054 IL7R; interleukin 7 receptor
gga:427406 CCL19; chemokine (C-C motif) ligand 19
gga:769278 CCR6; chemokine (C-C motif) receptor 6

gga04514 Cell adhesion molecules (CAMs) - Gallus gallus (chicken) (21)

gga:374068 CDH5; cadherin 5, type 2 (vascular endothelium)
gga:374102 SDC2; syndecan 2
gga:386580 PTPRC; protein tyrosine phosphatase, receptor type, C (EC:3.1.3.48)
gga:395362 CD4; CD4 molecule
gga:395485 CD40LG; CD40 ligand
gga:396092 ALCAM; activated leukocyte cell adhesion molecule
gga:396181 ITGB2; integrin, beta 2 (complement component 3 receptor 3 and 4 subunit)
gga:396249 CD28; CD28 molecule
gga:403158 CD8A; CD8a molecule
gga:417050 BMA1; B locus M alpha chain 1
gga:419856 CD34; CD34 molecule
gga:424105 ICOS; inducible T-cell co-stimulator
gga:424106 CTLA4; cytotoxic T-lymphocyte-associated protein 4
gga:424121 ITGA4; integrin, alpha 4 (antigen CD49D, alpha 4 subunit of VLA-4 receptor)
gga:424467 VCAM1; vascular cell adhesion molecule 1
gga:425389 BF2; MHC BF2 class I
gga:427224 CD274; CD274 molecule
gga:428309 CNTNAP1; contactin associated protein 1
gga:693256 BLB2; MHC class II beta chain
gga:724083 BLB1; MHC class II antigen B-F minor heavy chain
gga:772097 CD226; CD226 molecule

gga01100 Metabolic pathways - Gallus gallus (chicken) (20)

gga:395139 ST3GAL2; ST3 beta-galactoside alpha-2,3-sialyltransferase 2 (EC:2.4.99.4)
gga:395389 ALDH1A3; aldehyde dehydrogenase 1 family, member A3 (EC:1.2.1.5)
gga:396169 ST6GAL1; ST6 beta-galactosamide alpha-2,6-sialyltranferase 1 (EC:2.4.99.1)
gga:396451 PTGS2; prostaglandin-endoperoxide synthase 2 (prostaglandin G/H synthase and cyclooxygenase) (EC:1.14.99.1)
gga:415786 CES1; carboxylesterase 1 (monocyte/macrophage serine esterase 1)
gga:415805 PLCG2; phospholipase C, gamma 2 (phosphatidylinositol-specific)
gga:416087 SUCLG2; succinate-CoA ligase, GDP-forming, beta subunit (EC:6.2.1.4)
gga:418104 ATP6V0A4; ATPase, H+ transporting, lysosomal V0 subunit a4
gga:418692 MGAT4A; mannosyl (alpha-1,3-)-glycoprotein beta-1,4-N-acetylglucosaminyltransferase, isozyme A (EC:2.4.1.145)
gga:419175 PLCG1; phospholipase C, gamma 1
gga:419403 NADK; NAD kinase (EC:2.7.1.23)
gga:419718 ST3GAL4; ST3 beta-galactoside alpha-2,3-sialyltransferase 4
gga:419793 SDHD; succinate dehydrogenase complex, subunit D, integral membrane protein
gga:420210 ATP6V0D2; ATPase, H+ transporting, lysosomal 38kDa, V0 subunit d2 (EC:3.6.3.14)
gga:421578 AGPAT4; 1-acylglycerol-3-phosphate O-acyltransferase 4 (lysophosphatidic acid acyltransferase, delta)
gga:422345 ACSL4; acyl-CoA synthetase long-chain family member 4
gga:422660 UGT2A3; UDP glucuronosyltransferase 2 family, polypeptide A3
gga:422827 CD38; CD38 molecule (EC:3.2.2.5)

gga:424028 UGT1A1; UDP glucuronosyltransferase 1 family, polypeptide A1
gga:426430 OAT; ornithine aminotransferase (EC:2.6.1.13)

gga04510 Focal adhesion - Gallus gallus (chicken) (19)

gga:395951 ITGA1; integrin, alpha 1
gga:396000 COL6A1; collagen, type VI, alpha 1
gga:396133 FN1; fibronectin 1
gga:396243 COL1A2; collagen, type I, alpha 2
gga:396292 COL6A2; collagen, type VI, alpha 2
gga:396294 FYN; FYN oncogene related to SRC, FGR, YES (EC:2.7.10.2)
gga:396340 COL3A1; collagen, type III, alpha 1
gga:396445 MYLK; myosin light chain kinase (EC:2.7.11.18)
gga:396548 COL6A3; collagen, type VI, alpha 3
gga:416567 PRKCB; protein kinase C, beta
gga:417319 PIK3R5; phosphoinositide 3-kinase regulatory subunit 5
gga:418044 RAC2; ras-related C3 botulinum toxin substrate 2 (rho family, small GTP binding protein Rac2)
gga:418236 PARVG; parvin, gamma
gga:418752 COL4A2; collagen, type IV, alpha 2
gga:418978 PDGFD; platelet derived growth factor D
gga:419031 VWF; von Willebrand factor
gga:419444 PIK3CD; phosphoinositide-3-kinase, catalytic, delta polypeptide (EC:2.7.1.153)
gga:424121 ITGA4; integrin, alpha 4 (antigen CD49D, alpha 4 subunit of VLA-4 receptor)
gga:427171 PIK3R1; phosphoinositide-3-kinase, regulatory subunit 1 (alpha)

gga04650 Natural killer cell mediated cytotoxicity - Gallus gallus (chicken) (18)

gga:100049618 FCER1G; Fc fragment of IgE, high affinity I, receptor for; gamma polypeptide
gga:395438 LCP2; lymphocyte cytosolic protein 2 (SH2 domain containing leukocyte protein of 76kDa)
gga:395665 IFNAR1; interferon (alpha, beta and omega) receptor 1
gga:395911 CD247; CD247 molecule
gga:396054 IFNG; interferon, gamma
gga:396181 ITGB2; integrin, beta 2 (complement component 3 receptor 3 and 4 subunit)
gga:396294 FYN; FYN oncogene related to SRC, FGR, YES (EC:2.7.10.2)
gga:415805 PLCG2; phospholipase C, gamma 2 (phosphatidylinositol-specific)
gga:416567 PRKCB; protein kinase C, beta
gga:417319 PIK3R5; phosphoinositide 3-kinase regulatory subunit 5
gga:418044 RAC2; ras-related C3 botulinum toxin substrate 2 (rho family, small GTP binding protein Rac2)
gga:418411 SH2D1B; SH2 domain containing 1B
gga:419175 PLCG1; phospholipase C, gamma 1
gga:419444 PIK3CD; phosphoinositide-3-kinase, catalytic, delta polypeptide (EC:2.7.1.153)
gga:420086 ZAP70; zeta-chain (TCR) associated protein kinase 70kDa
gga:425935 CD244; CD244 molecule, natural killer cell receptor 2B4
gga:427171 PIK3R1; phosphoinositide-3-kinase, regulatory subunit 1 (alpha)
gga:427272 SYK; spleen tyrosine kinase (EC:2.7.10.2)

gga05164 Influenza A - Gallus gallus (chicken) (16)

gga:395196 IL1B; interleukin 1, beta
gga:395312 IL18; interleukin 18 (interferon-gamma-inducing factor)
gga:395313 MX1; myxovirus (influenza virus) resistance 1, interferon-inducible protein p78 (mouse)
gga:395551 CCL4; chemokine (C-C motif) ligand 4
gga:395665 IFNAR1; interferon (alpha, beta and omega) receptor 1
gga:395681 JAK1; Janus kinase 1 (EC:2.7.10.2)
gga:396054 IFNG; interferon, gamma
gga:417050 BMA1; B locus M alpha chain 1
gga:417319 PIK3R5; phosphoinositide 3-kinase regulatory subunit 5
gga:418528 TMPRSS2; transmembrane protease, serine 2
gga:418638 TLR7; toll-like receptor 7
gga:419444 PIK3CD; phosphoinositide-3-kinase, catalytic, delta polypeptide (EC:2.7.1.153)
gga:424044 STAT1; signal transducer and activator of transcription 1, 91kDa
gga:427171 PIK3R1; phosphoinositide-3-kinase, regulatory subunit 1 (alpha)
gga:693256 BLB2; MHC class II beta chain
gga:724083 BLB1; MHC class II antigen B-F minor heavy chain

gga04810 Regulation of actin cytoskeleton - Gallus gallus (chicken) (15)

gga:395511 RDX; radixin
gga:395774 GSN; gelsolin
gga:395951 ITGA1; integrin, alpha 1
gga:396133 FN1; fibronectin 1

gga:396181 ITGB2; integrin, beta 2 (complement component 3 receptor 3 and 4 subunit)
gga:396445 MYLK; myosin light chain kinase (EC:2.7.11.18)
gga:416490 ARPC1B; actin related protein 2/3 complex, subunit 1B, 41kDa
gga:417319 PIK3R5; phosphoinositide 3-kinase regulatory subunit 5
gga:418044 RAC2; ras-related C3 botulinum toxin substrate 2 (rho family, small GTP binding protein Rac2)
gga:418978 PDGFD; platelet derived growth factor D
gga:419444 PIK3CD; phosphoinositide-3-kinase, catalytic, delta polypeptide (EC:2.7.1.153)
gga:420504 PIP4K2A; phosphatidylinositol-5-phosphate 4-kinase, type II, alpha (EC:2.7.1.149)
gga:422247 ARHGEF6; Rac/Cdc42 guanine nucleotide exchange factor (GEF) 6
gga:424121 ITGA4; integrin, alpha 4 (antigen CD49D, alpha 4 subunit of VLA-4 receptor)
gga:427171 PIK3R1; phosphoinositide-3-kinase, regulatory subunit 1 (alpha)

gga05168 Herpes simplex infection - Gallus gallus (chicken) (14)

gga:374055 TRAF5; TNF receptor-associated factor 5
gga:395196 IL1B; interleukin 1, beta
gga:395551 CCL4; chemokine (C-C motif) ligand 4
gga:395665 IFNAR1; interferon (alpha, beta and omega) receptor 1
gga:395681 JAK1; Janus kinase 1 (EC:2.7.10.2)
gga:396054 IFNG; interferon, gamma
gga:396370 C3; complement component 3 (EC:3.4.21.43)
gga:414741 CD74; CD74 molecule, major histocompatibility complex, class II invariant chain
gga:417050 BMA1; B locus M alpha chain 1
gga:417123 TRAF1; TNF receptor-associated factor 1
gga:424044 STAT1; signal transducer and activator of transcription 1, 91kDa
gga:425389 BF2; MHC BF2 class I
gga:693256 BLB2; MHC class II beta chain
gga:724083 BLB1; MHC class II antigen B-F minor heavy chain

gga04145 Phagosome - Gallus gallus (chicken) (13)

gga:396181 ITGB2; integrin, beta 2 (complement component 3 receptor 3 and 4 subunit)
gga:396370 C3; complement component 3 (EC:3.4.21.43)
gga:417050 BMA1; B locus M alpha chain 1
gga:417485 NCF1; neutrophil cytosolic factor 1
gga:418052 NCF4; neutrophil cytosolic factor 4, 40kDa
gga:418104 ATP6V0A4; ATPase, H+ transporting, lysosomal V0 subunit a4
gga:418581 CYBB; cytochrome b-245, beta polypeptide
gga:420210 ATP6V0D2; ATPase, H+ transporting, lysosomal 38kDa, V0 subunit d2 (EC:3.6.3.14)
gga:421037 TUBB6; tubulin, beta 6 class V
gga:424445 NCF2; neutrophil cytosolic factor 2
gga:425389 BF2; MHC BF2 class I
gga:693256 BLB2; MHC class II beta chain
gga:724083 BLB1; MHC class II antigen B-F minor heavy chain

gga04512 ECM-receptor interaction - Gallus gallus (chicken) (12)

gga:374102 SDC2; syndecan 2
gga:395666 CD44; CD44 molecule (Indian blood group)
gga:395951 ITGA1; integrin, alpha 1
gga:396000 COL6A1; collagen, type VI, alpha 1
gga:396133 FN1; fibronectin 1
gga:396243 COL1A2; collagen, type I, alpha 2
gga:396292 COL6A2; collagen, type VI, alpha 2
gga:396340 COL3A1; collagen, type III, alpha 1
gga:396548 COL6A3; collagen, type VI, alpha 3
gga:418752 COL4A2; collagen, type IV, alpha 2
gga:419031 VWF; von Willebrand factor
gga:424121 ITGA4; integrin, alpha 4 (antigen CD49D, alpha 4 subunit of VLA-4 receptor)

gga04630 Jak-STAT signaling pathway - Gallus gallus (chicken) (12)

gga:395199 IL2RG; interleukin 2 receptor, gamma (severe combined immunodeficiency)
gga:395665 IFNAR1; interferon (alpha, beta and omega) receptor 1
gga:395681 JAK1; Janus kinase 1 (EC:2.7.10.2)
gga:396054 IFNG; interferon, gamma
gga:416630 SOCS1; suppressor of cytokine signaling 1
gga:417319 PIK3R5; phosphoinositide 3-kinase regulatory subunit 5
gga:418666 CSF2RA; colony stimulating factor 2 receptor, alpha, low-affinity (granulocyte-macrophage)
gga:419444 PIK3CD; phosphoinositide-3-kinase, catalytic, delta polypeptide (EC:2.7.1.153)
gga:421686 IL20RA; interleukin 20 receptor, alpha

gga:424044 STAT1; signal transducer and activator of transcription 1, 91kDa
gga:426054 IL7R; interleukin 7 receptor
gga:427171 PIK3R1; phosphoinositide-3-kinase, regulatory subunit 1 (alpha)

gga04620 Toll-like receptor signaling pathway - Gallus gallus (chicken) (10)

gga:395196 IL1B; interleukin 1, beta
gga:395551 CCL4; chemokine (C-C motif) ligand 4
gga:395665 IFNAR1; interferon (alpha, beta and omega) receptor 1
gga:417319 PIK3R5; phosphoinositide 3-kinase regulatory subunit 5
gga:418638 TLR7; toll-like receptor 7
gga:419444 PIK3CD; phosphoinositide-3-kinase, catalytic, delta polypeptide (EC:2.7.1.153)
gga:420189 LY96; lymphocyte antigen 96
gga:424044 STAT1; signal transducer and activator of transcription 1, 91kDa
gga:426274 TLR6; toll-like receptor 6
gga:427171 PIK3R1; phosphoinositide-3-kinase, regulatory subunit 1 (alpha)

gga04672 Intestinal immune network for IgA production - Gallus gallus (chicken) (9)

gga:374229 TNFSF13B; tumor necrosis factor (ligand) superfamily, member 13b
gga:395180 CXCL12; chemokine (C-X-C motif) ligand 12
gga:395485 CD40LG; CD40 ligand
gga:396249 CD28; CD28 molecule
gga:417050 BMA1; B locus M alpha chain 1
gga:424105 ICOS; inducible T-cell co-stimulator
gga:424121 ITGA4; integrin, alpha 4 (antigen CD49D, alpha 4 subunit of VLA-4 receptor)
gga:693256 BLB2; MHC class II beta chain
gga:724083 BLB1; MHC class II antigen B-F minor heavy chain

gga04080 Neuroactive ligand-receptor interaction - Gallus gallus (chicken) (9)

gga:395108 GZMA; granzyme A (granzyme 1, cytotoxic T-lymphocyte-associated serine esterase 3) (EC:3.4.21.78)
gga:395507 P2RX5; purinergic receptor P2X, ligand-gated ion channel, 5
gga:396114 P2RY6; pyrimidinergic receptor P2Y, G-protein coupled, 6
gga:408082 EDNRB; endothelin receptor type B
gga:418198 C3AR1; complement component 3a receptor 1
gga:418665 P2RY8; purinergic receptor P2Y, G-protein coupled, 8
gga:424833 GPR35; G protein-coupled receptor 35
gga:426049 CTSG; cathepsin G
gga:428525 histamine H3 receptor-like

gga04010 MAPK signaling pathway - Gallus gallus (chicken) (8)

gga:395196 IL1B; interleukin 1, beta
gga:416567 PRKCB; protein kinase C, beta
gga:418044 RAC2; ras-related C3 botulinum toxin substrate 2 (rho family, small GTP binding protein Rac2)
gga:421340 DUSP10; dual specificity phosphatase 10
gga:421460 RASGRP3; RAS guanyl releasing protein 3 (calcium and DAG-regulated)
gga:422380 NRK; Nik related kinase (EC:2.7.11.1)
gga:429929 GADD45B; growth arrest and DNA-damage-inducible, beta
gga:770905 PTPN7; protein tyrosine phosphatase, non-receptor type 7

gga04370 VEGF signaling pathway - Gallus gallus (chicken) (8)

gga:396451 PTGS2; prostaglandin-endoperoxide synthase 2 (prostaglandin G/H synthase and cyclooxygenase) (EC:1.14.99.1)
gga:415805 PLCG2; phospholipase C, gamma 2 (phosphatidylinositol-specific)
gga:416567 PRKCB; protein kinase C, beta
gga:417319 PIK3R5; phosphoinositide 3-kinase regulatory subunit 5
gga:418044 RAC2; ras-related C3 botulinum toxin substrate 2 (rho family, small GTP binding protein Rac2)
gga:419175 PLCG1; phospholipase C, gamma 1
gga:419444 PIK3CD; phosphoinositide-3-kinase, catalytic, delta polypeptide (EC:2.7.1.153)
gga:427171 PIK3R1; phosphoinositide-3-kinase, regulatory subunit 1 (alpha)

gga04070 Phosphatidylinositol signaling system - Gallus gallus (chicken) (8)

gga:415805 PLCG2; phospholipase C, gamma 2 (phosphatidylinositol-specific)
gga:416567 PRKCB; protein kinase C, beta
gga:417319 PIK3R5; phosphoinositide 3-kinase regulatory subunit 5
gga:419175 PLCG1; phospholipase C, gamma 1
gga:419444 PIK3CD; phosphoinositide-3-kinase, catalytic, delta polypeptide (EC:2.7.1.153)
gga:420504 PIP4K2A; phosphatidylinositol-5-phosphate 4-kinase, type II, alpha (EC:2.7.1.149)

gga:424745 INPP5D; inositol polyphosphate-5-phosphatase, 145kDa
gga:427171 PIK3R1; phosphoinositide-3-kinase, regulatory subunit 1 (alpha)

gga04144 Endocytosis - Gallus gallus (chicken) (8)

gga:378783 KIT; v-kit Hardy-Zuckerman 4 feline sarcoma viral oncogene homolog (EC:2.7.10.1)
gga:395199 IL2RG; interleukin 2 receptor, gamma (severe combined immunodeficiency)
gga:396406 CSF1R; colony stimulating factor 1 receptor
gga:416904 ADRBK2; adrenergic, beta, receptor kinase 2
gga:418608 SH3KBP1; SH3-domain kinase binding protein 1
gga:419641 SMAP2; small ArfGAP2
gga:422879 GRK4; G protein-coupled receptor kinase 4 (EC:2.7.1.-)
gga:425389 BF2; MHC BF2 class I

gga04020 Calcium signaling pathway - Gallus gallus (chicken) (7)

gga:395507 P2RX5; purinergic receptor P2X, ligand-gated ion channel, 5
gga:396445 MYLK; myosin light chain kinase (EC:2.7.11.18)
gga:408082 EDNRB; endothelin receptor type B
gga:415805 PLCG2; phospholipase C, gamma 2 (phosphatidylinositol-specific)
gga:416567 PRKCB; protein kinase C, beta
gga:419175 PLCG1; phospholipase C, gamma 1
gga:422827 CD38; CD38 molecule (EC:3.2.2.5)

gga04012 ErbB signaling pathway - Gallus gallus (chicken) (7)

gga:373906 NRG1; neuregulin 1
gga:415805 PLCG2; phospholipase C, gamma 2 (phosphatidylinositol-specific)
gga:416567 PRKCB; protein kinase C, beta
gga:417319 PIK3R5; phosphoinositide 3-kinase regulatory subunit 5
gga:419175 PLCG1; phospholipase C, gamma 1
gga:419444 PIK3CD; phosphoinositide-3-kinase, catalytic, delta polypeptide (EC:2.7.1.153)
gga:427171 PIK3R1; phosphoinositide-3-kinase, regulatory subunit 1 (alpha)

gga04142 Lysosome - Gallus gallus (chicken) (6)

gga:418104 ATP6V0A4; ATPase, H+ transporting, lysosomal V0 subunit a4
gga:418621 AP1S2; adaptor-related protein complex 1, sigma 2 subunit
gga:419552 LAPTM5; lysosomal protein transmembrane 5
gga:420210 ATP6V0D2; ATPase, H+ transporting, lysosomal 38kDa, V0 subunit d2 (EC:3.6.3.14)
gga:423789 LIPA; lipase A, lysosomal acid, cholesterol esterase (Wolman disease)
gga:426049 CTSG; cathepsin G

gga04520 Adherens junction - Gallus gallus (chicken) (6)

gga:395229 TCF7; transcription factor 7 (T-cell specific, HMG-box)
gga:396238 YES1; v-yes-1 Yamaguchi sarcoma viral oncogene homolog 1 (EC:2.7.10.2)
gga:396294 FYN; FYN oncogene related to SRC, FGR, YES (EC:2.7.10.2)
gga:416184 CTNNA1; catenin (cadherin-associated protein), alpha 1, 102kDa
gga:418044 RAC2; ras-related C3 botulinum toxin substrate 2 (rho family, small GTP binding protein Rac2)
gga:432368 SNAI2; snail homolog 2 (Drosophila)

gga04270 Vascular smooth muscle contraction - Gallus gallus (chicken) (6)

gga:373965 CALD1; caldesmon 1
gga:395301 KCNMB1; potassium large conductance calcium-activated channel, subfamily M, beta member 1
gga:396445 MYLK; myosin light chain kinase (EC:2.7.11.18)
gga:416567 PRKCB; protein kinase C, beta
gga:423518 PRKCH; protein kinase C, eta
gga:771678 GUCY1A2; guanylate cyclase 1, soluble, alpha 2

gga04910 Insulin signaling pathway - Gallus gallus (chicken) (6)

gga:395114 RHOQ; ras homolog gene family, member Q
gga:416630 SOCS1; suppressor of cytokine signaling 1
gga:417319 PIK3R5; phosphoinositide 3-kinase regulatory subunit 5
gga:419444 PIK3CD; phosphoinositide-3-kinase, catalytic, delta polypeptide (EC:2.7.1.153)
gga:423064 PDE3B; phosphodiesterase 3B, cGMP-inhibited (EC:3.1.4.17)
gga:427171 PIK3R1; phosphoinositide-3-kinase, regulatory subunit 1 (alpha)

gga05132 Salmonella infection - Gallus gallus (chicken) (5)

gga:395196 IL1B; interleukin 1, beta
gga:395312 IL18; interleukin 18 (interferon-gamma-inducing factor)
gga:396054 IFNG; interferon, gamma
gga:416490 ARPC1B; actin related protein 2/3 complex, subunit 1B, 41kDa
gga:423484 KLC1; kinesin light chain 1

gga00562 Inositol phosphate metabolism - Gallus gallus (chicken) (5)

gga:415805 PLCG2; phospholipase C, gamma 2 (phosphatidylinositol-specific)
gga:419175 PLCG1; phospholipase C, gamma 1
gga:419444 PIK3CD; phosphoinositide-3-kinase, catalytic, delta polypeptide (EC:2.7.1.153)
gga:420504 PIP4K2A; phosphatidylinositol-5-phosphate 4-kinase, type II, alpha (EC:2.7.1.149)
gga:424745 INPP5D; inositol polyphosphate-5-phosphatase, 145kDa

gga04540 Gap junction - Gallus gallus (chicken) (5)

gga:395278 GJA1; gap junction protein, alpha 1, 43kDa
gga:416567 PRKCB; protein kinase C, beta
gga:418978 PDGFD; platelet derived growth factor D
gga:421037 TUBB6; tubulin, beta 6 class V
gga:771678 GUCY1A2; guanylate cyclase 1, soluble, alpha 2

gga04310 Wnt signaling pathway - Gallus gallus (chicken) (5)

gga:395229 TCF7; transcription factor 7 (T-cell specific, HMG-box)
gga:416567 PRKCB; protein kinase C, beta
gga:418044 RAC2; ras-related C3 botulinum toxin substrate 2 (rho family, small GTP binding protein Rac2)
gga:418983 MMP7; matrix metallopeptidase 7 (matrilysin, uterine) (EC:3.4.24.23)
gga:423958 CTBP2; C-terminal binding protein 2

gga04530 Tight junction - Gallus gallus (chicken) (5)

gga:396238 YES1; v-yes-1 Yamaguchi sarcoma viral oncogene homolog 1 (EC:2.7.10.2)
gga:416184 CTNNA1; catenin (cadherin-associated protein), alpha 1, 102kDa
gga:416567 PRKCB; protein kinase C, beta
gga:423518 PRKCH; protein kinase C, eta
gga:426867 HCLS1; hematopoietic cell-specific Lyn substrate 1

gga03320 PPAR signaling pathway - Gallus gallus (chicken) (4)

gga:418982 MMP1; matrix metallopeptidase 1 (interstitial collagenase)
gga:420197 FABP5; fatty acid binding protein 5 (psoriasis-associated)
gga:422345 ACSL4; acyl-CoA synthetase long-chain family member 4
gga:423118 CPT1A; carnitine palmitoyltransferase 1A (liver) (EC:2.3.1.21)

gga04916 Melanogenesis - Gallus gallus (chicken) (4)

gga:378783 KIT; v-kit Hardy-Zuckerman 4 feline sarcoma viral oncogene homolog (EC:2.7.10.1)
gga:395229 TCF7; transcription factor 7 (T-cell specific, HMG-box)
gga:408082 EDNRB; endothelin receptor type B
gga:416567 PRKCB; protein kinase C, beta

gga04914 Progesterone-mediated oocyte maturation - Gallus gallus (chicken) (4)

gga:417319 PIK3R5; phosphoinositide 3-kinase regulatory subunit 5
gga:419444 PIK3CD; phosphoinositide-3-kinase, catalytic, delta polypeptide (EC:2.7.1.153)
gga:423064 PDE3B; phosphodiesterase 3B, cGMP-inhibited (EC:3.1.4.17)
gga:427171 PIK3R1; phosphoinositide-3-kinase, regulatory subunit 1 (alpha)

gga04210 Apoptosis - Gallus gallus (chicken) (4)

gga:395196 IL1B; interleukin 1, beta
gga:417319 PIK3R5; phosphoinositide 3-kinase regulatory subunit 5
gga:419444 PIK3CD; phosphoinositide-3-kinase, catalytic, delta polypeptide (EC:2.7.1.153)
gga:427171 PIK3R1; phosphoinositide-3-kinase, regulatory subunit 1 (alpha)

gga00514 Other types of O-glycan biosynthesis - Gallus gallus (chicken) (3)

gga:396169 ST6GAL1; ST6 beta-galactosamide alpha-2,6-sialyltranferase 1 (EC:2.4.99.1)
gga:422660 UGT2A3; UDP glucuronosyltransferase 2 family, polypeptide A3

gga:424028 UGT1A1; UDP glucuronosyltransferase 1 family, polypeptide A1

gga00982 Drug metabolism - cytochrome P450 - Gallus gallus (chicken) (3)

gga:395389 ALDH1A3; aldehyde dehydrogenase 1 family, member A3 (EC:1.2.1.5)
gga:422660 UGT2A3; UDP glucuronosyltransferase 2 family, polypeptide A3
gga:424028 UGT1A1; UDP glucuronosyltransferase 1 family, polypeptide A1

gga04621 NOD-like receptor signaling pathway - Gallus gallus (chicken) (3)

gga:395196 IL1B; interleukin 1, beta
gga:395312 IL18; interleukin 18 (interferon-gamma-inducing factor)
gga:395551 CCL4; chemokine (C-C motif) ligand 4

gga04623 Cytosolic DNA-sensing pathway - Gallus gallus (chicken) (3)

gga:395196 IL1B; interleukin 1, beta
gga:395312 IL18; interleukin 18 (interferon-gamma-inducing factor)
gga:395551 CCL4; chemokine (C-C motif) ligand 4

gga00983 Drug metabolism - other enzymes - Gallus gallus (chicken) (3)

gga:415786 CES1; carboxylesterase 1 (monocyte/macrophage serine esterase 1)
gga:422660 UGT2A3; UDP glucuronosyltransferase 2 family, polypeptide A3
gga:424028 UGT1A1; UDP glucuronosyltransferase 1 family, polypeptide A1

gga00190 Oxidative phosphorylation - Gallus gallus (chicken) (3)

gga:418104 ATP6V0A4; ATPase, H+ transporting, lysosomal V0 subunit a4
gga:419793 SDHD; succinate dehydrogenase complex, subunit D, integral membrane protein
gga:420210 ATP6V0D2; ATPase, H+ transporting, lysosomal 38kDa, V0 subunit d2 (EC:3.6.3.14)

gga00860 Porphyrin and chlorophyll metabolism - Gallus gallus (chicken) (3)

gga:420776 BLVRA; biliverdin reductase A
gga:422660 UGT2A3; UDP glucuronosyltransferase 2 family, polypeptide A3
gga:424028 UGT1A1; UDP glucuronosyltransferase 1 family, polypeptide A1

gga04920 Adipocytokine signaling pathway - Gallus gallus (chicken) (3)

gga:395083 TNFRSF1B; tumor necrosis factor receptor superfamily, member 1B
gga:422345 ACSL4; acyl-CoA synthetase long-chain family member 4
gga:423118 CPT1A; carnitine palmitoyltransferase 1A (liver) (EC:2.3.1.21)

gga00980 Metabolism of xenobiotics by cytochrome P450 - Gallus gallus (chicken) (3)

gga:395389 ALDH1A3; aldehyde dehydrogenase 1 family, member A3 (EC:1.2.1.5)
gga:422660 UGT2A3; UDP glucuronosyltransferase 2 family, polypeptide A3
gga:424028 UGT1A1; UDP glucuronosyltransferase 1 family, polypeptide A1

gga04150 mTOR signaling pathway - Gallus gallus (chicken) (3)

gga:417319 PIK3R5; phosphoinositide 3-kinase regulatory subunit 5
gga:419444 PIK3CD; phosphoinositide-3-kinase, catalytic, delta polypeptide (EC:2.7.1.153)
gga:427171 PIK3R1; phosphoinositide-3-kinase, regulatory subunit 1 (alpha)

gga00760 Nicotinate and nicotinamide metabolism - Gallus gallus (chicken) (2)

gga:419403 NADK; NAD kinase (EC:2.7.1.23)
gga:422827 CD38; CD38 molecule (EC:3.2.2.5)

gga00020 Citrate cycle (TCA cycle) - Gallus gallus (chicken) (2)

gga:416087 SUCLG2; succinate-CoA ligase, GDP-forming, beta subunit (EC:6.2.1.4)
gga:419793 SDHD; succinate dehydrogenase complex, subunit D, integral membrane protein

gga00140 Steroid hormone biosynthesis - Gallus gallus (chicken) (2)

gga:422660 UGT2A3; UDP glucuronosyltransferase 2 family, polypeptide A3
gga:424028 UGT1A1; UDP glucuronosyltransferase 1 family, polypeptide A1

gga00533 Glycosaminoglycan biosynthesis - keratan sulfate - Gallus gallus (chicken) (2)

gga:395139 ST3GAL2; ST3 beta-galactoside alpha-2,3-sialyltransferase 2 (EC:2.4.99.4)
gga:429123 CHST2; carbohydrate (N-acetylglucosamine-6-O) sulfotransferase 2

gga00040 Pentose and glucuronate interconversions - Gallus gallus (chicken) (2)

gga:422660 UGT2A3; UDP glucuronosyltransferase 2 family, polypeptide A3
gga:424028 UGT1A1; UDP glucuronosyltransferase 1 family, polypeptide A1

gga00510 N-Glycan biosynthesis - Gallus gallus (chicken) (2)

gga:396169 ST6GAL1; ST6 beta-galactosamide alpha-2,6-sialyltranferase 1 (EC:2.4.99.1)
gga:418692 MGAT4A; mannosyl (alpha-1,3-)-glycoprotein beta-1,4-N-acetylglucosaminyltransferase, isozyme A (EC:2.4.1.145)

gga00053 Ascorbate and aldarate metabolism - Gallus gallus (chicken) (2)

gga:422660 UGT2A3; UDP glucuronosyltransferase 2 family, polypeptide A3
gga:424028 UGT1A1; UDP glucuronosyltransferase 1 family, polypeptide A1

gga04350 TGF-beta signaling pathway - Gallus gallus (chicken) (2)

gga:396054 IFNG; interferon, gamma
gga:417892 DCN; decorin

gga04110 Cell cycle - Gallus gallus (chicken) (2)

gga:422360 STAG2; stromal antigen 2
gga:429929 GADD45B; growth arrest and DNA-damage-inducible, beta

gga00500 Starch and sucrose metabolism - Gallus gallus (chicken) (2)

gga:422660 UGT2A3; UDP glucuronosyltransferase 2 family, polypeptide A3
gga:424028 UGT1A1; UDP glucuronosyltransferase 1 family, polypeptide A1

gga00830 Retinol metabolism - Gallus gallus (chicken) (2)

gga:422660 UGT2A3; UDP glucuronosyltransferase 2 family, polypeptide A3
gga:424028 UGT1A1; UDP glucuronosyltransferase 1 family, polypeptide A1

gga04115 p53 signaling pathway - Gallus gallus (chicken) (2)

gga:415948 SHISA5; shisa homolog 5 (Xenopus laevis)
gga:429929 GADD45B; growth arrest and DNA-damage-inducible, beta

gga00230 Purine metabolism - Gallus gallus (chicken) (2)

gga:423064 PDE3B; phosphodiesterase 3B, cGMP-inhibited (EC:3.1.4.17)
gga:771678 GUCY1A2; guanylate cyclase 1, soluble, alpha 2

gga00071 Fatty acid metabolism - Gallus gallus (chicken) (2)

gga:422345 ACSL4; acyl-CoA synthetase long-chain family member 4
gga:423118 CPT1A; carnitine palmitoyltransferase 1A (liver) (EC:2.3.1.21)

gga03450 Non-homologous end-joining - Gallus gallus (chicken) (1)

gga:423853 POLL; polymerase (DNA directed), lambda

gga03013 RNA transport - Gallus gallus (chicken) (1)

gga:415977 NUP210; nucleoporin 210kDa

gga00512 Mucin type O-Glycan biosynthesis - Gallus gallus (chicken) (1)

gga:395139 ST3GAL2; ST3 beta-galactoside alpha-2,3-sialyltransferase 2 (EC:2.4.99.4)

gga00640 Propanoate metabolism - Gallus gallus (chicken) (1)

gga:416087 SUCLG2; succinate-CoA ligase, GDP-forming, beta subunit (EC:6.2.1.4)

gga00120 Primary bile acid biosynthesis - Gallus gallus (chicken) (1)

gga:423788 CH25H; cholesterol 25-hydroxylase

gga00130 Ubiquinone and other terpenoid-quinone biosynthesis - Gallus gallus (chicken) (1)

gga:769737 NQO1; NAD(P)H dehydrogenase, quinone 1

gga04260 Cardiac muscle contraction - Gallus gallus (chicken) (1)

gga:396549 ATP1B3; ATPase, Na+/K+ transporting, beta 3 polypeptide (EC:3.6.3.9)

gga00340 Histidine metabolism - Gallus gallus (chicken) (1)

gga:395389 ALDH1A3; aldehyde dehydrogenase 1 family, member A3 (EC:1.2.1.5)

gga00360 Phenylalanine metabolism - Gallus gallus (chicken) (1)

gga:395389 ALDH1A3; aldehyde dehydrogenase 1 family, member A3 (EC:1.2.1.5)

gga00561 Glycerolipid metabolism - Gallus gallus (chicken) (1)

gga:421578 AGPAT4; 1-acylglycerol-3-phosphate O-acyltransferase 4 (lysophosphatidic acid acyltransferase, delta)

gga00100 Steroid biosynthesis - Gallus gallus (chicken) (1)

gga:423789 LIPA; lipase A, lysosomal acid, cholesterol esterase (Wolman disease)

gga04146 Peroxisome - Gallus gallus (chicken) (1)

gga:422345 ACSL4; acyl-CoA synthetase long-chain family member 4

gga00564 Glycerophospholipid metabolism - Gallus gallus (chicken) (1)

gga:421578 AGPAT4; 1-acylglycerol-3-phosphate O-acyltransferase 4 (lysophosphatidic acid acyltransferase, delta)

gga00532 Glycosaminoglycan biosynthesis - chondroitin sulfate - Gallus gallus (chicken) (1)

gga:423952 CHST15; carbohydrate (N-acetylgalactosamine 4-sulfate 6-O) sulfotransferase 15

gga04141 Protein processing in endoplasmic reticulum - Gallus gallus (chicken) (1)

gga:420867 TXNDC5; thioredoxin domain containing 5 (endoplasmic reticulum)

gga04320 Dorso-ventral axis formation - Gallus gallus (chicken) (1)

gga:396235 TNIP1; TNFAIP3 interacting protein 1

gga00410 beta-Alanine metabolism - Gallus gallus (chicken) (1)

gga:395389 ALDH1A3; aldehyde dehydrogenase 1 family, member A3 (EC:1.2.1.5)

gga04912 GnRH signaling pathway - Gallus gallus (chicken) (1)

gga:386583 MMP2; matrix metallopeptidase 2 (gelatinase A, 72kDa gelatinase, 72kDa type IV collagenase) (EC:3.4.24.24)

gga00603 Glycosphingolipid biosynthesis - globo series - Gallus gallus (chicken) (1)

gga:395139 ST3GAL2; ST3 beta-galactoside alpha-2,3-sialyltransferase 2 (EC:2.4.99.4)

gga00590 Arachidonic acid metabolism - Gallus gallus (chicken) (1)

gga:396451 PTGS2; prostaglandin-endoperoxide synthase 2 (prostaglandin G/H synthase and cyclooxygenase) (EC:1.14.99.1)

gga00062 Fatty acid elongation - Gallus gallus (chicken) (1)

gga:428646 ELOVL5; ELOVL fatty acid elongase 5

gga00604 Glycosphingolipid biosynthesis - ganglio series - Gallus gallus (chicken) (1)

gga:395139 ST3GAL2; ST3 beta-galactoside alpha-2,3-sialyltransferase 2 (EC:2.4.99.4)

gga00330 Arginine and proline metabolism - Gallus gallus (chicken) (1)

gga:426430 OAT; ornithine aminotransferase (EC:2.6.1.13)

gga04140 Regulation of autophagy - Gallus gallus (chicken) (1)

gga:396054 IFNG; interferon, gamma

gga00350 Tyrosine metabolism - Gallus gallus (chicken) (1)

gga:395389 ALDH1A3; aldehyde dehydrogenase 1 family, member A3 (EC:1.2.1.5)

gga01040 Biosynthesis of unsaturated fatty acids - Gallus gallus (chicken) (1)

gga:428646 ELOVL5; ELOVL fatty acid elongase 5

gga00010 Glycolysis / Gluconeogenesis - Gallus gallus (chicken) (1)

gga:395389 ALDH1A3; aldehyde dehydrogenase 1 family, member A3 (EC:1.2.1.5)

gga04120 Ubiquitin mediated proteolysis - Gallus gallus (chicken) (1)

gga:416630 SOCS1; suppressor of cytokine signaling 1

gga04614 Renin-angiotensin system - Gallus gallus (chicken) (1)

gga:426049 CTSG; cathepsin G

gga03410 Base excision repair - Gallus gallus (chicken) (1)

gga:423853 POLL; polymerase (DNA directed), lambda

gga00601 Glycosphingolipid biosynthesis - lacto and neolacto series - Gallus gallus (chicken) (1)

gga:419718 ST3GAL4; ST3 beta-galactoside alpha-2,3-sialyltransferase 4

gga03050 Proteasome - Gallus gallus (chicken) (1)

gga:396054 IFNG; interferon, gamma

gga04330 Notch signaling pathway - Gallus gallus (chicken) (1)

gga:423958 CTBP2; C-terminal binding protein 2
