# Supplementary material for: Transcriptome Analysis in Chicken Cecal Epithelia upon Infection by Eimeria tenella In Vivo
Source: PLoS One. 2013 May 30;8(5):e64236. doi: 10.1371/journal.pone.0064236 (PMC3667848; doi:10.1371/journal.pone.0064236)
Supplement: Table S4 — List of differentially expressed genes that could be manually assigned into major functional groups. Up-regulated genes are in red, while down-regulated genes are in blue font. (PDF) [file pone.0064236.s006.pdf]

Table S4. List of differentially expressed genes that could be manually assigned into major functional groups. Up-regulated genes are in red, while down-regulated genes are in blue font.

Mannually annotated group	Probe Set ID	Gene description	Fold change
Cell death/growth/differentiation	Gga.5710.2.S1_a_at	HtrA serine peptidase 3	16.757
Cell death/growth/differentiation	Gga.5164.1.S2_at	BCL2-related protein A1	13.157
Cell death/growth/differentiation	Gga.2582.1.S1_at	transforming growth factor, beta-induced, 68kDa	13.131
Cell death/growth/differentiation	Gga.1247.1.S2_at	B6.1	11.957
Cell death/growth/differentiation	Gga.744.1.S1_at	GATA binding protein 3	9.192
Cell death/growth/differentiation	Gga.3179.1.S2_at	brain abundant, membrane attached signal protein 1	8.359
Cell death/growth/differentiation	GgaAffx.12413.1.S1_at	RNA binding motif protein 38	6.574
Cell death/growth/differentiation	Gga.5164.1.S1_at	BCL2-related protein A1	5.638
Cell death/growth/differentiation	Gga.11726.1.S1_a_at	Growth arrest and DNA-damage-inducible, gamma	5.443
Cell death/growth/differentiation	GgaAffx.21663.1.S1_s_at	hepatoma-derived growth factor, related protein 3	5.272
Cell death/growth/differentiation	Gga.7539.1.S1_at	forkhead box L2	4.678
Cell death/growth/differentiation	Gga.4328.1.S1_at	insulin-like growth factor binding protein 7	4.173
Cell death/growth/differentiation	Gga.9179.1.S1_at	death-associated protein kinase 1	3.825
Cell death/growth/differentiation	GgaAffx.12808.1.S1_s_at	shisa homolog 5 (Xenopus laevis)	3.553
Cell death/growth/differentiation	Gga.4720.3.S1_a_at	coiled-coil domain containing 21	3.546
Cell death/growth/differentiation	Gga.7709.1.S1_at	death-associated protein kinase 1	3.509
Cell death/growth/differentiation	Gga.4176.1.S1_at	eukaryotic translation initiation factor 5A2	3.219
Cell death/growth/differentiation	Gga.2628.1.S1_s_at	cyclin D2	0.482
Cell death/growth/differentiation	Gga.1325.1.S1_at	MAD1 mitotic arrest deficient-like 1 (yeast)	0.453
Cell death/growth/differentiation	GgaAffx.4278.2.S1_s_at	transforming growth factor, beta receptor associated protein 1	0.448
Cell death/growth/differentiation	Gga.2554.1.A1_at	transforming growth factor, beta 3	0.399
Cell death/growth/differentiation	Gga.10930.1.S1_at	BCL2-like 13 (apoptosis facilitator)	0.396
Cell death/growth/differentiation	GgaAffx.10239.1.S1_at	optic atrophy 1 (autosomal dominant)	0.395
Cell death/growth/differentiation	GgaAffx.24127.1.S1_at	fibroblast growth factor receptor 1	0.372
Cell death/growth/differentiation	GgaAffx.25789.4.S1_s_at	fibroblast growth factor 1 (acidic)	0.283
Cell death/growth/differentiation	GgaAffx.4984.1.S1_at	cell division cycle 2, G1 to S and G2 to M	0.258
Cell death/growth/differentiation	GgaAffx.10104.2.S1_s_at	Gametocyte specific factor 1	0.245
Chaperonin/HSP	GgaAffx.4144.1.S1_s_at	DnaJ (Hsp40) homolog, subfamily A, member 4	0.491
Chaperonin/HSP	GgaAffx.5862.1.S1_at	t-complex 1	0.462
Chaperonin/HSP	GgaAffx.4681.1.S1_at	t-complex 11 (mouse)-like 2	0.391
Chaperonin/HSP	GgaAffx.193.1.S1_at	DnaJ (Hsp40) homolog, subfamily C, member 11	0.387
Chaperonin/HSP	GgaAffx.6430.1.S1_at	chaperonin containing TCP1, subunit 2 (beta)	0.379
Cytoskeletal components/regulatior	GgaAffx.21017.1.S1_s_at	caldesmon 1	29.979
Cytoskeletal components/regulatior	Gga.4091.1.S2_a_at	myosin, light chain kinase	23.408
Cytoskeletal components/regulatior	GgaAffx.21386.1.S1_s_at	caldesmon 1	16.044
Cytoskeletal components/regulatior	Gga.4988.2.S1_a_at	caldesmon 1	14.532
Cytoskeletal components/regulatior	Gga.1294.1.S1_at	myosin IF	10.163
Cytoskeletal components/regulatior	Gga.1009.1.S1_s_at	actin related protein 2/3 complex, subunit 1B, 41kDa	8.429
Cytoskeletal components/regulatior	Gga.2268.1.S1_at	centrosomal protein 170kDa	8.138
Cytoskeletal components/regulatior	GgaAffx.12091.1.S1_at	calponin 2	7.683
Cytoskeletal components/regulatior	Gga.13147.1.S1_at	Kinesin light chain 1	6.638
Cytoskeletal components/regulatior	Gga.1009.2.S1_s_at	actin related protein 2/3 complex, subunit 1B, 41kDa	6.556
Cytoskeletal components/regulatior	Gga.1350.1.S1_at	ARP3 actin-related protein 3 homolog B (yeast)	5.890
Cytoskeletal components/regulatior	Gga.11948.2.S1_a_at	Kinesin light chain 1	5.404
Cytoskeletal components/regulatior	Gga.4533.6.A1_at	Dynein, light chain, Tctex-type 1	4.778
Cytoskeletal components/regulatior	Gga.3908.1.S1_at	transgelin	4.369
Cytoskeletal components/regulatior	Gga.4661.2.S1_at	tubulin, alpha 1c	3.717
Cytoskeletal components/regulatior	Gga.1746.1.S1_s_at	similar to kinesin, putative	3.638
Cytoskeletal components/regulatior	Gga.10463.1.S1_s_at	cytoskeleton associated protein 5	3.501
Cytoskeletal components/regulatior	Gga.7794.1.S1_at	tubulin, beta 6	3.342
Cytoskeletal components/regulatior	Gga.8075.1.S1_at	Kinesin family member 5C	3.211
Cytoskeletal components/regulatior	Gga.16889.1.S1_at	myosin regulatory light chain interacting protein	3.056
Cytoskeletal components/regulatior	Gga.4425.1.S1_at	Myosin VI	0.488
Cytoskeletal components/regulatior	Gga.8635.1.S1_at	tubulin folding cofactor E	0.458
Cytoskeletal components/regulatior	GgaAffx.21775.1.S1_s_at	microtubule-associated protein 4	0.442
Cytoskeletal components/regulatior	GgaAffx.2648.1.S1_s_at	microtubule associated monoxygenase, calponin and LIM domain containing 3	0.437
Cytoskeletal components/regulatior	GgaAffx.2572.1.S1_at	dynein, cytoplasmic 1, light intermediate chain 1	0.420
Cytoskeletal components/regulatior	GgaAffx.10140.2.S1_s_at	kinesin family member 18A	0.393
Cytoskeletal components/regulatior	Gga.4830.1.S1_at	Actin, gamma 1	0.363
Cytoskeletal components/regulatior	Gga.11487.1.S1_s_at	centromere protein E, 312kDa	0.363
DNA replication/repair	Gga.18956.1.S1_s_at	polymerase (DNA directed), lambda	3.015
DNA replication/repair	Gga.18300.1.S1_at	excision repair cross-complementing rodent repair deficiency, complementation group 5	0.498
DNA replication/repair	Gga.7505.1.S1_at	REX2, RNA exonuclease 2 homolog (S. cerevisiae)	0.491
DNA replication/repair	Gga.2148.4.S1_a_at	exonuclease NEF-sp	0.484
DNA replication/repair	Gga.10998.1.S1_s_at	RAD18 homolog (S. cerevisiae)	0.476
DNA replication/repair	GgaAffx.24832.2.S1_s_at	breast cancer 1, early onset	0.475
DNA replication/repair	GgaAffx.26038.1.S1_at	polymerase (DNA-directed), delta 3, accessory subunit	0.465
DNA replication/repair	Gga.4315.2.S1_a_at	replication protein A2, 32kDa	0.455

DNA replication/repair	Gga.710.6.S1_a_at	polymerase (DNA directed), gamma	0.450
DNA replication/repair	GgaAffx.4442.3.S1_s_at	replication factor C (activator 1) 3, 38kDa	0.440
DNA replication/repair	Gga.11871.1.S1_at	minichromosome maintenance complex component 2	0.435
DNA replication/repair	Gga.12043.2.S1_a_at	polymerase (DNA-directed), delta interacting protein 3	0.428
DNA replication/repair	GgaAffx.7758.2.S1_s_at	DNA2 DNA replication helicase 2-like (yeast)	0.408
DNA replication/repair	GgaAffx.21825.1.S1_s_at	RAD51-like 1 (S. cerevisiae)	0.398
DNA replication/repair	GgaAffx.25679.1.S1_s_at	poly (ADP-ribose) polymerase family, member 11	0.380
Extracellular matrix	Gga.12800.1.S1_at	matrix metallopeptidase 3 (stromelysin 1, progelatinase)	67.335
Extracellular matrix	Gga.3994.3.S1_x_at	Fibronectin 1	23.201
Extracellular matrix	Gga.9293.1.S1_at	Fibronectin 1	21.558
Extracellular matrix	Gga.4078.1.S1_at	lumican	21.079
Extracellular matrix	Gga.701.1.S1_s_at	leukocyte ribonuclease A-1 /// leukocyte ribonuclease A-2	20.283
Extracellular matrix	Gga.4851.1.S1_at	collagen, type III, alpha 1	19.577
Extracellular matrix	GgaAffx.21771.1.S1_at	collagen, type VI, alpha 1	16.921
Extracellular matrix	Gga.2073.1.S1_at	Alpha-1 collagen (I)	16.450
Extracellular matrix	Gga.4941.1.S1_at	cadherin 11, type 2, OB-cadherin (osteoblast)	15.052
Extracellular matrix	Gga.4128.1.S2_at	tensin 1	14.084
Extracellular matrix	Gga.3199.1.S1_at	matrix metallopeptidase 2 (gelatinase A, 72kDa gelatinase, 72kDa type IV collagenase)	13.372
Extracellular matrix	Gga.20018.1.S1_at	thrombomucin	12.438
Extracellular matrix	Gga.4851.1.A1_at	collagen, type III, alpha 1	11.954
Extracellular matrix	Gga.5255.1.S1_at	EGF-containing fibulin-like extracellular matrix protein 1	11.838
Extracellular matrix	Gga.12928.1.S1_at	vascular cell adhesion molecule 1	11.757
Extracellular matrix	Gga.540.1.S1_at	matrix Gla protein	11.638
Extracellular matrix	GgaAffx.11018.1.S1_s_at	matrix metallopeptidase 1 (interstitial collagenase)	11.544
Extracellular matrix	Gga.5171.1.S1_at	cadherin 5, type 2, VE-cadherin (vascular epithelium)	10.552
Extracellular matrix	Gga.1099.1.S1_at	annexin A6	10.396
Extracellular matrix	Gga.4999.2.S1_at	integrin, alpha 4 (antigen CD49D, alpha 4 subunit of VLA-4 receptor)	10.191
Extracellular matrix	Gga.4884.1.S1_at	annexin A1	9.299
Extracellular matrix	Gga.3104.1.S1_at	collagen, type IV, alpha 2	9.177
Extracellular matrix	GgaAffx.6053.1.S1_at	collagen, type I, alpha 2	7.860
Extracellular matrix	Gga.4507.1.S1_at	integrin, beta 2 (complement component 3 receptor 3 and 4 subunit)	7.479
Extracellular matrix	GgaAffx.5654.1.S1_at	integrin, alpha 4 (antigen CD49D, alpha 4 subunit of VLA-4 receptor)	7.224
Extracellular matrix	Gga.12039.1.S1_a_at	Matrix-remodelling associated 5	6.951
Extracellular matrix	GgaAffx.25534.1.S1_s_at	collagen, type III, alpha 1	6.917
Extracellular matrix	GgaAffx.25192.2.S1_s_at	matrix metallopeptidase 1 (interstitial collagenase)	6.677
Extracellular matrix	Gga.9346.3.S1_a_at	vimentin	6.418
Extracellular matrix	Gga.19452.1.S1_at	CD44 molecule (Indian blood group)	6.208
Extracellular matrix	Gga.1168.1.S1_at	annexin A5	6.099
Extracellular matrix	Gga.2679.1.S1_at	fibulin 1	6.062
Extracellular matrix	Gga.5776.1.S1_at	CD34 molecule	5.897
Extracellular matrix	GgaAffx.21790.1.S1_s_at	gap junction protein, alpha 1, 43kDa	5.744
Extracellular matrix	Gga.4051.1.S1_at	connective tissue growth factor	5.690
Extracellular matrix	GgaAffx.11429.1.S1_s_at	matrix metallopeptidase 7 (matrilysin, uterine)	5.654
Extracellular matrix	Gga.6223.1.S1_at	ADAM metallopeptidase domain 33	5.243
Extracellular matrix	Gga.4257.1.S1_at	collagen, type VI, alpha 2	5.106
Extracellular matrix	Gga.566.1.S1_at	integrin, alpha 1	4.793
Extracellular matrix	Gga.13442.1.S2_s_at	radixin	4.708
Extracellular matrix	Gga.3572.1.S1_at	CD44 molecule (Indian blood group)	4.619
Extracellular matrix	Gga.2965.1.S1_at	TIMP metallopeptidase inhibitor 2	4.032
Extracellular matrix	GgaAffx.22999.1.S1_s_at	collagen, type I, alpha 2	3.892
Extracellular matrix	Gga.13442.1.S1_at	radixin	3.637
Extracellular matrix	Gga.1776.1.S1_at	nidogen 1	3.562
Extracellular matrix	Gga.3572.1.S2_s_at	CD44 molecule (Indian blood group)	3.164
Extracellular matrix	Gga.888.1.S1_at	collagen, type VI, alpha 3	3.103
Extracellular matrix	Gga.198.1.S1_at	matrix metallopeptidase 9 (gelatinase B, 92kDa gelatinase, 92kDa type IV collagenase)	3.028
Extracellular matrix	GgaAffx.26220.2.S1_s_at	integrin beta 1 binding protein 3	0.480
Extracellular matrix	GgaAffx.6391.2.S1_s_at	integrator complex subunit 2	0.475
Extracellular matrix	GgaAffx.22425.1.S1_s_at	collagen, type XX, alpha 1	0.394
Extracellular matrix	GgaAffx.5541.1.S1_at	Discoidin, CUB and LCCL domain containing 1	0.356
Gene expression/regulation	Gga.3548.1.S1_at	zinc finger E-box binding homeobox 1	13.018
Gene expression/regulation	Gga.2551.2.S1_s_at	lactotransferrin	11.137
Gene expression/regulation	Gga.1313.1.S1_s_at	spleen focus forming virus (SFFV) proviral integration oncogene spi1	10.008
Gene expression/regulation	GgaAffx.20792.1.S1_s_at	zinc finger E-box binding homeobox 1	8.993
Gene expression/regulation	GgaAffx.12062.1.S1_s_at	Friend leukemia virus integration 1	8.875
Gene expression/regulation	GgaAffx.3506.1.S1_at	ecotropic viral integration site 2B	7.990
Gene expression/regulation	Gga.4285.1.S1_at	CCAAT/enhancer binding protein (C/EBP), beta	7.344
Gene expression/regulation	Gga.6066.1.S1_at	transcription factor 7 (T-cell specific, HMG-box)	7.083
Gene expression/regulation	Gga.1378.1.S1_s_at	histone deacetylase 9	5.821
Gene expression/regulation	Gga.3731.2.S1_a_at	gammaFBP-A	5.230

Gene expression/regulation	Gga.6375.1.S1_at	basic helix-loop-helix domain containing, class B, 8	5.205
Gene expression/regulation	Gga.4919.1.S2_at	CUG triplet repeat, RNA binding protein 2 /// hypothetical protein LOC769811	5.086
Gene expression/regulation	Gga.1108.1.S1_s_at	limb bud and heart development homolog (mouse)	4.534
Gene expression/regulation	Gga.4954.2.S1_a_at	TNFAIP3 interacting protein 1	4.510
Gene expression/regulation	Gga.4371.1.S1_s_at	putative homeodomain transcription factor 2	4.202
Gene expression/regulation	Gga.578.1.S1_at	IKAROS family zinc finger 1 (Ikaros)	4.108
Gene expression/regulation	GgaAffx.21436.1.S1_s_at	basic leucine zipper transcription factor, ATF-like	3.617
Gene expression/regulation	Gga.49.1.S1_at	chromobox homolog 4 (Pc class homolog, Drosophila)	3.394
Gene expression/regulation	GgaAffx.24709.1.S1_s_at	microphthalmia-associated transcription factor	0.498
Gene expression/regulation	Gga.16862.1.S1_at	TATA element modulatory factor 1	0.492
Gene expression/regulation	GgaAffx.2752.1.S1_s_at	MAX interactor 1	0.491
Gene expression/regulation	GgaAffx.4023.1.S1_at	REV3-like, catalytic subunit of DNA polymerase zeta (yeast)	0.490
Gene expression/regulation	GgaAffx.8903.3.S1_at	nuclear receptor subfamily 6, group A, member 1	0.487
Gene expression/regulation	GgaAffx.21044.1.S1_s_at	pseudouridylate synthase 7 homolog (S. cerevisiae)	0.482
Gene expression/regulation	GgaAffx.2077.1.S1_s_at	v-myc myelocytomatosis viral oncogene homolog (avian)	0.480
Gene expression/regulation	GgaAffx.25677.1.S1_s_at	Sin3A-associated protein, 130kDa	0.475
Gene expression/regulation	GgaAffx.23089.1.S1_at	general transcription factor IIA, 2, 12kDa	0.468
Gene expression/regulation	GgaAffx.25648.2.S1_s_at	WD repeat domain 36	0.463
Gene expression/regulation	GgaAffx.26668.2.S1_s_at	nuclear transcription factor Y, gamma	0.462
Gene expression/regulation	Gga.2475.1.S1_at	chromatin modifying protein 6	0.458
Gene expression/regulation	Gga.10895.1.S1_s_at	threonyl-tRNA synthetase	0.451
Gene expression/regulation	GgaAffx.4249.1.S1_x_at	CTD (carboxy-terminal domain, RNA polymerase II, polypeptide A) phosphatase, subunit 1	0.448
Gene expression/regulation	Gga.4830.1.S2_at	protein inhibitor of activated STAT, 2	0.446
Gene expression/regulation	Gga.5522.2.S1_a_at	activating signal cointegrator 1 complex subunit 3-like 1	0.440
Gene expression/regulation	GgaAffx.2639.1.S1_at	adenosine deaminase, tRNA-specific 1	0.437
Gene expression/regulation	GgaAffx.4639.1.S1_at	splicing factor 3b, subunit 4, 49kDa	0.435
Gene expression/regulation	Gga.6487.1.S1_at	similar to nuclear cap binding protein subunit 2	0.434
Gene expression/regulation	GgaAffx.9629.1.S1_at	chromatin licensing and DNA replication factor 1	0.427
Gene expression/regulation	GgaAffx.20192.1.S1_s_at	ribosomal protein S11	0.417
Gene expression/regulation	Gga.2087.1.S1_s_at	RNA polymerase 1-3	0.415
Gene expression/regulation	GgaAffx.8122.1.S1_at	nucleolar protein 5A (56kDa with KKE/D repeat)	0.415
Gene expression/regulation	Gga.4010.1.S1_s_at	integrator complex subunit 2	0.415
Gene expression/regulation	Gga.11672.1.S1_at	PRP4 pre-mRNA processing factor 4 homolog (yeast)	0.413
Gene expression/regulation	GgaAffx.193.2.S1_s_at	integrator complex subunit 10	0.412
Gene expression/regulation	Gga.17691.1.S1_at	activating transcription factor 4 (tax-responsive enhancer element B67)	0.408
Gene expression/regulation	GgaAffx.1279.2.S1_s_at	caudal type homeobox 2	0.383
Gene expression/regulation	GgaAffx.3047.2.S1_s_at	ribosomal protein L36	0.342
Gene expression/regulation	GgaAffx.1433.3.S1_s_at	similar to general transcription factor IIIC, polypeptide 1, alpha 220kDa	0.339
Gene expression/regulation	GgaAffx.10949.1.S1_s_at	ribosomal protein L38	0.333
Gene expression/regulation	GgaAffx.3310.1.S1_s_at	exonuclease domain containing 1	0.319
Gene expression/regulation	Gga.19462.1.S1_s_at	transcriptional adaptor 2 (ADA2 homolog, yeast)-like	0.316
Gene expression/regulation	Gga.12360.1.S1_at	Splicing factor, arginine/serine-rich 3	0.315
General metabolism	Gga.17674.1.S1_at	Biliverdin reductase A	29.576
General metabolism	GgaAffx.23872.1.S1_at	Biliverdin reductase A	14.424
General metabolism	Gga.739.1.S1_at	quiescence-specific protein	11.988
General metabolism	GgaAffx.7420.1.S1_at	phospholipase D family, member 4	8.185
General metabolism	GgaAffx.26353.1.S1_s_at	GTPase, IMAP family member 8	7.820
General metabolism	Gga.11830.1.S1_at	deoxyribonuclease I-like 3	7.752
General metabolism	Gga.1177.1.S1_at	ELOVL family member 5, elongation of long chain fatty acids (FEN1/Elo2, SUR4/Elo3-like)	7.159
General metabolism	Gga.6017.1.S1_s_at	Biliverdin reductase A	7.089
General metabolism	Gga.6236.1.S1_at	switch-associated protein 70	7.025
General metabolism	Gga.7765.1.S1_at	carbohydrate (N-acetylglucosamine-6-O) sulfotransferase 2	6.813
General metabolism	Gga.10928.1.S1_at	cholesterol 25-hydroxylase	5.922
General metabolism	GgaAffx.10393.1.S1_at	cytochrome b-245, beta polypeptide (chronic granulomatous disease)	5.857
General metabolism	Gga.13365.1.S1_at	1-acylglycerol-3-phosphate O-acyltransferase 4 (lysophosphatidic acid acyltransferase, delta)	5.713
General metabolism	Gga.14925.1.S1_at	mannosyl (alpha-1,3-)-glycoprotein beta-1,4-N-acetylglucosaminyltransferase, isozyme A	5.460
General metabolism	Gga.3807.1.S2_s_at	aldehyde dehydrogenase 1 family, member A3	5.432
General metabolism	Gga.4401.1.S1_a_at	prostaglandin-endoperoxide synthase 2 (prostaglandin G/H synthase and cyclooxygenase)	5.150
General metabolism	Gga.14426.1.S1_at	lipase A, lysosomal acid, cholesterol esterase (Wolman disease)	4.885
General metabolism	Gga.7148.2.A1_at	phospholipase C, gamma 2 (phosphatidylinositol-specific)	4.612
General metabolism	Gga.6612.2.A1_at	NK-lysin	4.252
General metabolism	GgaAffx.12860.1.S1_at	thioredoxin domain containing 5 (endoplasmic reticulum)	4.149
General metabolism	Gga.12233.1.S1_at	N-acylsphingosine amidohydrolase (acid ceramidase)-like	4.123
General metabolism	Gga.7640.2.S1_at	Acyl-CoA synthetase long-chain family member 4	4.017
General metabolism	GgaAffx.3425.1.S1_at	phospholipase C, gamma 2 (phosphatidylinositol-specific)	4.003

General metabolism	Gga.9879.1.S1_s_at	lipase A, lysosomal acid, cholesterol esterase (Wolman disease)	3.893
General metabolism	Gga.3323.1.S1_s_at	fatty acid binding protein 5	3.892
General metabolism	GgaAffx.1032.1.S1_s_at	inositol polyphosphate-5-phosphatase, 145kDa	3.863
General metabolism	GgaAffx.12708.1.S1_s_at	similar to ornithine aminotransferase (gyrate atrophy) /// ornithine aminotransferase	3.692
General metabolism	Gga.1161.1.S1_at	succinate dehydrogenase complex, subunit D, integral membrane protein	3.504
General metabolism	GgaAffx.22378.1.S1_s_at	phospholipase C, gamma 2 (phosphatidylinositol-specific)	3.219
General metabolism	Gga.7148.1.S1_at	phospholipase C, gamma 2 (phosphatidylinositol-specific)	3.218
General metabolism	Gga.1758.1.S1_at	Arachidonate 5-lipoxygenase-activating protein	3.208
General metabolism	Gga.7640.1.S1_a_at	Acyl-CoA synthetase long-chain family member 4	3.201
General metabolism	Gga.593.1.S1_s_at	ADP-ribosyltransferase /// ADP-ribosyltransferase	3.188
General metabolism	GgaAffx.4938.1.S1_at	phosphatidylinositol-5-phosphate 4-kinase, type II, alpha	3.020
General metabolism	Gga.10824.1.S1_s_at	N-acetylglucosaminyltransferase VI	0.490
General metabolism	Gga.7294.1.S1_at	choline phosphotransferase 1	0.490
General metabolism	Gga.9508.1.S1_at	glutamine-fructose-6-phosphate transaminase 1	0.488
General metabolism	GgaAffx.3796.1.S1_s_at	phosphorylase kinase, beta	0.486
General metabolism	GgaAffx.22454.1.S1_s_at	arachidonate 5-lipoxygenase	0.484
General metabolism	GgaAffx.12570.1.S1_s_at	Oxysterol binding protein-like 11	0.483
General metabolism	GgaAffx.21140.1.S1_s_at	NADH dehydrogenase (ubiquinone) 1 alpha subcomplex, 2, 8kDa	0.476
General metabolism	GgaAffx.23988.2.S1_s_at	gallinacin 1	0.475
General metabolism	Gga.2908.1.S1_at	asparagine-linked glycosylation 11 homolog (S. cerevisiae, alpha-1,2-mannosyltransferase)	0.472
General metabolism	GgaAffx.24513.9.S1_s_at	asparagine-linked glycosylation 8 homolog (S. cerevisiae, alpha-1,3-glucosyltransferase)	0.470
General metabolism	GgaAffx.26615.2.S1_s_at	Similar to dermatan 4-sulfotransferase-1	0.466
General metabolism	Gga.11674.1.S1_at	methyltransferase like 8	0.442
General metabolism	GgaAffx.24884.1.S1_s_at	dUTP pyrophosphatase	0.438
General metabolism	Gga.5263.3.S1_s_at	phospholipase C-like 2	0.438
General metabolism	GgaAffx.882.2.S1_s_at	acyl-CoA synthetase long-chain family member 5	0.432
General metabolism	Gga.8991.1.S1_at	glutathione S-transferase theta 1	0.424
General metabolism	Gga.17281.1.S1_at	UDP-Gal:betaGlcNAc beta 1,4- galactosyltransferase, polypeptide 1	0.417
General metabolism	Gga.11447.1.S1_a_at	K(lysine) acetyltransferase 2A	0.408
General metabolism	GgaAffx.23111.1.S1_s_at	uridine phosphorylase 2	0.397
General metabolism	GgaAffx.25247.1.S1_at	UDP-glucuronate decarboxylase 1	0.395
General metabolism	Gga.17284.1.S1_at	serine racemase	0.375
General metabolism	GgaAffx.24100.1.S1_s_at	steroid 5 alpha-reductase 3	0.365
General metabolism	GgaAffx.26520.1.S1_s_at	UDP-glucose pyrophosphorylase 2	0.364
General metabolism	Gga.12113.1.A1_at	methyltransferase like 14	0.359
General metabolism	GgaAffx.10350.1.S1_at	5'-nucleotidase, cytosolic III-like	0.356
General metabolism	Gga.9596.1.S1_at	cytochrome P450, family 39, subfamily A, polypeptide 1	0.351
General metabolism	GgaAffx.21089.1.S1_s_at	STT3, subunit of the oligosaccharyltransferase complex, homolog B (S. cerevisiae)	0.349
General metabolism	GgaAffx.5994.1.S1_at	methyltransferase like 10	0.304
Immunity/defense	Gga.12186.1.S1_at	lymphatic vessel endothelial hyaluronan receptor 1	32.611
Immunity/defense	Gga.1020.1.S1_at	chemokine (C-C motif) ligand 17	29.428
Immunity/defense	Gga.701.1.S1_at	leukocyte ribonuclease A-2	18.493
Immunity/defense	Gga.9873.1.S1_at	complement component 1, q subcomponent, C chain	16.683
Immunity/defense	Gga.5396.1.S1_at	complement component 1, q subcomponent, B chain	14.962
Immunity/defense	Gga.7298.1.S1_at	complement component 1, q subcomponent, A chain	14.601
Immunity/defense	Gga.11955.1.S1_at	Immunoglobulin superfamily, member 6	14.396
Immunity/defense	Gga.9513.1.S2_at	Chemokine (C-X-C motif) ligand 12 (stromal cell-derived factor 1)	13.883
Immunity/defense	Gga.9133.1.S1_at	Chemokine ah221	12.744
Immunity/defense	Gga.4374.1.S1_a_at	interferon regulatory factor 4	12.149
Immunity/defense	Gga.3676.1.S1_at	B cell RAG associated protein	12.106
Immunity/defense	Gga.4681.2.S1_s_at	dedicator of cytokinesis 10	11.854
Immunity/defense	GgaAffx.2960.1.S1_s_at	complement component 1, q subcomponent, B chain	11.824
Immunity/defense	Gga.703.1.S1_at	leukocyte ribonuclease A-1	11.803
Immunity/defense	Gga.916.1.S2_at	interferon, gamma	11.653
Immunity/defense	Gga.1307.1.S1_s_at	interleukin 16 (lymphocyte chemoattractant factor)	11.579
Immunity/defense	Gga.2467.1.S1_at	immunoglobulin J polypeptide, linker protein for immunoglobulin alpha and mu polypeptides	11.272
Immunity/defense	Gga.17679.1.S1_s_at	immunoglobulin-like receptor CHIR-B5 /// immunoglobulin-like receptor CHIR-B6	11.200
Immunity/defense	Gga.4110.4.S1_x_at	immunoglobulin lambda-like polypeptide 1	11.067
Immunity/defense	Gga.4110.10.S1_x_at	immunoglobulin lambda-like polypeptide 1	10.715
Immunity/defense	Gga.5743.1.S1_at	lymphocyte antigen 96	10.485
Immunity/defense	GgaAffx.151.1.S1_s_at	CD79b molecule, immunoglobulin-associated beta	9.962
Immunity/defense	Gga.4110.11.S1_x_at	immunoglobulin lambda-like polypeptide 1	9.867
Immunity/defense	GgaAffx.11201.1.S1_s_at	toll-like receptor 6	9.819

Immunity/defense	Gga.3145.1.S1_x_at	similar to Moesin (Membrane-organizing extension spike protein)	9.804
Immunity/defense	Gga.1311.5.S1_s_at	Similar to T-cell receptor V beta chain	9.765
Immunity/defense	GgaAffx.10617.2.S1_s_at	toll-like receptor 7	9.749
Immunity/defense	GgaAffx.5458.1.S1_at	cytotoxic T-lymphocyte-associated protein 4	9.664
Immunity/defense	Gga.6032.1.S1_at	dedicator of cytokinesis 8	9.560
Immunity/defense	GgaAffx.25386.1.S1_at	dedicator of cytokinesis 2	9.265
Immunity/defense	GgaAffx.21915.1.S1_at	interferon-induced protein with tetratricopeptide repeats 5	9.081
Immunity/defense	GgaAffx.2421.1.S1_s_at	T-cell immunoglobulin and mucin domain containing 4	9.060
Immunity/defense	Gga.723.1.S1_a_at	CD28 molecule	9.052
Immunity/defense	Gga.4491.1.S1_at	Lymphocyte-specific protein 1	9.021
Immunity/defense	Gga.21.1.S1_a_at	interleukin 2 receptor, gamma (severe combined immunodeficiency)	8.796
Immunity/defense	Gga.17647.1.S1_at	neutrophil cytosolic factor 2 (65kDa, chronic granulomatous disease, autosomal 2)	8.778
Immunity/defense	Gga.690.1.S1_at	lymphocyte antigen 86	8.760
Immunity/defense	GgaAffx.12658.1.S1_at	neutrophil cytosolic factor 4, 40kDa	8.655
Immunity/defense	GgaAffx.26062.1.S1_s_at	immunoglobulin-like receptor CHIR-B3 /// similar to immunoglobulin-like receptor CHIR-B3	8.641
Immunity/defense	GgaAffx.12842.1.S1_s_at	immunoresponsive 1 homolog (mouse)	8.616
Immunity/defense	Gga.445.1.S1_at	similar to T cell receptor alpha	8.522
Immunity/defense	Gga.445.2.S1_a_at	similar to T cell receptor alpha	8.396
Immunity/defense	Gga.9675.1.S1_at	platelet derived growth factor D	8.338
Immunity/defense	Gga.3145.3.S1_a_at	similar to Moesin (Membrane-organizing extension spike protein)	8.166
Immunity/defense	Gga.236.1.S1_at	lymphocyte cytosolic protein 2 (SH2 domain containing leukocyte protein of 76kDa)	7.935
Immunity/defense	Gga.19049.1.S1_at	interleukin 18 (interferon-gamma-inducing factor)	7.852
Immunity/defense	GgaAffx.25386.2.S1_s_at	dedicator of cytokinesis 2	7.785
Immunity/defense	Gga.3366.1.S1_at	interleukin 20 receptor, alpha	7.766
Immunity/defense	Gga.11522.1.S1_x_at	similar to immunoglobulin-like receptor CHIR-AB1	7.748
Immunity/defense	Gga.4110.4.S1_a_at	immunoglobulin lambda-like polypeptide 1	7.729
Immunity/defense	GgaAffx.9789.1.S1_at	T cell receptor associated transmembrane adaptor 1	7.620
Immunity/defense	Gga.217.1.S1_x_at	MHC class II antigen B-F minor heavy chain	7.599
Immunity/defense	Gga.4110.12.S1_a_at	immunoglobulin lambda-like polypeptide 1	7.546
Immunity/defense	Gga.14571.1.S1_s_at	Hermansky-Pudlak syndrome 5	7.475
Immunity/defense	Gga.3145.2.S1_a_at	similar to Moesin (Membrane-organizing extension spike protein)	7.466
Immunity/defense	Gga.11522.1.S1_at	similar to immunoglobulin-like receptor CHIR-AB1	7.247
Immunity/defense	GgaAffx.10876.2.S1_s_at	lymphocyte cytosolic protein 1 (L-plastin)	7.125
Immunity/defense	GgaAffx.24185.1.S1_at	interleukin 7 receptor	7.054
Immunity/defense	Gga.1220.1.A1_at	pre-B lymphocyte gene 3	6.815
Immunity/defense	Gga.6332.1.S1_at	similar to complement regulatory soluble protein	6.733
Immunity/defense	Gga.1311.3.S1_at	similar to T-cell receptor beta	6.695
Immunity/defense	GgaAffx.11727.1.S1_at	lymphocyte cytosolic protein 1 (L-plastin)	6.591
Immunity/defense	Gga.3380.1.S1_at	T cell receptor alpha locus	6.418
Immunity/defense	Gga.13336.2.S1_x_at	MHC class II antigen B-F minor heavy chain	6.280
Immunity/defense	GgaAffx.12851.1.S1_s_at	complement component 3a receptor 1	6.058
Immunity/defense	Gga.542.1.S1_at	lymphotactin	6.045
Immunity/defense	GgaAffx.21398.1.S1_at	dedicator of cytokinesis 10	5.998
Immunity/defense	Gga.4491.1.S1_s_at	Lymphocyte-specific protein 1	5.811
Immunity/defense	GgaAffx.24298.1.S1_s_at	interleukin 20 receptor, alpha	5.807
Immunity/defense	Gga.4964.5.S1_a_at	similar to T cell receptor gamma /// similar to T-cell receptor gamma Vg3-Jg3	5.646
Immunity/defense	Gga.7064.1.S1_at	immunoglobulin superfamily, member 1	5.593
Immunity/defense	GgaAffx.8515.1.S1_at	interleukin 7 receptor	5.554
Immunity/defense	Gga.4964.17.S1_x_at	similar to T-cell receptor gamma Vg3-Jg3	5.493
Immunity/defense	Gga.4964.3.S1_x_at	similar to T-cell receptor gamma Vg3-Jg3	5.489
Immunity/defense	Gga.4964.11.S1_a_at	similar to T-cell receptor gamma V2.6a /// similar to T-cell receptor gamma Vg3-Jg3	5.488
Immunity/defense	Gga.4964.2.S1_a_at	similar to T cell receptor gamma /// similar to T-cell receptor gamma Vg3-Jg3	5.468
Immunity/defense	Gga.11252.1.S1_at	chemokine (C-C motif) ligand 19	5.454
Immunity/defense	GgaAffx.23145.3.S1_s_at	dedicator of cytokinesis 8	5.299
Immunity/defense	Gga.16655.1.S1_at	histocompatibility (minor) HA-1	5.279
Immunity/defense	Gga.4964.54.A1_x_at	Similar to T-cell receptor gamma chain Vg3-Jg3	5.271
Immunity/defense	Gga.296.1.S1_at	chemokine (C-C motif) ligand 1	5.250
Immunity/defense	Gga.178.1.S1_s_at	guanylate binding protein	5.180
Immunity/defense	Gga.4964.22.S1_x_at	Similar to T-cell receptor gamma chain Vg1-Jg2	5.162
Immunity/defense	GgaAffx.12157.1.S1_at	similar to immunoglobulin-like receptor CHIR-B2 precursor	5.131
Immunity/defense	Gga.65.1.S1_a_at	similar to T-cell receptor gamma Vg3-Jg3	5.120
Immunity/defense	Gga.16589.1.S1_at	Lymphocyte-specific protein 1	5.102
Immunity/defense	GgaAffx.11648.1.S1_at	B-cell CLL/lymphoma 6 (zinc finger protein 51)	5.042
Immunity/defense	Gga.4964.1.S2_at	similar to T cell receptor gamma /// similar to T-cell receptor gamma Vg3-Jg3	5.031

Immunity/defense	Gga.19.1.S1_at	interleukin 1, beta	5.023
Immunity/defense	GgaAffx.1625.1.S1_s_at	complement factor H	4.910
Immunity/defense	Gga.4964.1.S1_x_at	similar to T-cell receptor gamma Vg3-Jg3	4.909
Immunity/defense	GgaAffx.7251.1.S1_at	chemokine (C-C motif) receptor 6	4.853
Immunity/defense	GgaAffx.5454.1.S1_at	inducible T-cell co-stimulator	4.850
Immunity/defense	Gga.6529.1.S1_at	Fc fragment of IgE, high affinity I, receptor for; gamma polypeptide	4.849
Immunity/defense	GgaAffx.20498.1.S1_s_at	monocyte to macrophage differentiation-associated	4.758
Immunity/defense	Gga.4964.4.S1_x_at	similar to T cell receptor gamma /// similar to T-cell receptor gamma chain Vg1-Jg2	4.613
Immunity/defense	Gga.4414.1.S1_s_at	CD74 molecule, major histocompatibility complex, class II invariant chain	4.574
Immunity/defense	GgaAffx.26385.1.S1_s_at	dedicator of cytokinesis 10	4.569
Immunity/defense	Gga.18469.1.S1_s_at	dedicator of cytokinesis 8	4.459
Immunity/defense	Gga.276.1.S1_at	early B-cell factor 1	4.410
Immunity/defense	Gga.65.1.S1_x_at	similar to T-cell receptor gamma Vg3-Jg3	4.396
Immunity/defense	Gga.6492.1.S1_at	similar to chemokine CXCL13/BCA-1	4.390
Immunity/defense	Gga.3027.1.S1_at	CD40 ligand (TNF superfamily, member 5, hyper-IgM syndrome)	4.359
Immunity/defense	Gga.4964.11.S1_x_at	similar to T-cell receptor gamma V2.6a /// similar to T-cell receptor gamma Vg3-Jg3	4.265
Immunity/defense	Gga.2734.1.S2_at	activated leukocyte cell adhesion molecule	4.249
Immunity/defense	Gga.15811.2.S1_at	cytokine-like nuclear factor n-pac	4.236
Immunity/defense	Gga.4964.53.S1_x_at	similar to T-cell receptor gamma Vg3-Jg3	4.206
Immunity/defense	Gga.4964.54.S1_x_at	similar to T-cell receptor gamma chain Vg3-Jg3 /// similar to T-cell receptor gamma Vg3-Jg3	4.099
Immunity/defense	GgaAffx.7943.1.S1_s_at	neutrophil cytosolic factor 4, 40kDa	4.086
Immunity/defense	Gga.4964.20.S1_x_at	similar to T-cell receptor gamma Vg3-Jg3 /// similar to T-cell receptor gamma chain Vg3-Jg1	4.051
Immunity/defense	Gga.9563.1.S1_at	chemokine (C-C motif) ligand 4 /// chemokine (C-C motif) ligand 5	4.025
Immunity/defense	GgaAffx.7589.1.S1_at	chemokine (C-X3-C motif) receptor 1	4.018
Immunity/defense	Gga.12516.1.S1_s_at	interleukin 20 receptor, alpha	3.896
Immunity/defense	Gga.4964.25.S1_x_at	similar to T-cell receptor gamma chain Vg3-Jg3 /// similar to T-cell receptor gamma Vg3-Jg3	3.794
Immunity/defense	Gga.13336.3.S1_a_at	MHC class II antigen B-F minor heavy chain /// MHC class II beta chain	3.775
Immunity/defense	Gga.575.1.S1_at	CD8a molecule	3.768
Immunity/defense	Gga.4185.1.S2_at	Interferon (alpha, beta and omega) receptor 1	3.730
Immunity/defense	Gga.2510.1.S1_at	complement component 3	3.701
Immunity/defense	Gga.13336.5.S1_x_at	MHC class II antigen B-F minor heavy chain /// MHC class II beta chain	3.682
Immunity/defense	Gga.10606.1.S1_at	suppressor of cytokine signaling 1	3.610
Immunity/defense	Gga.13336.3.S1_x_at	MHC class II antigen B-F minor heavy chain /// MHC class II beta chain	3.600
Immunity/defense	GgaAffx.8901.1.S1_at	lymphoid-restricted membrane protein	3.578
Immunity/defense	Gga.16267.1.S1_s_at	similar to MHC Rfp-Y class I alpha chain /// similar to MHC Rfp-Y class I alpha chain	3.476
Immunity/defense	Gga.4491.1.A1_at	Lymphocyte-specific protein 1	3.454
Immunity/defense	Gga.1328.2.A1_at	Beta-2-microglobulin	3.439
Immunity/defense	Gga.4995.1.A1_at	Similar to T-cell receptor gamma chain Vg2-Jg3	3.401
Immunity/defense	GgaAffx.11658.1.S1_s_at	neutrophil cytosolic factor 1	3.382
Immunity/defense	Gga.4964.6.S1_x_at	similar to T cell receptor gamma /// similar to T-cell receptor gamma Vg3-Jg3	3.380
Immunity/defense	Gga.2529.1.S1_a_at	interleukin 1 receptor-like 1	3.313
Immunity/defense	Gga.7100.1.S1_at	similar to macrophage inflammatory protein-2	3.199
Immunity/defense	Gga.15027.1.S1_at	Signaling lymphocytic activation molecule family member 1	3.187
Immunity/defense	Gga.916.1.S1_at	interferon, gamma	3.104
Immunity/defense	Gga.4973.3.S1_x_at	MHC class I antigen B-F minor heavy chain	3.085
Immunity/defense	GgaAffx.21845.2.S1_s_at	CD74 molecule, major histocompatibility complex, class II invariant chain	3.070
Immunity/defense	GgaAffx.12703.1.S1_at	similar to Toll-like receptor 21	3.063
Immunity/defense	GgaAffx.12160.1.S1_s_at	cytokine receptor-like factor 3	3.041
Immunity/defense	Gga.7057.1.S1_at	caspase recruitment domain family, member 11	3.036
Immunity/defense	GgaAffx.26702.1.S1_s_at	similar to dedicator of cytokinesis 11	3.026
Immunity/defense	Gga.9732.1.S1_at	inter-alpha (globulin) inhibitor H5	3.022
Immunity/defense	Gga.1045.1.S1_at	similar to dedicator of cytokinesis 11	3.002
Immunity/defense	Gga.8178.1.S1_at	interferon stimulated exonuclease gene 20kDa-like 2	0.477
Immunity/defense	Gga.5922.2.S1_a_at	platelet-activating factor acetylhydrolase 2, 40kDa	0.449
Immunity/defense	GgaAffx.26288.1.S1_s_at	complement component 1, r subcomponent	0.447
Immunity/defense	Gga.11984.1.S1_at	dedicator of cytokinesis 5	0.399
Immunity/defense	GgaAffx.20791.1.S1_at	interleukin 28B (interferon, lambda 3)	0.347
Mitochondrial protein	Gga.5224.1.S1_at	Carbonic anhydrase VB, mitochondrial	3.017
Mitochondrial protein	GgaAffx.12907.1.S1_at	mitochondrial ribosomal protein L18	0.495
Mitochondrial protein	GgaAffx.10169.1.S1_at	leucyl-tRNA synthetase 2, mitochondrial	0.493

Mitochondrial protein	GgaAffx.23789.1.S1_at	peptidase (mitochondrial processing) alpha	0.489
Mitochondrial protein	Gga.6085.3.S1_a_at	mitochondrial ribosomal protein S6	0.444
Mitochondrial protein	GgaAffx.1241.1.S1_at	mitochondrial ribosomal protein S31	0.444
Mitochondrial protein	GgaAffx.2318.1.S1_at	similar to Translocase of outer mitochondrial membrane 20 homolog (yeast)	0.443
Mitochondrial protein	GgaAffx.194.1.S1_at	mitochondrial ribosomal protein L3	0.411
Mitochondrial protein	GgaAffx.9649.1.S1_at	solute carrier family 25 (mitochondrial carrier, brain), member 14	0.405
Mitochondrial protein	GgaAffx.9403.1.S1_s_at	mitochondrial translational initiation factor 3	0.391
Mitochondrial protein	Gga.4006.2.S1_a_at	mitochondrial ribosomal protein L39	0.380
Mitochondrial protein	GgaAffx.23494.1.S1_s_at	ATP synthase, H+ transporting, mitochondrial F0 complex, subunit F2	0.319
Protein degradation	Gga.1111.1.S1_a_at	ubiquitin specific peptidase 18	3.656
Protein degradation	GgaAffx.26411.1.S1_s_at	proteasome (prosome, macropain) 26S subunit, non-ATPase, 6	0.487
Protein degradation	GgaAffx.25545.1.S1_at	proteasome (prosome, macropain) subunit, beta type, 2	0.467
Protein degradation	GgaAffx.5008.1.S1_at	ubiquitin specific peptidase 34	0.413
Protein degradation	GgaAffx.7526.1.S1_at	similar to cullin 4A	0.411
Protein degradation	GgaAffx.26602.1.S1_at	proteasome (prosome, macropain) 26S subunit, ATPase, 6	0.400
Protein degradation	Gga.4152.1.S1_s_at	proteasome (prosome, macropain) subunit, alpha type, 1	0.382
Protein degradation	GgaAffx.9792.1.S1_at	cullin-associated and neddylation-dissociated 2 (putative)	0.373
Protein degradation	GgaAffx.4984.2.S1_s_at	proteasome (prosome, macropain) 26S subunit, non-ATPase, 1	0.274
Protein modification	Gga.6553.1.S1_at	cathepsin G	12.681
Protein modification	Gga.5393.1.S1_at	similar to serine protease	12.146
Protein modification	Gga.3867.1.S1_at	prion protein (p27-30) (Creutzfeldt-Jakob disease)	10.834
Protein modification	Gga.18980.1.S1_at	NUAK family, SNF1-like kinase, 1	10.073
Protein modification	Gga.7788.1.S1_at	similar to phospholipase A2 inhibitor gamma subunit B	9.230
Protein modification	GgaAffx.5597.1.S1_at	mitogen-activated protein kinase kinase kinase kinase 4	9.224
Protein modification	Gga.11824.1.S1_at	Cystatin F (leukocystatin)	8.876
Protein modification	Gga.606.1.S1_at	v-kit Hardy-Zuckerman 4 feline sarcoma viral oncogene homolog	8.172
Protein modification	GgaAffx.20295.1.S1_at	NUAK family, SNF1-like kinase, 1	8.084
Protein modification	Gga.415.1.S1_at	serpin peptidase inhibitor, clade B (ovalbumin), member 10	8.026
Protein modification	Gga.1193.1.S2_at	protein tyrosine phosphatase, receptor type, C	7.156
Protein modification	Gga.1193.1.S1_at	protein tyrosine phosphatase, receptor type, C	6.665
Protein modification	Gga.1148.1.S1_at	ST6 beta-galactosamide alpha-2,6-sialyltranferase 1	6.428
Protein modification	Gga.11016.1.S1_at	B locus M alpha chain 1	6.345
Protein modification	Gga.16596.1.S1_at	phosphotyrosine interaction domain containing 1	6.342
Protein modification	GgaAffx.11885.1.S1_s_at	Bruton agammaglobulinemia tyrosine kinase	6.278
Protein modification	Gga.5273.1.S1_at	Adrenergic, beta, receptor kinase 2	6.103
Protein modification	Gga.1148.1.S2_at	ST6 beta-galactosamide alpha-2,6-sialyltranferase 1	5.965
Protein modification	Gga.11456.1.S1_at	cystatin A (stefin A)	5.810
Protein modification	Gga.3379.2.S1_at	SH3-domain kinase binding protein 1	5.403
Protein modification	Gga.4105.2.S1_a_at	serpin peptidase inhibitor, clade E (nexin, plasminogen activator inhibitor type 1), member 2	5.271
Protein modification	GgaAffx.7823.1.S1_s_at	serine/threonine kinase 17a	5.240
Protein modification	Gga.12533.1.S1_at	protein tyrosine phosphatase-like A domain containing 2	5.231
Protein modification	Gga.6964.1.S1_at	protein kinase C, beta	5.112
Protein modification	GgaAffx.8960.1.S1_at	TXK tyrosine kinase	5.110
Protein modification	GgaAffx.26700.1.S1_s_at	protein kinase C, beta	4.995
Protein modification	GgaAffx.8628.1.S1_at	granzyme K (granzyme 3; tryptase II)	4.983
Protein modification	GgaAffx.12547.1.S1_at	ST3 beta-galactoside alpha-2,3-sialyltransferase 2	4.680
Protein modification	Gga.1035.1.S1_at	transglutaminase 2 (C polypeptide, protein-glutamine-gamma-glutamyltransferase)	4.648
Protein modification	GgaAffx.968.3.S1_s_at	zeta-chain (TCR) associated protein kinase 70kDa	4.625
Protein modification	Gga.7157.1.S1_at	protein kinase C, beta	4.554
Protein modification	GgaAffx.12557.1.S1_s_at	tyrosylprotein sulfotransferase 2	4.469
Protein modification	Gga.9012.1.S1_at	serine/threonine kinase 17b	4.276
Protein modification	Gga.11865.1.S1_at	similar to Cathepsin L, a	4.073
Protein modification	GgaAffx.6712.2.S1_s_at	protein kinase D3	3.941
Protein modification	Gga.6713.1.S1_at	granzyme A (granzyme 1, cytotoxic T-lymphocyte-associated serine esterase 3)	3.829
Protein modification	Gga.7011.1.S1_a_at	similar to cAMP-dependent protein kinase inhibitor gamma	3.823
Protein modification	Gga.8329.1.S1_at	protein kinase C, eta	3.815
Protein modification	GgaAffx.11653.1.S1_s_at	serine/threonine kinase 17b	3.802
Protein modification	Gga.2435.1.S2_at	Janus kinase 1 (a protein tyrosine kinase)	3.532
Protein modification	Gga.12120.1.S1_at	protein kinase C and casein kinase substrate in neurons 3	3.491
Protein modification	Gga.11468.1.S1_a_at	peptidylprolyl isomerase C (cyclophilin C)	3.474
Protein modification	Gga.19289.1.S1_at	Prolylcarboxypeptidase (angiotensinase C)	3.378
Protein modification	GgaAffx.6712.1.S1_at	protein kinase D3	3.231
Protein modification	GgaAffx.378.1.S1_at	protein tyrosine phosphatase, non-receptor type 7	3.158
Protein modification	GgaAffx.12579.1.S1_at	dual specificity phosphatase 10	3.107
Protein modification	GgaAffx.22748.1.S1_at	mitogen-activated protein kinase kinase kinase kinase 4	3.033
Protein modification	GgaAffx.9707.1.S1_s_at	protein-L-isoaspartate (D-aspartate) O-methyltransferase domain containing 1	3.012
Protein modification	Gga.536.1.S1_a_at	2'-5'-oligoadenylate synthetase-like	3.000

Protein modification	GgaAffx.22730.1.S1_at	phosphatase and tensin homolog (mutated in multiple advanced cancers 1), pseudogene 1	0.499
Protein modification	Gga.19366.1.S1_s_at	CDC-like kinase 4	0.499
Protein modification	GgaAffx.1012.1.S1_at	Tousled-like kinase 1	0.476
Protein modification	GgaAffx.25489.1.S1_at	TBC domain-containing protein kinase-like	0.469
Protein modification	GgaAffx.10968.1.S1_at	peptidylprolyl isomerase domain and WD repeat containing 1	0.465
Protein modification	Gga.16991.2.S1_a_at	Phosphatase and tensin homolog	0.462
Protein modification	GgaAffx.6526.1.S1_s_at	peptidylprolyl isomerase E (cyclophilin E)	0.455
Protein modification	Gga.9011.2.S1_at	protein phosphatase 1, regulatory (inhibitor) subunit 12A	0.440
Protein modification	Gga.7118.2.S1_s_at	protein phosphatase 3 (formerly 2B), catalytic subunit, beta isoform	0.439
Protein modification	GgaAffx.9058.2.S1_s_at	protein phosphatase 1, regulatory (inhibitor) subunit 8	0.436
Protein modification	GgaAffx.1184.1.S1_at	mitogen-activated protein kinase kinase kinase 4	0.433
Protein modification	Gga.5698.1.S1_at	methyl-CpG binding domain protein 5	0.418
Protein modification	Gga.267.1.S1_at	protein-L-isoaspartate (D-aspartate) O-methyltransferase	0.416
Protein modification	GgaAffx.24899.1.S1_at	integrin-linked kinase-associated serine/threonine phosphatase 2C	0.407
Protein modification	GgaAffx.23800.1.S1_at	pitrilysin metallopeptidase 1	0.406
Protein modification	Gga.19278.1.S1_s_at	protein arginine methyltransferase 7	0.401
Protein modification	Gga.6118.1.S1_at	serine/threonine kinase receptor associated protein	0.399
Protein modification	GgaAffx.2219.3.S1_s_at	cathepsin Z	0.395
Protein modification	GgaAffx.8894.1.S1_at	calcium/calmodulin-dependent serine protein kinase (MAGUK family)	0.392
Protein modification	Gga.18973.1.S1_at	Megakaryocyte-associated tyrosine kinase	0.387
Protein modification	GgaAffx.25618.1.S1_at	protein phosphatase 1, regulatory (inhibitor) subunit 1B	0.359
Protein modification	Gga.16586.1.S1_at	protein phosphatase 1, regulatory (inhibitor) subunit 13B	0.357
Protein modification	Gga.9700.4.S1_a_at	serine/threonine kinase 38 like	0.314
Protein modification	Gga.51.1.S1_at	ariadne homolog, ubiquitin-conjugating enzyme E2 binding protein, 1 (Drosophila)	0.294
Protein modification	GgaAffx.25266.1.S1_at	protein kinase C, alpha	0.282
Signal transduction	Gga.8030.1.S1_at	tissue factor pathway inhibitor 2	19.735
Signal transduction	Gga.3259.1.S1_at	angiopoietin 2	15.883
Signal transduction	GgaAffx.13210.1.S1_at	Src-like-adaptor	15.548
Signal transduction	GgaAffx.12646.1.S1_at	spleen tyrosine kinase	15.136
Signal transduction	Gga.4070.1.S1_at	Colony stimulating factor 1 receptor	13.456
Signal transduction	Gga.10829.1.S1_s_at	endothelin receptor type B	13.023
Signal transduction	Gga.15362.1.S1_at	regulator of G-protein signalling 18	12.172
Signal transduction	Gga.5008.1.S1_at	cyclin-dependent kinase inhibitor 2A (melanoma, p16, inhibits CDK4)	12.162
Signal transduction	Gga.7838.1.S1_at	colony stimulating factor 2 receptor, alpha, low-affinity (granulocyte-macrophage)	11.274
Signal transduction	Gga.1287.1.S1_at	RAS guanyl releasing protein 3 (calcium and DAG-regulated)	11.087
Signal transduction	GgaAffx.11475.1.S1_s_at	Ras association (RalGDS/AF-6) domain family member 2	10.759
Signal transduction	GgaAffx.13138.1.S1_at	tumor necrosis factor (ligand) superfamily, member 13b	10.559
Signal transduction	Gga.1348.1.S1_at	Rho GTPase activating protein 24	10.525
Signal transduction	Gga.9882.1.S1_at	colony stimulating factor 2 receptor, alpha, low-affinity (granulocyte-macrophage)	10.007
Signal transduction	Gga.8508.1.S2_s_at	Src-like-adaptor	9.750
Signal transduction	Gga.3306.1.S1_s_at	endothelin receptor type B	9.680
Signal transduction	Gga.10418.1.S1_at	Guanine nucleotide binding protein (G protein), gamma 10	9.372
Signal transduction	Gga.18933.1.S1_s_at	Ras association (RalGDS/AF-6) domain family 5	9.355
Signal transduction	GgaAffx.13005.1.S1_at	purinergic receptor P2Y, G-protein coupled, 8	9.317
Signal transduction	GgaAffx.7881.1.S1_s_at	Rho GTPase activating protein 15	9.272
Signal transduction	Gga.1585.1.S2_at	stathmin-like 2	9.250
Signal transduction	Gga.8164.1.S1_a_at	similar to Ras-related protein Rab-10	8.807
Signal transduction	GgaAffx.11864.1.S1_at	G protein-coupled receptor 174	8.430
Signal transduction	Gga.8789.1.S1_at	phosphoinositide-3-kinase, catalytic, delta polypeptide	8.124
Signal transduction	Gga.18945.1.S1_at	phosphatidylinositol-3,4,5-trisphosphate-dependent Rac exchange factor 1	8.070
Signal transduction	Gga.9880.1.S1_at	Purinergic receptor P2Y, G-protein coupled, 13	8.046
Signal transduction	Gga.7334.1.S1_at	amyloid beta (A4) precursor protein-binding, family B, member 1 interacting protein	7.585
Signal transduction	GgaAffx.21779.1.S1_s_at	stathmin-like 2	7.539
Signal transduction	Gga.10018.1.S1_at	Ras and Rab interactor 3	7.497
Signal transduction	GgaAffx.12475.1.S1_at	TNF receptor-associated factor 5	7.471
Signal transduction	GgaAffx.12029.1.S1_at	tumor necrosis factor receptor superfamily, member 1B	7.459
Signal transduction	GgaAffx.12969.1.S1_at	phosphoinositide-3-kinase, regulatory subunit 5, p101	7.271
Signal transduction	GgaAffx.11713.1.S1_s_at	Rho guanine nucleotide exchange factor (GEF) 3	7.242
Signal transduction	Gga.2696.1.S1_at	similar to selectin ligand interactor cytoplasmic-1	7.207
Signal transduction	GgaAffx.12948.1.S1_s_at	RAS guanyl releasing protein 3 (calcium and DAG-regulated)	7.062
Signal transduction	GgaAffx.13096.1.S1_at	Ras association (RalGDS/AF-6) domain family 5	7.014
Signal transduction	Gga.4359.1.S1_s_at	tumor necrosis factor (ligand) superfamily, member 13b	6.985
Signal transduction	GgaAffx.26579.1.S1_s_at	Colony stimulating factor 1 receptor	6.896
Signal transduction	Gga.539.1.S1_at	CD247 molecule	6.880
Signal transduction	Gga.8094.1.S1_a_at	Rho GDP dissociation inhibitor (GDI) beta	6.646

Signal transduction	Gga.6239.1.S1_at	regulator of G-protein signalling 1	6.607
Signal transduction	Gga.11560.2.S1_a_at	phosphoinositide-3-kinase adaptor protein 1	6.604
Signal transduction	Gga.6387.1.S1_at	ras-related C3 botulinum toxin substrate 2 (rho family, small GTP binding protein Rac2)	6.596
Signal transduction	GgaAffx.6562.1.S1_at	G protein-coupled receptor 171	6.591
Signal transduction	GgaAffx.10679.1.S1_at	colony stimulating factor 2 receptor, alpha, low-affinity (granulocyte-macrophage)	6.521
Signal transduction	Gga.3154.1.S1_at	ras homolog gene family, member Q	6.330
Signal transduction	Gga.5148.1.S2_s_at	tumor necrosis factor receptor superfamily, member 1B	6.305
Signal transduction	GgaAffx.6562.1.A1_at	G protein-coupled receptor 171	6.256
Signal transduction	GgaAffx.11426.1.S1_at	tumor necrosis factor receptor superfamily, member 18	6.131
Signal transduction	Gga.5148.1.S1_at	tumor necrosis factor receptor superfamily, member 1B	5.991
Signal transduction	Gga.8823.1.S1_s_at	CKLF-like MARVEL transmembrane domain containing 7	5.990
Signal transduction	GgaAffx.3477.1.S1_at	G protein-coupled receptor 35	5.986
Signal transduction	GgaAffx.12575.1.S1_s_at	ankyrin repeat and SOCS box-containing 9	5.942
Signal transduction	Gga.1027.1.S1_at	similar to Rho-GTPase-activating protein 6 (Rho-type GTPase-activating protein RhoGAPX-1)	5.775
Signal transduction	Gga.998.2.S1_a_at	CD3d molecule, delta (CD3-TCR complex)	5.712
Signal transduction	Gga.11395.1.S1_at	G protein-coupled receptor 34	5.576
Signal transduction	GgaAffx.24611.2.S1_s_at	similar to histamine H3 receptor H3S	5.551
Signal transduction	GgaAffx.4780.1.S1_at	signal transducer and activator of transcription 1, 91kDa	5.283
Signal transduction	GgaAffx.12684.1.S1_s_at	Rho GTPase activating protein 25	5.257
Signal transduction	Gga.11516.1.S1_at	Pyrimidinergic receptor P2Y, G-protein coupled, 6	5.235
Signal transduction	Gga.4359.1.S2_s_at	tumor necrosis factor (ligand) superfamily, member 13b	5.162
Signal transduction	GgaAffx.12918.1.S1_s_at	cytohesin 4	5.126
Signal transduction	Gga.4970.1.S1_at	CD3e molecule, epsilon (CD3-TCR complex)	4.995
Signal transduction	Gga.6098.1.S1_at	Similar to contactin associated protein	4.976
Signal transduction	GgaAffx.11771.1.S1_at	Rac/Cdc42 guanine nucleotide exchange factor (GEF) 6	4.796
Signal transduction	Gga.352.1.S1_at	regulator of G-protein signaling 20	4.700
Signal transduction	Gga.10030.1.S1_s_at	phosphoinositide-3-kinase, regulatory subunit 5, p101	4.610
Signal transduction	Gga.986.1.S1_s_at	tumor necrosis factor receptor superfamily, member 18	4.476
Signal transduction	GgaAffx.8756.1.S1_at	similar to FKSG15	4.405
Signal transduction	GgaAffx.10807.1.S1_at	Epstein-Barr virus induced gene 2 (lymphocyte-specific G protein-coupled receptor)	4.287
Signal transduction	Gga.1092.1.S1_s_at	G protein-coupled receptor 174	4.282
Signal transduction	Gga.3624.1.S1_at	phosphodiesterase 3B, cGMP-inhibited	3.977
Signal transduction	GgaAffx.12014.1.S1_s_at	amyloid beta (A4) precursor protein-binding, family B, member 1 interacting protein	3.879
Signal transduction	Gga.4113.1.S1_at	ras homolog gene family, member F (in filopodia)	3.743
Signal transduction	GgaAffx.11590.1.S1_s_at	cytohesin 1	3.702
Signal transduction	Gga.5148.1.S1_s_at	tumor necrosis factor receptor superfamily, member 1B	3.601
Signal transduction	Gga.8752.1.S1_s_at	sorting nexin 10	3.594
Signal transduction	GgaAffx.3442.2.S1_s_at	Rho guanine nucleotide exchange factor (GEF) 3	3.579
Signal transduction	GgaAffx.9008.1.S1_s_at	parvin, gamma	3.410
Signal transduction	Gga.17410.1.S1_at	Glucocorticoid induced transcript 1	3.362
Signal transduction	Gga.9999.1.S1_at	RAS p21 protein activator 3	3.341
Signal transduction	GgaAffx.11861.1.S1_at	RasGEF domain family, member 1A	3.313
Signal transduction	Gga.19099.2.S1_a_at	secreted protein, acidic, cysteine-rich (osteonectin)	3.312
Signal transduction	GgaAffx.9971.2.S1_s_at	G protein-coupled receptor kinase 4	3.309
Signal transduction	Gga.10465.1.S1_s_at	G protein-coupled receptor 65	3.259
Signal transduction	Gga.12335.2.S1_s_at	cytohesin 4	3.246
Signal transduction	Gga.12662.1.S1_at	signal transducer and activator of transcription 1, 91kDa	3.235
Signal transduction	Gga.3175.1.S1_at	Phosphoinositide-3-kinase, regulatory subunit 1 (p85 alpha)	3.223
Signal transduction	GgaAffx.11756.1.S1_at	G protein-coupled receptor 137B	3.153
Signal transduction	GgaAffx.1027.1.S1_at	TNF receptor-associated factor 1	3.135
Signal transduction	GgaAffx.7443.1.S1_at	signal transducing adaptor family member 1	3.053
Signal transduction	GgaAffx.1691.1.A1_at	regulator of G-protein signalling 5	3.029
Signal transduction	GgaAffx.5618.1.S1_at	amyloid beta (A4) precursor protein-binding, family B, member 1 interacting protein	0.499
Signal transduction	Gga.7708.1.S1_at	agrin	0.497
Signal transduction	Gga.4589.1.S3_at	thyroid hormone receptor interactor 12	0.496
Signal transduction	GgaAffx.22041.1.S1_s_at	src kinase associated phosphoprotein 2	0.476
Signal transduction	GgaAffx.25488.1.S1_at	TNF receptor-associated factor 6	0.467
Signal transduction	Gga.4101.1.S1_s_at	diacylglycerol kinase, delta 130kDa	0.463
Signal transduction	Gga.10747.1.S1_s_at	ankyrin 3	0.461
Signal transduction	Gga.2166.3.S1_a_at	hepatocyte growth factor (hepapoietin A; scatter factor)	0.458
Signal transduction	GgaAffx.21433.1.S1_s_at	RAS, dexamethasone-induced 1	0.457
Signal transduction	Gga.7797.1.S1_s_at	Rac GTPase activating protein 1	0.427
Signal transduction	GgaAffx.23134.1.S1_at	insulin receptor substrate 1	0.387
Signal transduction	Gga.8230.1.S1_at	discs, large homolog 7 (Drosophila)	0.384
Signal transduction	GgaAffx.25114.2.S1_s_at	lipopolysaccharide-induced TNF factor	0.320
Transporter/membrane trafficking	Gga.2680.1.S1_at	aquaporin 1 (Colton blood group)	19.758
Transporter/membrane trafficking	Gga.918.1.S1_at	potassium large conductance calcium-activated channel, subfamily M, beta member 1	11.604
Transporter/membrane trafficking	Gga.9077.1.S1_at	hydrogen voltage-gated channel 1	9.945

Transporter/membrane trafficking	Gga.11184.1.S1_at	potassium channel tetramerisation domain containing 12	9.018
Transporter/membrane trafficking	Gga.12076.1.S1_at	purinergic receptor P2X, ligand-gated ion channel, 5	5.915
Transporter/membrane trafficking	Gga.871.1.S1_at	solute carrier family 2 (facilitated glucose transporter), member 3	5.714
Transporter/membrane trafficking	Gga.14445.1.S1_s_at	solute carrier family 1 (high affinity aspartate/glutamate transporter), member 6	5.374
Transporter/membrane trafficking	Gga.16544.1.S1_s_at	chloride intracellular channel 2	5.361
Transporter/membrane trafficking	Gga.1235.2.S1_at	potassium voltage-gated channel, shaker-related subfamily, beta member 2	5.274
Transporter/membrane trafficking	Gga.7278.1.S1_at	Solute carrier family 25, member 29	4.881
Transporter/membrane trafficking	GgaAffx.10727.2.S1_at	ATPase, class V, type 10A	4.785
Transporter/membrane trafficking	Gga.6246.1.S1_at	Rab interacting lysosomal protein-like 2	4.724
Transporter/membrane trafficking	Gga.7430.1.S1_at	synaptotagmin XI	4.698
Transporter/membrane trafficking	GgaAffx.8046.1.S1_s_at	solute carrier family 41, member 2	4.674
Transporter/membrane trafficking	GgaAffx.5539.1.S1_s_at	solute carrier family 1 (glutamate/neutral amino acid transporter), member 4	4.495
Transporter/membrane trafficking	Gga.17590.1.S1_s_at	chloride intracellular channel 2	4.422
Transporter/membrane trafficking	Gga.4594.1.S2_at	ATPase, Na+/K+ transporting, beta 3 polypeptide	4.070
Transporter/membrane trafficking	Gga.4451.1.S1_at	gelsolin	3.981
Transporter/membrane trafficking	GgaAffx.1584.1.S1_at	solute carrier family 9 (sodium/hydrogen exchanger), member 9	3.831
Transporter/membrane trafficking	GgaAffx.1584.2.S1_s_at	solute carrier family 9 (sodium/hydrogen exchanger), member 9	3.608
Transporter/membrane trafficking	Gga.9127.1.S1_at	ATPase, class V, type 10D	3.476
Transporter/membrane trafficking	Gga.9816.1.S1_s_at	chloride intracellular channel 2	3.436
Transporter/membrane trafficking	Gga.1235.1.S1_at	potassium voltage-gated channel, shaker-related subfamily, beta member 2	3.434
Transporter/membrane trafficking	Gga.4594.1.S1_at	ATPase, Na+/K+ transporting, beta 3 polypeptide	3.420
Transporter/membrane trafficking	Gga.6941.1.S1_at	Similar to guanine nucleotide exchange factor Lbc	3.374
Transporter/membrane trafficking	Gga.2564.1.S1_s_at	adaptor-related protein complex 1, sigma 2 subunit	3.276
Transporter/membrane trafficking	GgaAffx.22282.1.S1_at	solute carrier family 29 (nucleoside transporters), member 1	0.497
Transporter/membrane trafficking	Gga.458.1.S1_s_at	ATPase, Ca++ transporting, plasma membrane 4	0.488
Transporter/membrane trafficking	Gga.18339.1.S1_s_at	nucleoporin 54kDa	0.488
Transporter/membrane trafficking	Gga.5104.1.S1_at	nucleoporin 35kDa	0.480
Transporter/membrane trafficking	Gga.7586.1.S1_at	solute carrier family 23 (nucleobase transporters), member 2	0.474
Transporter/membrane trafficking	GgaAffx.1444.1.S1_at	karyopherin alpha 5 (importin alpha 6)	0.468
Transporter/membrane trafficking	GgaAffx.22855.1.S1_at	RAB14, member RAS oncogene family	0.467
Transporter/membrane trafficking	Gga.6181.1.S1_at	potassium channel tetramerisation domain containing 10	0.461
Transporter/membrane trafficking	GgaAffx.26684.1.S1_at	solute carrier family 16, member 13 (monocarboxylic acid transporter 13)	0.451
Transporter/membrane trafficking	GgaAffx.24196.1.S1_s_at	potassium channel tetramerisation domain containing 20	0.448
Transporter/membrane trafficking	GgaAffx.10181.1.S1_at	syntaxin binding protein 3	0.430
Transporter/membrane trafficking	GgaAffx.4428.2.S1_s_at	vacuolar protein sorting 53 homolog (S. cerevisiae)	0.424
Transporter/membrane trafficking	GgaAffx.6526.7.S1_s_at	importin 7	0.421
Transporter/membrane trafficking	Gga.12255.1.S1_s_at	ATPase, H+ transporting, lysosomal V0 subunit a1	0.413
Transporter/membrane trafficking	Gga.16527.1.S1_at	solute carrier family 43, member 2	0.412
Transporter/membrane trafficking	Gga.13215.1.S1_at	RAB, member RAS oncogene family-like 5	0.396
Transporter/membrane trafficking	Gga.7931.1.S1_at	nucleoporin 133kDa	0.271
