# Supplementary material for: Transcriptome Analysis in Chicken Cecal Epithelia upon Infection by Eimeria tenella In Vivo
Source: PLoS One. 2013 May 30;8(5):e64236. doi: 10.1371/journal.pone.0064236 (PMC3667848; doi:10.1371/journal.pone.0064236)
Supplement: Table S5 — List of primers used in real-time qRT-PCR analysis. (PDF) [file pone.0064236.s007.pdf]

Additional file 6, Table S5. List of primers used in real-time qRT-PCR analysis

Accession No.	Gene Symbol		Primer Sequence (5'-3')
NM_204649.1	CD4	F	AGCTGTGTGTTTGCGGTCATC
		R	ATTTATGGCTTTGCAGCTCAGG
NM_204891.1	ATP2A3	F	ACCTGCAGGACTCCAGAGCAA
		R	GCAAACCCTCAGTCCGAGTTACA
NM_205036.1	TGFBI	F	CACGAACAGCATTCAGCACATC
		R	TGAGGTCAGAAGCAGCCACAG
NM_205422.1	P20K	F	GGAGCGGAACTACACGGATGA
		R	GCCTCGAGCTTTGGCACATTA
NM_001007488.3	TLR6	F	TGCAGTAGTCATCTGTTGGCTCTT
		R	AGGGATCCCATTATGTGTCTCA
XM_417175.2	MMP3	F	GCCCAGATGCGGAATACCAG
		R	TTTGCAATTGCTTCGTCCACA
XM_001231298.1	STAT4	F	AATCAGCAAATGGGAGCCTGTC
		R	CTGCGTTTCAAAGCTGATGGAG
XM_001234442.1	MAPK13	F	GCTTGAGGACAAGGCGGCTA
		R	ACTGCCTGAGGATTGGCTGTG
NM_205518.1	ACTB	F	ATTGTCCACCGCAAATGCTTC
		R	AAATAAAGCCATGCCAATCTCGTC
NM_001007942	RSFR	F	AATCCTCGCAAATACCCAAAGA
		R	AAGCTGCATTGCTTGGGAAC
NM_205251	ITGB2	F	CACTGACTGACAATGCCGAGAA
		R	ATTGCGCCAGCCAATCAAG
NM_001030704	MMD	F	GGAGTCCATGGCTGTTGGTTC
		R	CCAGAATGACAAGTGACGCTGTATC
NM_001039329	PRDX6	F	GATGAGATCCTGAGAGTGGTGGAC
		R	TCATCAGGTAAGGTGGGCACAA
NM_001012600	PSMD1	F	GCCCTTATCATGATCCAGCAGAC
		R	CGCTCCAAACTTGGCCATAAC
NM_001006366	MRPL3	F	CAGGCGTCCTGGAGCAATATCTA
		R	AATTTGTGTGACCCGGAACAGAG

NM_001007824	Actin, gamma 1	F	GGAGCGCAAGTATTCTGTCTGG
		R	GCACTTGCGATGGACTATGGA
XM_001234262	ADH1C	F	TGAAGTGCTGACTGAAATGACTGG
		R	CGTAGTTGTTGTGGCAAGAGGC
NM_001030870	NADK	F	AAGAGCACAGAGAGAAGATGACGG
		R	GGAAAGGTAAGAAGAAGGACCACG
FM165414	18S rRNA	F	CGGTTCTATTTTGTTGGTTTTCGG
		R	GCTTTCGCTTTAGTTCGTCTTGC
XM_416918.	SLC9A2	F	TCAGCAAGTACTTCGTCCCTGTG
		R	GTCAGATTTCAGGTGGCTCTGCTA
XM_418767	DYNC1LI1	F	GAATGTGCATGATGAAGATCGAGA
		R	CCTTTAATGAGTTTGCCTCCATTG
